# Rapid evolution of α-gliadin gene family revealed by analyzing *Gli-2* locus regions of wild emmer wheat

**DOI:** 10.1007/s10142-019-00686-z

**Published:** 2019-06-13

**Authors:** Naxin Huo, Tingting Zhu, Shengli Zhang, Toni Mohr, Ming-Cheng Luo, Jong-Yeol Lee, Assaf Distelfeld, Susan Altenbach, Yong Q. Gu

**Affiliations:** 1 United States Department of Agriculture-Agricultural Research Service USDA-ARS, Western Regional Research Center, 800 Buchanan Street, Albany, CA 94710 USA; 2 Department of Plant Sciences, University of California, Davis, CA 95616 USA; 3 Hena Institute of Science and Technology, Xinxiang, Hena Province, 453003 China; 4 National Institute of Agricultural Sciences, RDA, Jeonju, 54874 South Korea; 5 Institute for Crop Improvement, Tel Aviv University, Tel Aviv-Yafo, Israel

**Keywords:** Wheat gluten proteins, α-Gliadin gene family, Gene duplication, Genome evolution, Phylogeny, Celiac disease

## Abstract

α-Gliadins are a major group of gluten proteins in wheat flour that contribute to the end-use properties for food processing and contain major immunogenic epitopes that can cause serious health-related issues including celiac disease (CD). α-Gliadins are also the youngest group of gluten proteins and are encoded by a large gene family. The majority of the gene family members evolved independently in the A, B, and D genomes of different wheat species after their separation from a common ancestral species. To gain insights into the origin and evolution of these complex genes, the genomic regions of the *Gli-2* loci encoding α-gliadins were characterized from the tetraploid wild emmer, a progenitor of hexaploid bread wheat that contributed the AABB genomes. Genomic sequences of *Gli-2* locus regions for the wild emmer A and B genomes were first reconstructed using the genome sequence scaffolds along with optical genome maps. A total of 24 and 16 α-gliadin genes were identified for the A and B genome regions, respectively. α-Gliadin pseudogene frequencies of 86% for the A genome and 69% for the B genome were primarily caused by C to T substitutions in the highly abundant glutamine codons, resulting in the generation of premature stop codons. Comparison with the homologous regions from the hexaploid wheat cv. Chinese Spring indicated considerable sequence divergence of the two A genomes at the genomic level. In comparison, conserved regions between the two B genomes were identified that included α-gliadin pseudogenes containing shared nested TE insertions. Analyses of the genomic organization and phylogenetic tree reconstruction indicate that although orthologous gene pairs derived from speciation were present, large portions of α-gliadin genes were likely derived from differential gene duplications or deletions after the separation of the homologous wheat genomes ~ 0.5 MYA. The higher number of full-length intact α-gliadin genes in hexaploid wheat than that in wild emmer suggests that human selection through domestication might have an impact on α-gliadin evolution. Our study provides insights into the rapid and dynamic evolution of genomic regions harboring the α-gliadin genes in wheat.

**Electronic supplementary material:**

The online version of this article (10.1007/s10142-019-00686-z) contains supplementary material, which is available to authorized users.

## Introduction

Bread wheat is one of the most important food crops in the world, providing about 20% of the calories in the human diet. Wheat is the most diversely adapted cereal crop and is grown in a wide range of temperate environments, from 67° N in Scandinavia and Russian to 45° S in Argentina (Peng et al. 2011). Bread wheat also provides a high yield production, up to 15 tons per ha in cool wet environments. Although the adaptability and high yields of wheat contribute to its success as an important food crop, there is no doubt that the unique properties of wheat flour that allow it to be processed into a range of food products provide advantages over other cereals (Shewry 2009). These unique properties are determined by the structures and interactions of gluten proteins which comprise about 75% of the total proteins stored in wheat grain. Wheat gluten consists of two types of proteins, the monomeric gliadins and polymeric glutenins. They have different functionalities in determining the viscoelastic properties of wheat flour dough. Glutenins confer elasticity while gliadins provide extensibility (Shewry et al. 2002). The glutenins are subdivided into HMW-glutenins and LMW-glutenins while the gliadins are subdivided into α, γ, ω, and δ-gliadins. Genetic and genomic studies indicate that genes encoding these gluten proteins are primarily located in three genomic regions. HMW-glutenin genes are located at the *Glu-1* loci on the long arms of the wheat group 1 homoeologous chromosomes (Gu et al. 2004), while on the short arms of the same chromosome, there are two tightly linked *Glu-3* and *Gli-1* loci encoding the LMW-glutenins and γ-, δ-, and ω-gliadins, respectively (D'Ovidio and Masci 2004; Dong et al. 2016). The third genomic region located on the short arms of wheat group 6 chromosomes harbors the *Gli-2* loci encoding α-gliadins.

Studies on gluten gene evolution indicate that the HMW-glutenins originated from a duplication of an ancestral water-soluble globulin gene, which occurred ~ 25 MYA before the divergence of the Triticeae and Brachypodieae lineages (Gu et al. 2010; Xu and Messing 2009). Although the origin of LMW-glutenins is less clear, phylogenetic studies showed that sequences related to LMW-glutenins also exist in Brachypodium (Dong et al. 2016). Gliadins likely evolved after the occurrence of glutenins, particularly the α-gliadins that are not present in rye and barley, which diverged from wheat about 7 to 10 MYA. Therefore, α-gliadins are the youngest group of gluten proteins present in wheat and its ancestral species (Huo et al. 2018b). However, α-gliadins are important nutrition sources as they account for 15–30% of the total seed storage proteins in the wheat grain (Altenbach et al. 2011). Unfortunately, α-gliadins are also major triggers of celiac disease (CD), a food-sensitive autoimmune disorder that impacts about 0.7–2% of the human population worldwide (Sollid et al. 2012). Several major CD immunogenic peptides have been identified in wheat α-gliadins, including the most toxic 33-mer consisting of six overlapping copies of three highly stimulatory epitopes (Sollid et al. 2012). A breeding approach to develop wheat cultivars with reduced immunogenic potential, while retaining wheat flour’s end-use functional properties, has been proposed (Shewry and Tatham 2016). A comprehensive analysis of α-gliadin genomic regions could enhance the breeding effort by unraveling their genetic diversity and developing molecular markers for selections in breeding programs. However, the α-gliadin gene family is also the most complex among the gluten genes with previous estimates of copy numbers ranging from 25 to 150 in different wheat cultivars and ancestral species (Anderson and Greene 1997; Harberd et al. 1985). Furthermore, the generation of high-quality sequences to uncover α-gliadin genomic regions still represents a big challenge due to the clustering of multiple tandemly duplicated gene copies in addition to the large size (17 G), polyploid nature, and high content of repetitive DNA of the wheat genome. The origin and evolutionary relationships of α-gliadin gene family members are often difficult to draw without the context of their genomic organizations.

Recently, a high-quality genome sequence of the reference wheat cultivar Chinese Spring has been generated based on Illumina short reads (International Wheat Genome Sequencing Consortium et al. 2018) and used to identify a repository of grain proteins associated with wheat allergens and immunogenic responses (Juhasz et al. 2018). With the improved genome sequencing of Chinese Spring with PacBio long reads (Zimin et al. 2017) and utilization of BioNano genome maps to improve and validate the sequence assemblies, the complexities of the wheat gluten genomic regions have now been better resolved (Huo et al. 2018a; Huo et al. 2018b). In Chinese Spring (CS), the full complement of α-gliadin genes includes 47 genes with 26 encoding functional proteins and the rest being pseudogenes (Huo et al. 2018b). Comparative analyses of orthologous regions from the homoeologous A, B, and D genomes have greatly advanced our understanding of the mechanisms underlying the molecular evolution of the complex α-gliadin gene family members. Phylogenetic studies revealed that duplication and expansion of α-gliadin genes occurred 2 to 3 MYA after the divergence of the wheat A, B, and D genomes (Huo et al. 2018b). This suggests that the dynamic and rapid evolution of α-gliadin genes occurred recently and continually. The identification of a full complement of α-gliadins in CS also enabled a more accurate and robust examination of expression levels of individual genes using transcriptomic RNA-seq data (Huo et al. 2018b). In addition, the correlation of transcript levels with protein accumulation levels was examined recently in Chinese Spring using a proteomic approach (Altenbach et al. 2019). Understanding the genetic variation of α-gliadin genes and their protein accumulation is important as both the quality (coding sequence variation) and quantity (differential gene expression) of gluten proteins contribute to the dough properties of wheat flour from different cultivars (Shewry et al. 2002).

Domestication of wheat occurred about 10,000 years ago and played an important role in human civilization (Dubcovsky and Dvorak 2007). The initial domestication started with allotetraploid wild emmer wheat (*Triticum dicoccoides* (Korn.) Thell; genome AABB) and the subsequent evolution of certain domestication traits including non-brittle spike led to the cultivated tetraploid emmer wheat (*T. turgidum* ssp. *dicoccum*, 2n = 4x = 28, genome AABB). The hexaploid bread wheat (*T. aestivum*, 2n = 6x = 21, AABBDD) was formed from the hybridization of a domesticated emmer wheat with an ancestral diploid *Aegilops tauschii* (DD) genome. Therefore, the wild emmer wheat is the progenitor of both cultivated tetraploid pasta wheat and hexaploid bread wheat, contributing the AABB genomes to both cultivated wheat types. Although the analysis of gluten complements in ancestral wheat species has not been carried out in detail, studies have shown that different types of α-gliadin genes from diploid, tetraploid, and polyploid wheats differ considerably in the frequencies and in the presence and abundance of CD immunogenic peptides (Ozuna et al. 2015). A comprehensive study using a genomics approach to analyze the genomic regions harboring α-gliadin genes will not only elucidate α-gliadin evolution in ancestral wheat species, but also facilitate the exploitation of tetraploid emmer wheat to introduce useful traits into new varieties with enhanced end-use properties and reduced immunogenic potential. The genetic diversity in crop plants has been considerably eroded compared with their wild ancestors due to bottlenecks imposed by plant domestication and modern breeding (Huang et al. 2016; Peng et al. 2011). Therefore, modern cultivars are more vulnerable to various biotic and abiotic stresses. Natural populations of wild emmer wheat possess wide genotypic variation in many agronomically important traits, including resistances to biotic and abiotic stresses, seed size and yield, protein content and quantity including high grain protein, and novel gliadins and glutenins (Hebelstrup 2017; Huang et al. 2016; Merchuk-Ovnat et al. 2016). One of the major objectives in modern agriculture is to enrich the genetic diversity in germplasm by reintroducing valuable traits from wild ancestral species.

Because of its importance in the improvement of modern wheat varieties, a reference quality genome sequence of a wild emmer accession “Zavitan” was recently generated to serve as a useful resource for a wide range of applications (Avni et al. 2017). In this study, we reconstructed the genomic regions harboring the α-gliadin loci from the A and B genomes of wild emmer wheat using the genome sequence and BioNano genome map data. The high-quality sequences generated here allowed us to perform a detailed analysis to understand the genomic structure and organization of *Gli-2* regions in wild emmer wheat and compare them with the homologous regions from the hexaploid wheat cv. Chinese Spring to reveal more recent evolutionary changes including gene duplications and deletions, differences in gene expression, and abundance of CD immunogenic peptides.

## Materials and methods

### Sequence assembly of genomic regions harboring *Gli-2* loci in wild emmer

Sequence scaffolds from the wild emmer accession Zavitan were downloaded from the published data (Avni et al. 2017). α-Gliadin genes and genes annotated in the genomic regions carrying *Gli-2* loci from the hexaploid wheat cv. Chinese Spring (Huo et al. 2018b) were used to BLAST against the wild emmer sequence scaffolds. Previously, an optical BioNano genome map was generated for the wild emmer accession using the nicking endonuclease Nt.*Bsp*QI (Zhu et al. 2019). The retrieved sequence scaffolds were first digested in silico based on the restriction site of Nt.*Bsp*QI using the Knickers program (BioNano Genomics) and then aligned with the BioNano map by computing with RefAligner (BioNano Genomics). Visualization of the alignments was performed with snapshot in IrysView (BioNano Genomics). All software packages used can be obtained from BioNano Genomics (https://bionanogenomics.com/support/software-downloads/). Manual checking and editing were performed to improve the final assembly by aligning, merging, and reorienting contigs (Hastie et al. 2013).

### Sequence analysis and gene annotation

For sequence analysis and gene annotation, the final assembled α-gliadin genomic sequences for the wild emmer A and B genomes were first submitted to TriAnnot pipeline for automated gene annotation (Leroy et al. 2012). In addition, homology searches were performed against the NCBI non-redundant databases using BLASTN, BLASTX, and TBLASTX algorithms to verify annotated genes and identify missed genes and pseudogenes. Pseudogenes are usually excluded using automated gene annotation pipelines. However, they can be easily identified manually using homology-based BLAST searches. The annotated genes were then compared with the gene contents from the α-gliadin regions of the hexaploid wheat cv. Chinese Spring (Huo et al. 2018b). Nine annotated full-length intact α-gliadin gene sequences from wild emmer were deposited in NCBI GenBank with accession no. from MK333911 to MK333918 and MK358822. The reconstructed genomic sequences spanning the α-gliadin loci are freely available upon request.

Identification of repetitive DNA elements and their arrangements in the genome is important for better understanding of genomic structure and evolution. This is particularly true for the wheat genome with high repetitive DNA contents (~ 85%). Currently, no pipelines are available for accurate identification of repetitive DNA elements and delineation of their nested structure pattern. Therefore, manual annotation of repetitive DNA elements was performed using different tools including DNAstar MegAlign dotplot analysis (www.dnastar.com) and by blasting against and comparison with the TREP database (http://botserv2.uzh.ch/kelldata/trep-db/index.html).

### Transcriptome data analysis

The expression of α-gliadin genes from wild emmer was studied using Illumina RNA-seq transcriptome data generated from developing grain at 12 days post anthesis (Avni et al. 2017). The wild emmer coding sequences (CDS) were downloaded from https://www.dropbox.com/sh/3dm05grokhl0nbv/AAC3wvlYmAher8fY0srX3gX9a?dl=0. The annotated α-gliadin gene sequences along with the wild emmer CDS were used as a complete gene set for mapping illumina transcriptome reads using the CLC Genomic Workbench (v8.5) RNA-Seq Analysis Toolbox. Because of the high nucleotide similarities of α-gliadin CDS, stringent mapping parameters with mismatch cost 2, insertion and deletion cost 3, length fraction 0.9, and similarity 0.99 were employed in mapping of the reads. The FPKM values were calculated using the function in the CLC Toolbox. RNA-seq alignments were manually reviewed to confirm the assembly of α-gliadin gene sequences, including mutation sites causing pseudogenization.

### Phylogenetic tree analysis

For construction of phylogenetic trees, nucleotide sequences of α-gliadin gene coding regions were extracted and aligned using MUSCLE with default settings (Kumar et al. 2016), followed by visual inspection and manual editing to improve the sequence alignment qualities. Pseudogenes containing large deletions were removed, as they can be problematic in tree constructions. However, pseudogenes that were disrupted by TE insertions but contained the full-length gene sequences were included after removal of the TE sequences. Phylogenetic trees were constructed using the neighbor-joining method in the MEGA7 program with the confidence probability estimated using the bootstrap test with 1000 replications (Kumar et al. 2016).

## Results

### Reconstruction of α-gliadin gene genomic regions in wild emmer wheat

To reconstruct the α-gliadin gene genomic regions in wild emmer wheat, we first searched wild emmer sequence scaffolds using BLASTn with α-gliadin and glutamate-like receptor (*GLR*) gene sequences identified in the hexaploid wheat cv. Chinese spring. The *GLR* genes were included to define locus boundaries since the three homoeologous α-gliadin loci in Chinese Spring were all flanked by *GLR* genes (Huo et al. 2018b). A total of six sequence scaffolds with sizes ranging from 132 kb (scaffold46758) to 2.8 Mb (scaffold43817) were retrieved (Supplementary Table S1). One scaffold (scaffold1353-1) was from the B genome and the rest from the A genome. These scaffolds were used to align the BioNano genome (BNG) map previously generated for the wild emmer genome (Dvorak et al. 2018; Zhu et al. 2019) to identify BNG contigs. Only two BNG contigs, ctg6 and ctg46, were retrieved, with estimated sizes of 229 and 55 Mb, respectively. These two BNG contigs served as frameworks to reconstruct the α-gliadin genomic regions by aligning, ordering, and reorienting scaffolds using the method described previously (Huo et al. 2018a). Such a process can validate and improve sequence assemblies. For example, two scaffolds, scaffold46758 and scaffold48495, both containing multiple α-gliadin genes, were missing in the A genome α-gliadin region in the pseudomolecule assembly of the wild emmer genome. The BNG ctg6 showed a perfect alignment with the two sequence scaffolds and guided the placement of the unanchored scaffolds into the region (Supplementary Fig. S1).

### α-Gliadin intact genes and pseudogene analysis

In the reconstructed sequence regions of the wild emmer A and B genomes, four *GLR* genes were identified for each genome. Two (*GLR1* and *GLR2*) are in front of the α-gliadin genes, one (*GLR3*) is within the α-gliadin cluster, and the last (*GLR4*) is located after the α-gliadin gene region (Supplementary Table 2). In the orthologous α-gliadin regions of Chinese Spring A, B, and D and diploid *Ae. tauschii* genomes, the same number and order of *GLR* genes were identified (Huo et al. 2017; Huo et al. 2018b). Therefore, these *GLR* genes defined the boundaries of the α-gliadin locus regions for the wild emmer A and B genomes. The final sequence for the A genome region was ~ 2.89 Mb, while that for the B genome region, it was 0.91 Mb. We then performed detailed analyses on the sequences by employing manual annotation to identify both full-length intact genes and pseudogenes since automated gene annotation pipelines often do not predict pseudogenes that have been disrupted by various evolutionary events. This is particularly true for gliadin genes with large numbers of gene family members. There were 24 and 16 α-gliadin genes for the A and B genomes, respectively (see Supplementary Table S2 and Supplementary file 1). Twenty of 24 A genome (83%) and 11of 16 B genome (69%) α-gliadin genes were likely pseudogenes due to the presence of various mutation events, including deletions, transposable element insertions, mutations causing in-frame stop codons, frameshift mutations etc. (Table 1). Two genes, *Td-α-A6* and *Td-α-A19*, were not assembled completely because gaps were present in the assembled coding regions. In addition to the gaps, *Td-α-A6* also had a deletion at the 5′ end and *Td-α-A19* had an in-frame stop codon. Therefore, they were both likely pseudogenes.

**Table 1:** α-Gliadin genes and mutation events in the pseudogenes

Gene ID	Length (bp)*	Types of mutations	In-frame stop codon no.	Stop codons next to Q
*Td-α-A1*	924	Stop codon	2	1
*Td-α-A2*	879	Stop codon	2	2
*Td-α-A3*	785	Stop codon	1	1
*Td-α-A4*	859	Frameshift and stop codon	1	1
*Td-α-A5*	859	Frameshift and stop codon	3	2
*Td-α-A6*	583	Deletion at 5′ end, frameshift, and gap	0	0
*Td-α-A7*	696	Deletion at 5′ end, frameshift, and stop codon	2	1
*Td-α-A8*	859	Stop codon	4	2
*Td-α-A9*	846	Stop codon	2	2
*Td-α-A10*	834	Stop codon	2	1
*Td-α-A11*	837	Stop codon	1	1
*Td-α-A12*	845	Frameshift and stop codon	8	5
*Td-α-A13*	845	Stop codon	3	1
*Td-α-A14*	894	Intact full-length		
*Td-α-A15*	870	Stop codon	1	0
*Td-α-A16*	456	Deletion at 3′ end and stop codon	2	0
*Td-α-A17*	887	Intact full-length		
*Td-α-A18*	845	Frameshift and stop codon	5	1
*Td-α-A19*	579	Frameshift, stop codon, and gap	1	0
*Td-α-A20*	861	Intact full-length		
*Td-α-A21*	858	Intact full-length		
*Td-α-A22*	859	Deletion at 5′ end		
*Td-α-A23*	855	Stop codon	1	1
*Td-α-A24*	849	Stop codon	1	1
*Td-α-B1*	949	Intact full-length		
*Td-α-B2*	996	Intact full-length		
*Td-α-B3*	1023	Stop codon	1	1
*Td-α-B4*	969	Intact full-length		
*Td-α-B5*	927	Stop codon	1	1
*Td-α-B6*	918	Intact full-length		
*Td-α-B7*	765	TE insertion and stop codon	3	3
*Td-α-B8*	852	TE insertion and stop codon	3	3
*Td-α-B9*	813	Deletion at 5′ end and stop codon	3	3
*Td-α-B10*	1104	TE insertions and stop codon	1	0
*Td-α-B11*	231	Deletion at 5′ end and stop codon	1	0
*Td-α-B12*	669	Deletion at 5′ end and stop codon	1	1
*Td-α-B13*	606	Stop codon	2	0
*Td-α-B14*	146	Deletions at both 5′ and 3′ ends		
*Td-α-B15*	885	Intact full-length		
*Td-α-B16*	237	Deletion at 5′ end and stop codon	1	0

Note: *for pseudogeenes containing TE insertions, the length was calculated after the TE sequence was removed

The high number of α-gliadin pseudogenes correlates with a high frequency of mutation events. In most cases, multiple mutation events were detected in the pseudogenes (Table 1). In these scenarios, the first mutation that resulted in pseudogenization could not be determined. However, in six cases, a single point mutation caused an in-frame stop codon in the coding region. In five out of six cases, the point mutation occurred in the regions of the α-gliadin genes containing strings of glutamine codons. Glutamine residues (Q) are encoded by two codons, CAA and CAG. When compared to the non-interrupted full open reading frame (full ORF) α-gliadin genes, we found that these stop codons (TAA or TAG) were the result of a C to T change in CAA and CAG codons. Considering the high glutamine content in α-gliadins, it is likely that C to T transitions could be responsible for a majority of the premature stop codons in the pseudogenes. For instance, 40% of the codons in the active *Td-α-B4* gene encode glutamine. That means at least 40% of the residues could become stops with a single C to T base change. Many pseudogenes contained multiple in-frame stop codons. A total of 59 stop codons were identified in the α-gliadin genes in the wild emmer, 33 (56%) of which were in portions of the genes encoding poly Q regions or next to a Q residue.

### Structural organization of α-gliadin genes in the wild emmer genome

**Fig. 1:**
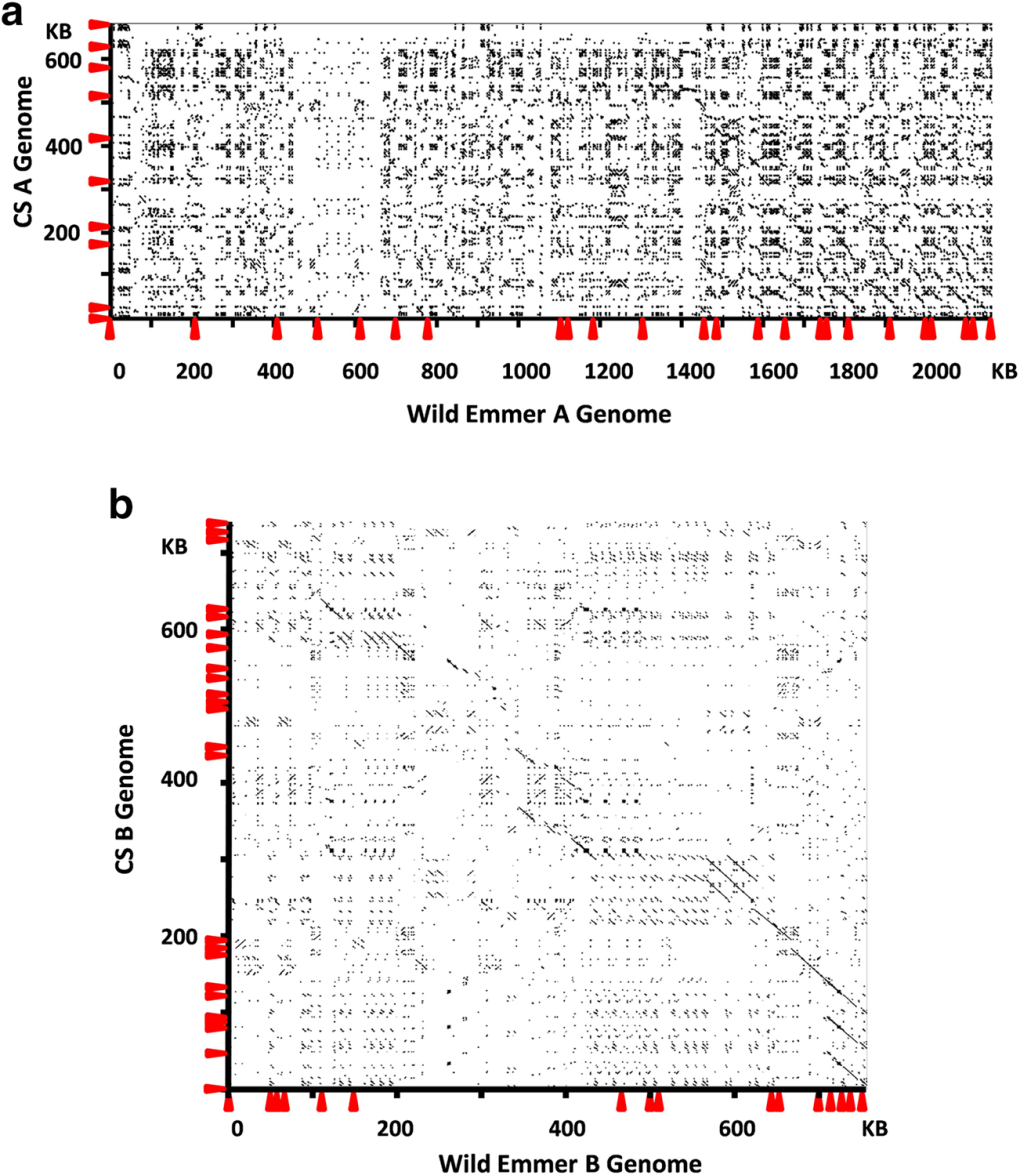
Pairwise comparison of α-gliadin genomic regions from two homologous genomes. Dotplot analyses between homologous regions from the A genomes (**a**) and B genomes (**b**) of wild emmer and hexaploid wheat Chinese Spring were performed using YASS program with default parameter setting (Noe and Kucherov 2005). The positions of α-gliadin genes are indicated with arrows along the axes

The genomic regions harboring *Gli-2* loci are complex due to the large number of duplications of α-gliadin genes. Therefore, we performed in-depth annotation of genes and repetitive DNA elements to delineate the genomic organization of α-gliadin genes in the A and B genomes of wild emmer. In the A genome, intergenic regions between two α-gliadin genes were primarily composed of transposable elements with nested insertion patterns (Supplementary Fig. S2). This greatly increased the size of the intergenic region and lowered the gene density. The estimated gene density was one α-gliadin gene per 107 kb based on a 2.5-Mb distance between the first (*Td-α-A1*) and last (*Td-α-A24*) α-gliadin gene. In comparison, the gene density was one gene every 70 kb in the homologous A genome in Chinese Spring based on a 700-kb region with a total number of 10 α-gliadin genes (Huo et al. 2018b). Therefore, it appears that both higher number of TE insertions and sequence duplications have greatly expanded the *Gli-2* locus region in the wild emmer A genome as compared to the region in CS. When the repetitive DNA elements in the intergenic region were examined, 10 out of the 23 α-gliadin gene intergenic regions in the wild emmer A genome had three shared elements (*Ada*, *Manor*, and *Sabrina*) (Supplementary Fig. S2), suggesting that most gene duplications occurred after the presence of these three elements. Insertions of other TEs likely occurred differentially in different intergenic regions. In the Chinese Spring A genome, the base structure also contained one of each *Ada*, *Manor*, and *Sabrina* elements in seven out of the nine intergenic regions. However, the *Sabrina* element was inserted into the *Manor* element, suggesting that only *Ada* and *Manor* were shared between the two A genomes (Supplementary Fig. S2). *Ada* and *Manor* only accounted for a small portion of the intergenic regions. The insertions of other large number of TEs likely occurred after the separation of two A genomes, resulting in considerable sequence divergence. To further examine the relationship of the two homologous regions, a dotplot matrix analysis was performed (Fig. 1a). A diagonal line representing close sequence identities was not observed, supporting the notion that intergenic regions were considerably diverged in the *Gli-2* locus regions of the two A genomes.

The α-gliadin gene density was much higher in the B genome α-gliadin region than in the A genome region, one gene per 48 kb. This mainly resulted from the smaller number of TE insertions in the intergenic regions (Supplementary Fig. S3). Except for the intergenic regions between *Td-α-B6* and *Td-α-B7* and between *Td-α-B1* and *Td-α-B2*, most intergenic regions contained less than 3 TE insertions. In the B genome, we identified four α-gliadin genes that were disrupted by insertions of TEs into the coding sequences and three out of the four genes shared the same TE disruptions with α-gliadin genes from CS. In addition, the large set of nested TE insertions between *Td-α-B6* and *Td-α-B7* in wild emmer was also shared with the CS region between *Ta-α-B10* and *Ta-α-B11* although some TEs were differentially inserted in the two regions (Supplementary Fig. S3). The shared TEs were useful in identifying orthologous gene pairs and defining orthologous regions that are conserved between the two genomes. For example, *Td-α-B7* in wild emmer is likely orthologous to *Ta-α-B19* in Chinese Spring as they have shared TE insertions (Supplementary Fig. S3). Therefore, unlike the two A genomes, considerable portions of the two B genome regions were conserved, as demonstrated by the dotplot matrix analysis (Fig. 1b). However, genomic sequences that are not shared by the two B genomes can be also easily detected. Based on the regions defined by shared TEs, we can propose that the region containing *Ta-α-B11* to *Ta-α-B18* are only present in CS, likely due to differential gene duplications in Chinese Spring or deletions in the wild emmer.

### Phylogenetic analysis of α-gliadin genes

**Fig. 2:**
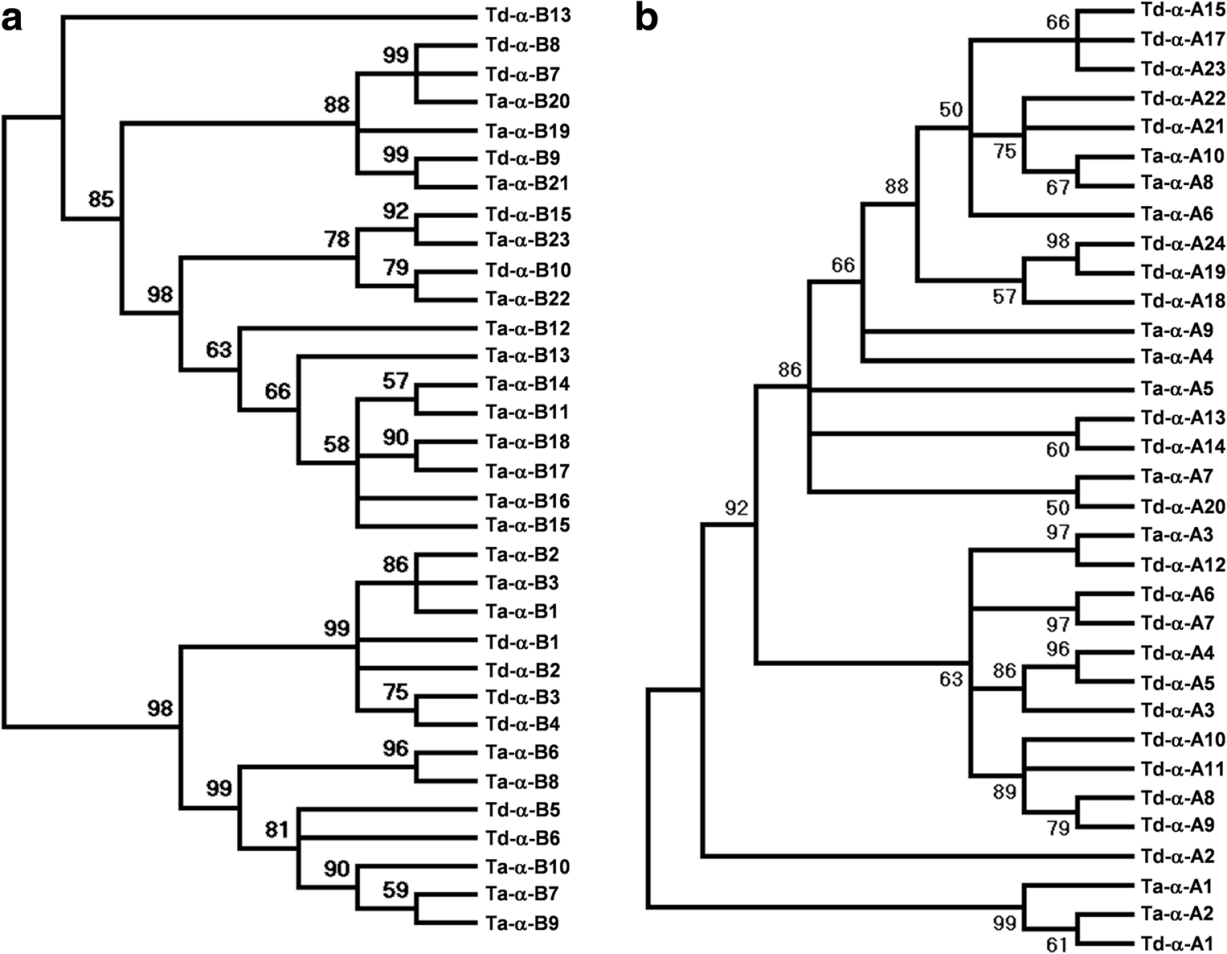
Phylogenetic trees of α-gliadin genes. Nucleotide sequences of α-gliadin gene sequences from the A genomes (**a**) and B genomes (**b**) of wild emmer and Chinese Spring were used for phylogenetic analysis. Phylogenetic trees were reconstructed with the neighbor-joining method. The bootstrap consensus tree inferred from 1000 replicates is taken to represent the evolutionary history of the α-gliadin genes analyzed. The percentages of replicate trees in which the associated genes clustered together in the bootstrap test (1000 replicates) are shown next to the branches

Understanding the evolutionary relationships of genes belonging to large gene families is often difficult because of the complexity in distinguishing orthologous (speciation) and paralogous (duplication) genes (Cannon and Young 2003; Panchy et al. 2016). Our previous study showed that amplification of most α-gliadin genes occurred independently in the wheat A, B, and D genomes, suggesting rapid and continuing evolution of the α-gliadin gene regions. To further understand α-gliadin gene duplication and evolution in a more recent time point, we reconstructed two phylogenetic trees, one for the α-gliadin genes from the A genomes of Chinese Spring and wild emmer wheat, and the other for the α-gliadin genes from the B genomes (Fig. 2a and Supplementary Fig. S4). We then compared the phylogenetic data with the structural organizations of α-gliadin regions to see if the tree results were supported. In the case of α-gliadin genes from the B genomes, *Ta-α-B22* from CS and *Td-α-B10* from wild emmer were grouped together, suggesting that they are orthologous genes. This result is supported by the notion that they both contained the same large nested TE insertions that likely occurred before the separation of the two A genomes (Supplementary Fig. S3). This is also true for *Ta-α-B19* and *Ta-α-B20* from CS and *Td-α-B7* and *Td-α-B8* from wild emmer as they were all disrupted by the same TE insertions before speciation. The phylogenetic tree also indicated other orthologous gene pairs including *Ta-α-B21* from CS and *Td-α-B9* from wild emmer, *Ta-α-B22* from CS and *Td-α-B10* from wild emmer, and *Ta-α-B23* from CS and *Td-α-B15* from wild emmer. In addition, the tree showed that all the α-gliadin genes of *Ta-α-B11* to *Ta-α-B18* from CS were grouped together. This agrees with our previous assumption based on shared TEs. All of these genes were located in the region between a shared TE insertion structure between *Ta-α-B10* and *Ta-α-B11* and the α-gliadin *Ta-α-B19* gene containing *Fatima* and *Inga* insertions, while the corresponding region was missing in wild emmer (Supplementary Fig. S3), supporting that they are paralogous genes derived from gene duplications in the A genome of hexaploid wheat.

In the phylogenetic tree for the A genome α-gliadin genes (Fig. 2b and Supplementary Fig. S5), three orthologous gene pairs can be easily identified between the two genomes. They were *Td-α-A1* and *Ta-α-A2*, *Td-α-A12* and *Ta-α-A3*, and *Td-α-A20* and *Ta-α-A7*. The other genes were likely differentially duplicated in the two A genomes. For example, *Td-α-A2* to *Td-α-A11* were only present in the wild emmer since no genes were present in the corresponding region in CS surrounded by two orthologous genes, *Ta-α-A2* and *Ta-α-A3*. In addition, genes after *Td-α-A13* in wild emmer and after *Ta-α-A4* in CS were also likely duplicated after the divergence of the two A genomes considering the differences in TE insertion patterns between α-gliadin genes. The notion that divergence of α-gliadin genomic regions in the two A genomes as shown by the dot plot matrix analysis (Fig. 1a) further supports the rapid evolution through gene duplications/deletions in the analyzed regions.

### Transcriptome analysis of α-gliadin gene expression

Individual members of large gene families may be differentially expressed but are difficult to characterize due to high sequence similarity. Mapping transcriptome reads with high stringencies can provide a much higher resolution in differentiating gene family members than other methods (Huo et al. 2018a). A total of 62 million reads from the developing grain of wild emmer 12 days post anthesis (12DPA) were extracted from a published dataset (Avni et al. 2017) and used to map to the complete set of annotated genes from wild emmer. Among 33 million reads that could be mapped to the complete gene set at a 99% identity stringency, 15.37% were from the α-gliadin genes, indicating a high abundance of α-gliadin transcripts in the developing grain tissue. 9.44% of these were α-gliadin genes from the A genome and 5.93% from the B genome.

**Fig. 3:**
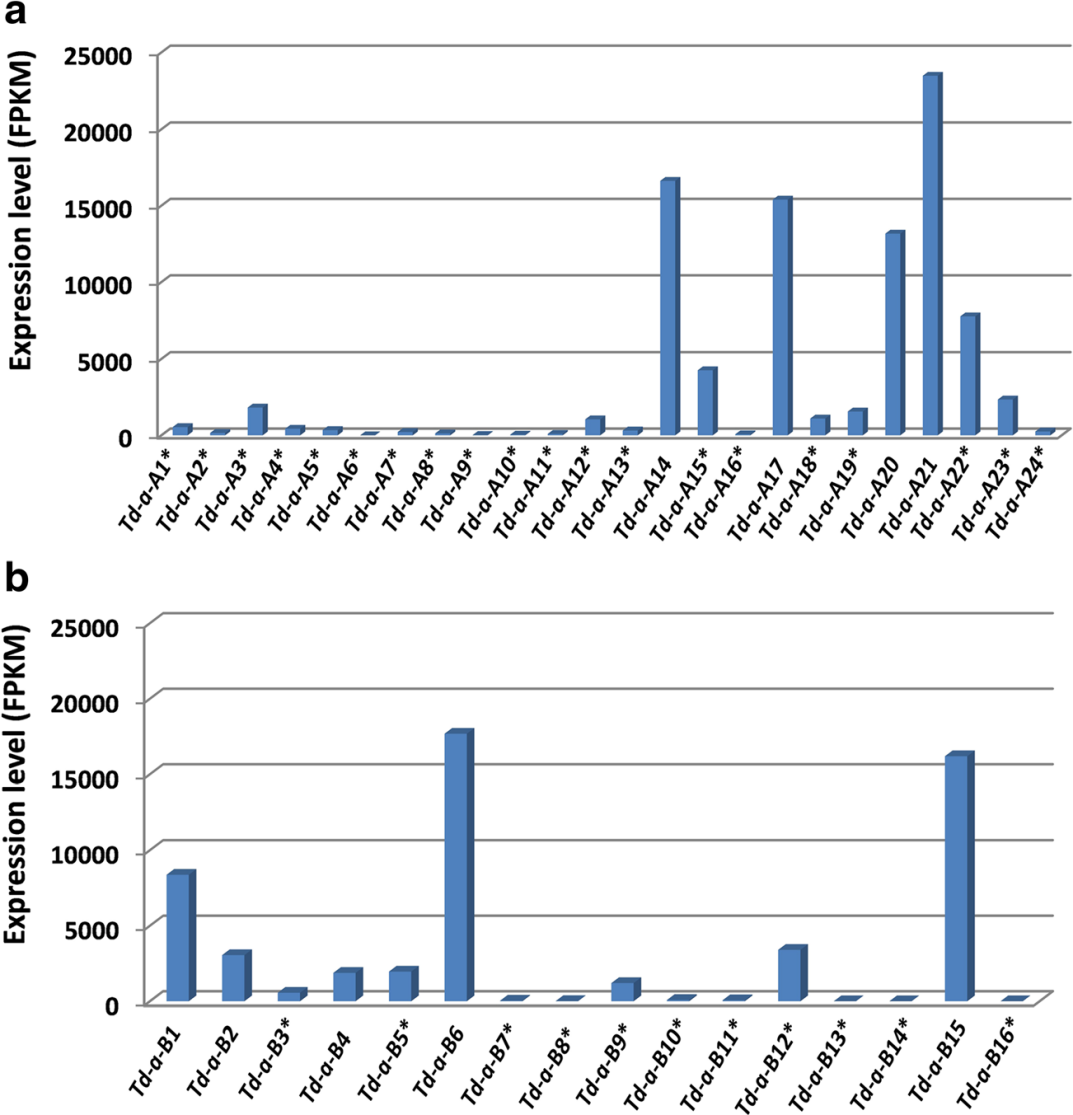
Expression profiles of α-gliadin genes in wild emmer. Expression profiles were generated using Illumina RNA-seq transcriptome datasets published previously from developing grain 12 days after anthesis (Avni et al. 2017). Pseudogenes are indicated with *

When the transcript level for individual genes was examined, full-length intact genes were highly expressed. All four intact genes in the A genome had FPKM (Fragments Per Kilobase Million) values over 10,000 (Fig. 3a). The expression level for pseudogenes was much lower, likely due to the instability of their transcripts regulated by nonsense-mediated mRNA decay mechanisms (Hug et al. 2016). However, *Td-α-A22* had a relatively high transcript level compared to other pseudogenes. Characterization of pseudogene-derived transcripts has been reported (Guo et al. 2014). mRNA from different pseudogenes might degrade at different rates. In the B genome, three out of five intact genes had FPKM values over 8000. However, the expression levels of the other two intact genes were relatively low, close to that of some of the pseudogenes (Fig. 3b). The promoter regions of all α-gliadin genes are very similar (Huo et al. 2017), so more detailed characterization will be needed to address the expression differences of the intact α-gliadin genes. One of the advantages in studying gene expression with transcriptome reads is that one can go back to the alignment data and check the result. This step was particularly useful for us to further validate the assembly of α-gliadin genes and pseudogenes manually.

### α-Gliadin proteins and CD epitopes

**Fig. 4:**
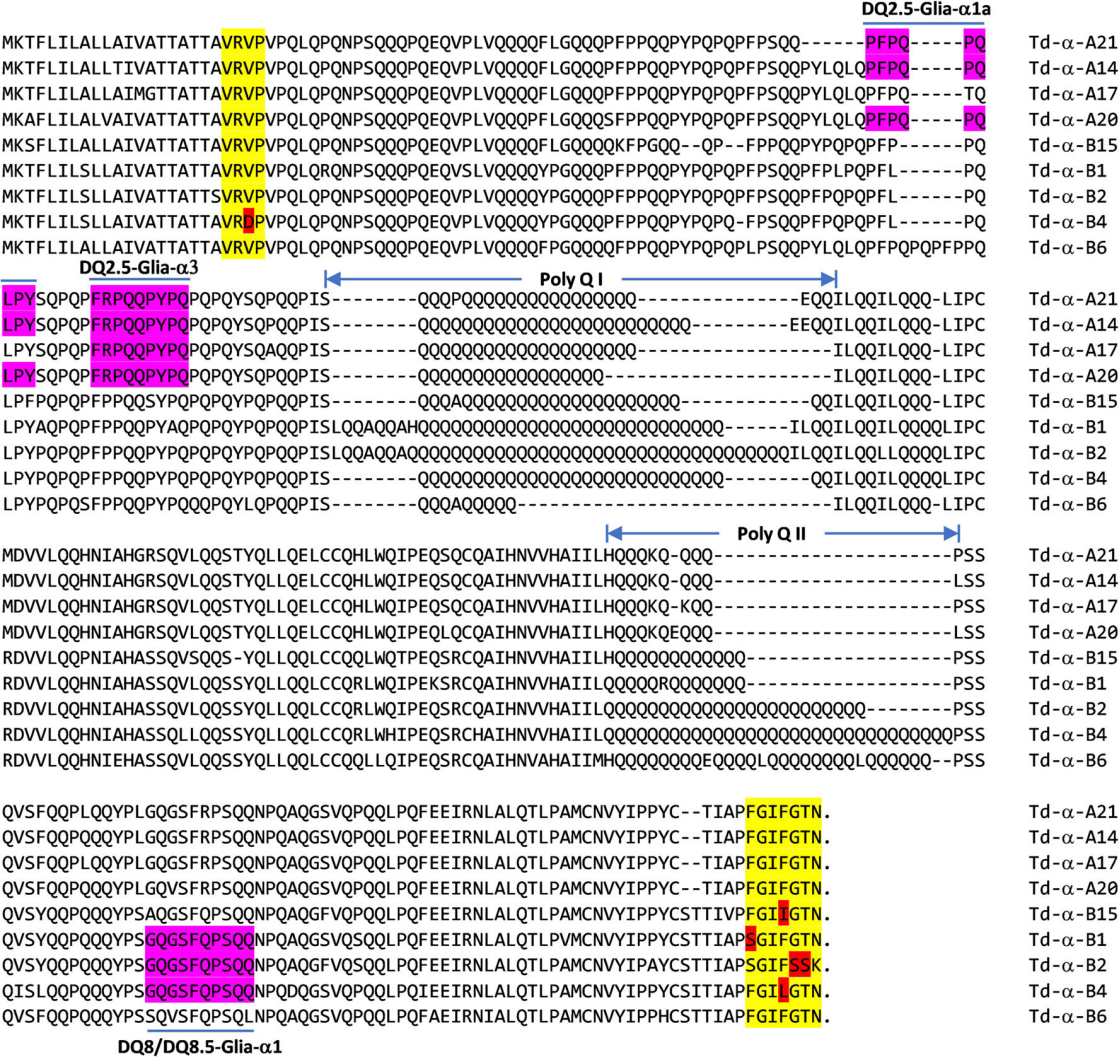
Sequence analysis of deduced α-gliadin proteins in wild emmer. Deduced protein sequences from nine full-length α-gliadin genes from wild emmer were aligned using ClusterW with manual editing to improve the alignment quality. The first four and last seven amino acid sequences of the mature α-gliadins are highlighted with yellow with amino acid substitutions indicated by red. The sequences of three CD epitopes are highlighted with magenta. Two poly Q regions are indicated

Based on sequence analysis and the transcriptome data, there are likely only four active α-gliadin genes in the A genome and five in the B genome that produce full-length proteins (Fig. 4). All of the encoded α-gliadins contained two poly Q domains separated by a non-repetitive region. Alignment of nine wild emmer α-gliadin sequences showed that they are generally conserved except in the poly Q regions where the number of glutamine residues varied greatly from 9 (Td-α-B6) to 41(Td-α-B2) in the Poly Q I region and from 6 (Td-α-A17) to 32 (Td-α-B4) in the Poly QII region. Variations in the number of Qs in the poly Q regions accounted for most of the differences in protein size among α-gliadins. We also noted that the A genome α-gliadins had very short poly Q II regions with only 6 to 7 Qs, while the B genome α-gliadins had longer and variable sizes (Fig. 4). In addition, the A genome α-gliadins had a conserved C-terminal region ending in GIFGTN. However, the last seven amino acid sequences were not the same in all the B genome α-gliadins.

The most significant T cell epitopes in celiac patients are PFPQPQLPY (DQ2.5-Glia-α1a), PYPQPQLPY (DQ2.5-Glia-α1b), PQPQLPYPQ (DQ2.5-Glia-α2), and FRPQQPYPQ (DQ2.5-Glia-α3) (Sollid et al. 2012). The most toxic 33-mer peptide is derived from the overlapping sequences of some of these epitopes. When we examined the wild emmer α-gliadins for T cell epitopes, it was found that all the A genome α-gliadins contained two T cell epitopes, DQ2.5-Glia-α1a and DQ2.5-Glia-α3, except Td-α-A17 in which P in DQ2.5-Glia-α1a was replaced by T, resulting from the mutation of a CCG to a ACG codon. Four out of five B genome α-gliadins contained only one minor epitope, DQ8/D8.5-Glia-α1. No epitopes were detected in Td-α-B6 and Td-α-B15 (Fig. 4).

## Discussion

Wheat gliadins are major seed storage proteins that contribute unique functional properties for food processing. However, gliadins are also the major cause of health-related issues associated with gluten-containing food products. Therefore, understanding the structure, evolution, and expression of gliadin genes in relation to gluten functionality will provide useful knowledge to facilitate the breeding of wheat varieties with improved end-use properties and reduced immunogenic potential. In this work, we performed a detailed analysis of genomic regions harboring *Gli-2* loci in wild emmer, the progenitor of both cultivated tetraploid pasta and hexaploid bread wheat, and compared the locus regions with the homologous regions of A and B genomes from the hexaploid wheat cv. Chinese Spring. This work provides new insights into the evolution of the complex wheat α-gliadin genes.

### Rapid evolution of genomic regions harboring α-gliadin genes

α-Gliadins are the youngest group of gluten proteins in wheat as they are only present in certain species in the Triticeae tribe. For instance, rye and barley, which diverged from wheat only about 7 to 10 MYA, do not contain α-gliadins but do possess genes encoding proteins similar to other groups of gluten proteins (Shewry and Halford 2002). α-Gliadin genes also evolve rapidly. For example, in the hexaploid wheat cv. Chinese Spring, there are 10, 26, and 11 α-gliadin genes in the A, B, and D subgenomes, respectively. Most of these α-gliadin genes evolved independently in the three homoeologous genomes that diverged ~ 2.3 to 2.4 MYA (Huo et al. 2018b). In this study, to further understand the evolution of α-gliadin genes, we compared α-gliadin genomic regions in the homologous A and B genomes from wild emmer and Chinese Spring. Given the estimates that tetraploid wheat formation occurred no more than 0.5 MYA (Chalupska et al. 2008), the comparison of the homologous regions of the A and B genomes from wild emmer and hexaploid wheat represents a much more recent time point. To our surprise, the two A genomes are quite different at the genomic structure level. First, the size of the genomic region harboring the α-gliadin gene family is 2.5 Mb in the A genome of wild emmer, while it is only 500 kb in the Chinese Spring A genome. Second, the wild emmer A genome contains a greater number of α-gliadin genes than Chinese Spring, 24 as compared to 10. Third, the intergenic regions contained differential TE insertions, although in some regions, shared TEs in the bottom of the nested sets could be identified. However, because of a large number of differential TE insertions, the α-gliadin regions in the two A genomes are generally not conserved as revealed by the dotplot analysis in Fig. 2a.

In comparison, the B genomes are more conserved as shared regions can be easily identified (Fig. 2b). We also noticed that multiple TE insertions in three α-gliadin genes in wild emmer (Fig. 2b) were also shared with the genes in the Chinese Spring B genome (Huo et al. 2018b). Our results suggest that the two A and two B genome α-gliadin regions might have different rates of sequence evolution. One explanation could be that TEs accelerate genomic structure changes. Studies have shown that the wheat genome contains a vast number of TEs. A high content of very similar TE sequences can cause frequent errors in recombination and lead to gene duplication and structural chromosome changes that could drive fast genome evolution (Luo et al. 2017). The gene density in the A genome regions is much higher than that in the B genome regions and correlates with high TE contents (Supplementary Fig. S2 and S3). Our results support the notions that variations in TE contents impact evolution at the local genomic scale.

### Evolution of α-gliadin genes and pseudogenes

Our analyses showed considerable variations in the α-gliadin gene copy numbers in the homologous *Gli-2* locus regions from tetraploid wild emmer and hexaploid wheat cv. Chinese Spring. Since α-gliadin genes are only mapped in *Gli-2* loci on the group 6 chromosomes, differential gene expansion and deletion within the locus regions likely played a major role in copy number variation. Phylogenetic trees facilitated identification of orthologous copies of α-gliadin genes that were present before the separation of the two homologous genomes. The identification of these orthologous gene pairs can often be further supported by the structure of targeted genomic regions, such as shared TE insertions (Fig. S2 and S3). Meanwhile, we also found that several groups of α-gliadin genes in the same clades were all from either wild emmer or Chinese Spring, suggesting that these are paralogous genes derived from local gene duplications that occurred after the separation of the two homologous genomes. However, we also cannot eliminate the possibility that the copy number variations result from differential deletions that occurred in one of the genomes. Nevertheless, our results support that rapid differential duplications/deletions occurring in the last ~ 0.5 MYA after the separation of the two homologous A and B genomes have resulted in considerable changes in the α-gliadin gene genomic regions.

Studies have shown that high proportions of α-gliadin genes are pseudogenes (Ozuna et al. 2015; van Herpen et al. 2006), ranging from ~ 12% in diploid wheat to 76% in tetraploid wheat (Ozuna et al. 2015). Although different mutational mechanisms that could result in pseudogenization of α-gliadin genes have been detected in this study and others (Huo et al. 2018b), we found that 28 out of 31 pseudogenes in wild emmer contained at least one internal in-frame stop codon (Table 1). α-Gliadins contain high percentages (30~50%) of Q residues encoded by CAA and CAG codons that can be mutated into stop codons when C is substituted by T. That means ~ 40% α-gliadin codons are potential stop codons, suggesting that the high frequency of pseudogenization of α-gliadin genes could result from single point mutations.

It appears that CAA and CAG codons for Q are not randomly distributed in the region, but tend to occur in homomeric runs of single codons, resulting in microsatellite sequences in α-gliadin gene sequences (Anderson and Greene 1997). Microsatellite repeat DNAs are known to be hypervariable as they are often involved in slippage-mispairing during DNA replication and could serve as hotspots for recombination (Anderson and Greene 1997). These events may have resulted in homogenization of the α-gliadin microsatellites with the possible effect of removing stop codons in pseudogenes or adding stop codons in active genes. Comparison of α-gliadin genes from hexaploid wheat D and *Ae. tauschii* D genomes revealed that pseudogenes in the ancestral genome could be reverted to become active genes in hexaploid wheat (Huo et al. 2017), supporting a role of pseudogenes in α-gliadin gene evolution.

It was also noticed that the frequency of α-gliadin pseudogenes varied greatly in wild emmer and hexaploid wheat genomes. In wild emmer, it is 83% (20/24) for the A genome and 69% (11/16) for the B genome. These frequencies are much higher than that in hexaploid wheat cv. Chinese Spring—20% (2/10) for the A genome and 58% (14/24) for the B genome (Huo et al. 2018b). It has been reported that diploid wheats have low frequencies of α-gliadin pseudogenes as compared to the polyploid wheats (Ozuna et al. 2015). This is understandable as the genetic redundancy of polyploid genomes could accelerate the accumulation of mutations, leading to pseudogenization of duplicated genes. Our data indicate that the tetraploid wild emmer has a higher frequency of α-gliadin pseudogenes than hexaploid wheat. This observation has also been reported in a previous study in which multiple accessions of hexaploid and tetraploid wheats were employed (Ozuna et al. 2015). Although the exact mechanisms are not known, hexaploid wheat varieties have experienced strong human selections for desirable traits including end-use qualities determined by gluten genes. Different types and high levels of α-gliadins could have impacts on both nutritional values and end-use quality of wheat grains, two valuable targeted traits in breeding selection, while the wild emmer used in this study is an undomesticated species. Genetic diversity analyses need to be performed in wild and domesticated wheat species to better understand the evolution of complex α-gliadin loci.

### α-Gliadin expression and immunogenic epitopes

In hexaploid bread wheat, α-gliadins are abundant, comprising 15–30% of the total seed storage proteins in the grain. Unfortunately, they are also the major protein group containing epitopes that trigger human immunogenic responses associated with celiac disease (Scherf et al. 2016). Therefore, several studies have analyzed α-gliadin components from diploid and tetraploid wheats and their wild relatives (Ozuna et al. 2015; Salentijn et al. 2013). Six distinct types of α-gliadins with strong differences in their frequencies in different wheat species were characterized and certain CD epitopes were associated with specific types of α-gliadins (Ozuna et al. 2015). In addition, both quantitative and qualitative differences in CD epitopes were found to be present in different tetraploid durum wheat accessions (Salentijn et al. 2013). In this study, we showed that in wild emmer, there are only nine full-length intact α-gliadin genes that encode functional proteins with different expression levels (Fig. 3). Among the nine predicted α-gliadin sequences, two of the four α-gliadins (Td-α-B6 and Td-α-B15) from the B genome are free of CD epitopes (Fig. 4). These two CD epitope-free α-gliadin genes are also among the most highly expressed genes based on transcriptome data analysis (Fig. 2). Our results support the importance of analyzing α-gliadin genomic regions from wild wheat species to identify useful resources that can be exploited in future breeding/selection of wheat varieties with reduced immunogenic potential.

## Electronic supplementary material

ESM 1 (PDF 161 kb)

ESM 2 (PDF 217 kb)

ESM 3 (PDF 155 kb)

ESM 4 (PDF 93 kb)

ESM 5 (PDF 110 kb)

ESM 6 (PDF 75 kb)

ESM 7 (PDF 101 kb)
