# Supplementary material for: Rapid evolution of α-gliadin gene family revealed by analyzing Gli-2 locus regions of wild emmer wheat
Source: Funct Integr Genomics. 2019 Jun 13;19(6):993–1005. doi: 10.1007/s10142-019-00686-z (PMC6797660; doi:10.1007/s10142-019-00686-z)
Supplement: Supplementary file 1 — (PDF 161 kb) [file 10142_2019_686_MOESM1_ESM.pdf]

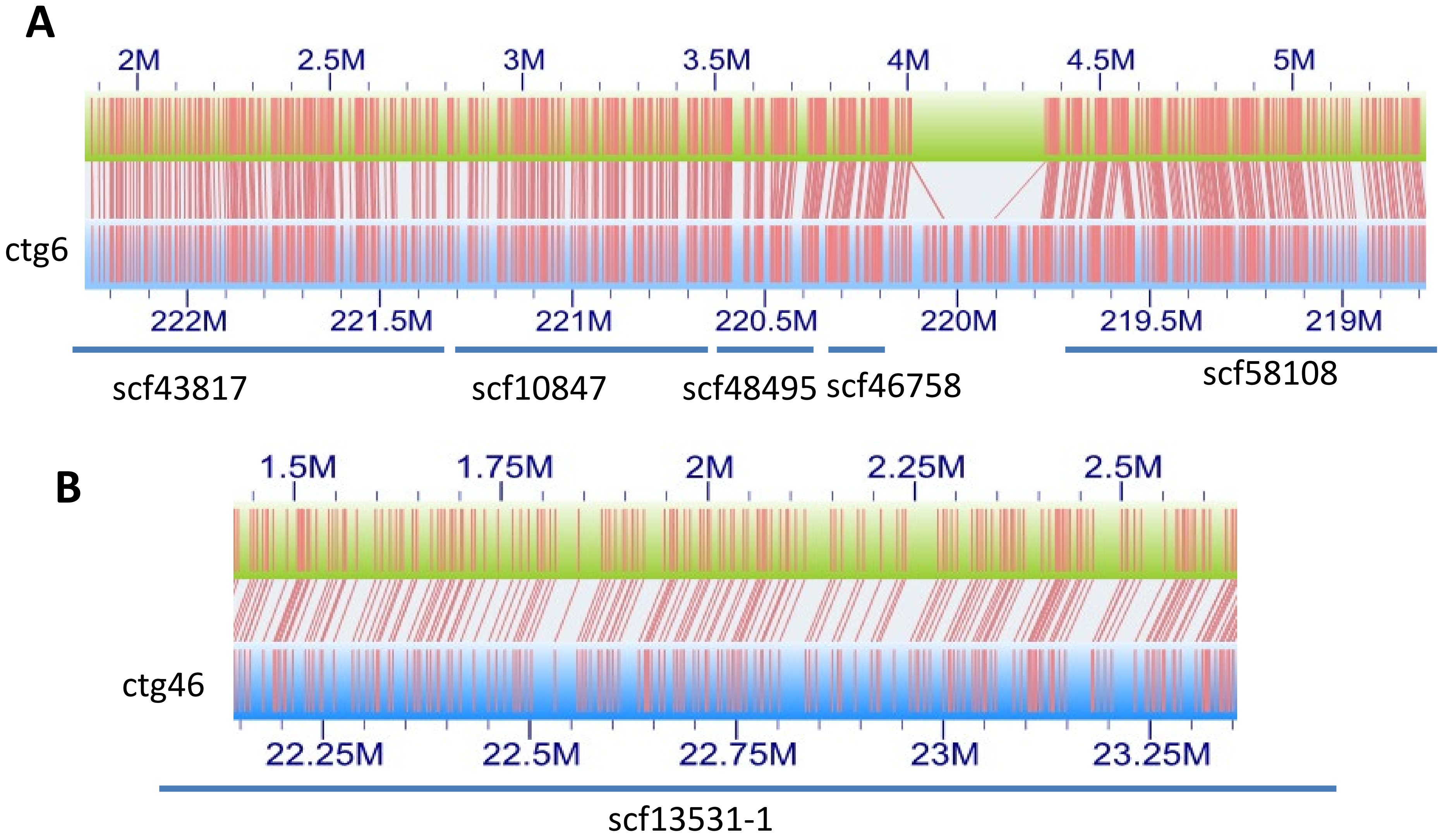

Figure S1. Reconstruction of α-gliadin genomic regions in wild emmer.
The extraction of wild emmer sequence scaffolds and alignment with BioNano maps is described in the Materials and Methods. Blue bars represent BioNano maps. Green bars are reconstructed sequences derived from difference scaffolds that align to the BioNano maps. Vertical lines represent agreements pf sequence motifs of endonuclease site (GCTTCTTC) between the consensus sequences and BioNano map contigs. Note: In the BioNano ctg6 contigs, there is one region with an estimated length of 500 kb that can not find any matches in the wild emmer genomic sequence scaffolds. Analysis showed that the gap was exactly located in a TE in a nested TE structure. It is possible that the contig contains a chimeric region.
