# Supplementary material for: Rapid evolution of α-gliadin gene family revealed by analyzing Gli-2 locus regions of wild emmer wheat
Source: Funct Integr Genomics. 2019 Jun 13;19(6):993–1005. doi: 10.1007/s10142-019-00686-z (PMC6797660; doi:10.1007/s10142-019-00686-z)
Supplement: Supplementary file 2 — (PDF 217 kb) [file 10142_2019_686_MOESM2_ESM.pdf]

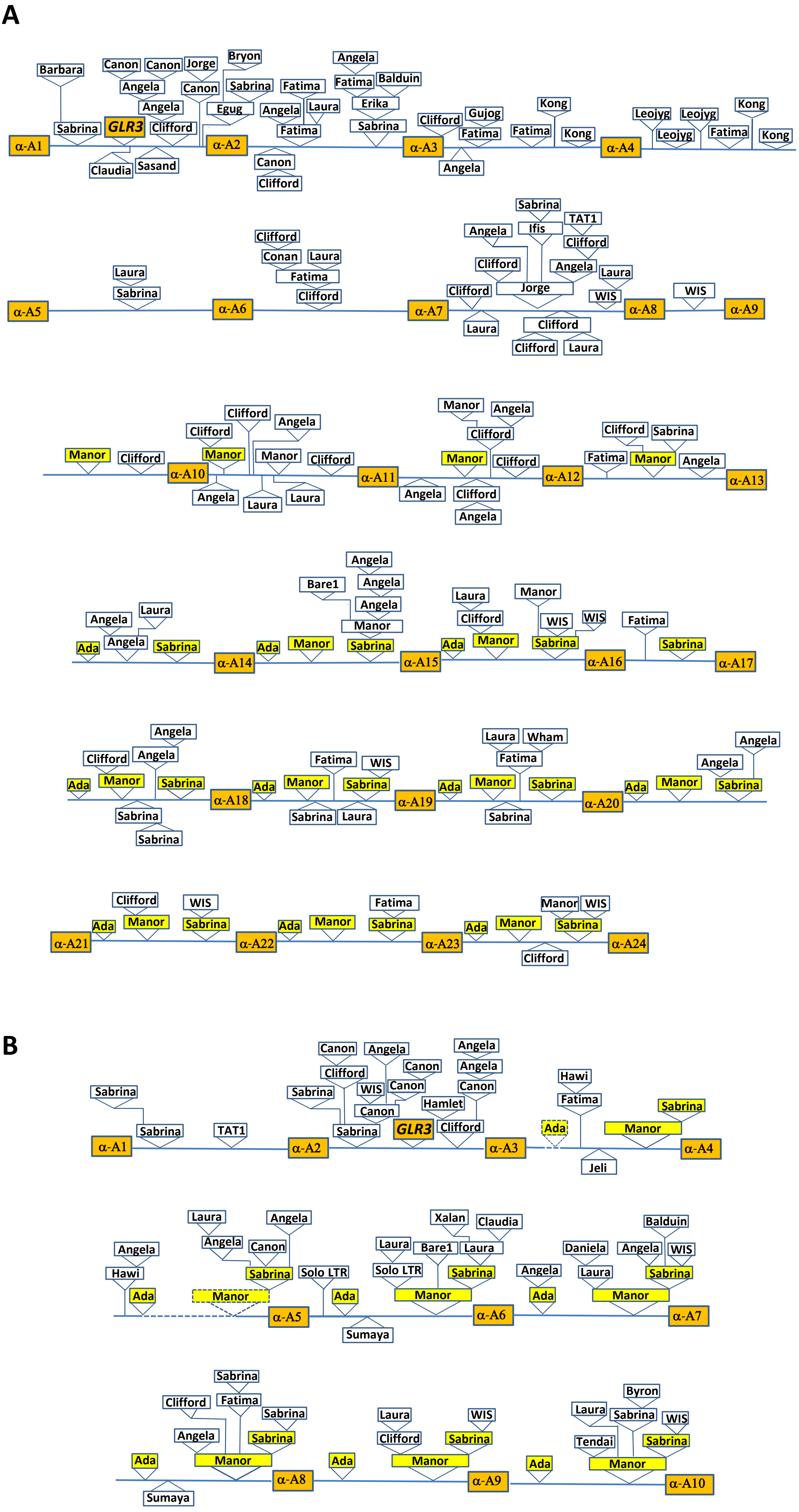

Figure S2. Organization of the α-gliadin locus region in the wild emmer and Chinese Spring A genomes.

The genomic organization of regions containing α-gliadin genes from wild emmer A genome (A) and Chinese Spring A genome (B) (Huo et al., 2018b) are displayed with α-gliadin genes represented by color-filled boxes. Retroelements are boxed with their names, and their insertion positions are indicated by triangles or solid vertical lines. Retroelement insertion sites that are likely shared in the intergenic regions or between two genomes are highlighted with yellow. Dashed lines indicate deleted sequences.
