# Supplementary material for: Rapid evolution of α-gliadin gene family revealed by analyzing Gli-2 locus regions of wild emmer wheat
Source: Funct Integr Genomics. 2019 Jun 13;19(6):993–1005. doi: 10.1007/s10142-019-00686-z (PMC6797660; doi:10.1007/s10142-019-00686-z)
Supplement: Supplementary file 4 — (PDF 93 kb) [file 10142_2019_686_MOESM4_ESM.pdf]

Supplementary Figure S4: Nucleotide sequence alignment of α-gliadin genes from the A genomes of hexaploid wheat Chinese Spring and wild emmer wheat

```
Ta-alpha-A4        ATGAAGACATTTCTCATCCTTGCCCTCCTTGCTATCGTGGCGACCACCGCCACAACTGCA
Ta-alpha-A5        ATGAAGACATTTCTCATCCTTGCCCTCCTTGCTATCGTGGCGACCACCGCCACAACTGCA
Td-alpha-A14       ATGAAGACGTTTCTCATCCTTGCCCTCCTTACTATCGTGGCGACCACCGCCACAACTGCA
Td-alpha-A20       ATGAAGGCATTTCTCATCCTTGCCCTCGTTGCTATCGTGGCGACCACCGCCACAACTGCA
Ta-alpha-A7        ATGAAGACCTTTCTCATCCTTGCCCTCCTTGCTATCGTGGCGACCACCGCCACAACCGCA
Ta-alpha-A9        ATGAAGACCTTTCTCATCCTTGCCCTCCTTGCTATCGTGGCGACCACCGCCACAACTGCA
Ta-alpha-A8        ATGAAGACCTTTCTCATCCTTGCCCTCCCTGCTATCGTGGCGACCACCGCCACAACTGCA
Ta-alpha-A10       ATGAAGACCTTTCTCATCCTTGCCCTCCTTGCTATCGTGGCGACCACCGCCACAACTGCA
Ta-alpha-A6        ATGAAGACCTTTCTCATCCTTGCCCTCCTTGCTATCGTGGCGACCACCGCCACAACTGCA
Td-alpha-A15       ATGAAGACCTTTCTCATCCTTGCCCTCCTTGCTATCGTGGCGACCACCGCCACAACTGCA
Td-alpha-A21       ATGAAGACCTTTCTCATCCTTGCCCTCCTTGCTATCGTGGCGACCACCGCCACAACTGCA
Td-alpha-A24       ATGAAGACCTTTCTCATCCTTGCCCTCCTTGCTATCGTGGCGACCACCGCCACAACTGCA
Td-alpha-A13       ATGAAGACATTTCTCATCCTTGCCCTCCTTGCTATCGTGGCGACCACCGCCACAACTGCA
Td-alpha-A17       ATGAAGACCTTTCTCATCCTTGCCCTCCTTGCTATCATGGGGACCACCGCCACAACTGCA
Td-alpha-A22       --------------------TGCCCTCCTTGCTATCGTGGCGACCACCGCCACAACTGCA
Td-alpha-A23       ATGAAGACCTTTCTCATCCTTGTCCTCCTTGCTATTGTGGCGACCACCGCCACAACTGCA
Td-alpha-A18       ATGAAGACCTTTCTCATCCTTGCCCTCCTTGCTATCGTGGCGACCACCGCCACAACTGCA
Td-alpha-A11       ATGAAGACCTTTCTCATCCTTTCCCTCCTTGCTATCGTGGAGACCACCGCCACAACTGCA
Td-alpha-A9        ATGAAGACCTTTCTCATCCTTTCCCTCCTTGCTATCGTGGAGACCACCGCCACAACTGCA
Td-alpha-A10       ATGAAGACCTTTCTCATCCTTTCCCTCCTTGCTATCGTGGAGACCACCGCCACAACTGCA
Td-alpha-A8        ATGAAGACCTTTCTCCTCCTTTCCCTCATTGCTATCGTGGAGACCACCACCACAACTGCA
Td-alpha-A12       ATGAAGACCTTTCTCATCCTTGCCCTCCTTGCTATCGTGGCGACCACCGCCACAACTGCA
Ta-alpha-A3        ATGAAGACCTTTCTCATCCTTTCCCTCCTTGCTATCGTGGCAACCACCGCCACAACTGCA
Td-alpha-A4        ATGAAGACCTTTCTCATTCTTGCACTCCTTGCTATCGTGGAGACCACCGCCGCAACTGCA
Td-alpha-A5        ATGAAGACCTTTCTCATTCTGGCACTCCTTGCTATCGTGGAGACCACCGCCGCAACTGTA
Td-alpha-A2        ATGAAGACCTTTCTCATCCTTGCCCTCCTTGCTATCGTGGCAACCACCGCCACAACTGTA
Td-alpha-A3        ATGAAGACCTTTCTCATCCTTGCCCTCCTTGCTATCGTGGCGACCACCGCCGCAACTTCA
Ta-alpha-A1        ATGAAGACTTTTCTCATCCTTGCCCTCCTTGCTATTGTGGCGACCACTGCCACAACTGCA
Ta-alpha-A2        ATGAAGACATTTCTCATCATTTCCCTCCTTGCTATCGTGGCGACCACCGCCACAACTGCA
Td-alpha-A1        ATGAAGACACTTCTCATCCTTTCCCTCATTGCTATCGTGGCGACCACTGCCACAACTGCA
Td-alpha-A19       ATGAAGACCTTTCTCATCCTTGCCCTCCTTGCTATCGTGGCGACCACCGCCACAATTGCA
Td-alpha-A7        AT----------------------------------------------------------
Td-alpha-A6        ------------------------------------------------------------
Td-alpha-A16       ATGAAGACCTTTCTCATCCTTGCCCTCCTTGCTATCGTGGCGACCACCGCCACAACTGCA

Ta-alpha-A4        GTTAGAGTTCCAGTGCCACAATTGCAGCCACAAAATCCATCTCAGCAACAGTCACAAGAG
Ta-alpha-A5        GTTAGAGTTCCAGTGCCACAATTGCAGCCACAAAATCCATCTCAGCAACAGCCACAAGAG
Td-alpha-A14       GTTAGAGTTCCAGTGCCACAATTGCAGCCACAAAATCCATCTCAGCAACAGCCACAAGAG
Td-alpha-A20       GTTAGAGTTCCAGTGCCACAATTGCAGCCACAAAATCCATCTCAGCAACAGCCACAAGAG
Ta-alpha-A7        GTTAGAGTTCCAGTGCCACAATTGCAGCCACAAAATCCATCTCAGCAACAGCCACAAGAG
Ta-alpha-A9        GTTAGAGTTCCAGTGCCACAATTGCAGCCACAAAATCCATCTCAGCAACAGCCACAGGAG
Ta-alpha-A8        GTTAGAGTTCCAGTGCCACAATTGCAGCCACAAAATCCATCTCAGCAACAGCCACAAGAG
Ta-alpha-A10       GTTAGAGTTCCAGTGCCACAATTGCAGCCACAAAATCCATCTCAGCAACAGCCACAAGAG
Ta-alpha-A6        GTTAGAGTTCCAGTGCCACAATTGCAGCCACAACATCCATCTCAGCAACAGCCACAAGAG
Td-alpha-A15       GTTAGAGTTCCAGTGCCACAATTGCAGCCACAAAATCCATCTCAGCAACAGCCACAAGAG
Td-alpha-A21       GTTAGAGTTCCAGTGCCACAATTGCAGCCACAAAATCCATCTCAGCAACAGCCACAAGAG
Td-alpha-A24       GTTAGAGTTCTAGTGCCACAATTGCAGCCATAAAATCCATCTCAGCAACAGCCACAAGAG
Td-alpha-A13       GTTAGAGTTCCAGTGCCACAATTGCAGCCACAAAATCCATCTCAGCAACAGCCACAAGAG
Td-alpha-A17       GTTAGAGTTCCAGTGCCACAATTGCAGCCACAAAATCCATCTCAGCAACAGCCACAAGAG
Td-alpha-A22       GTTAGAGTTCCAGTGCCACAATTGCAGCCACAAAATCCATCTCAGCAACACCCACAAGAG
Td-alpha-A23       GTTAGATTTCCAGTGCCACAATTGCAGCCACAAAATCCATCTCAGCAACAGCCACAAGAG
Td-alpha-A18       GTTAGAGTTACAGTGCCACAATTGCAGCCATAAAATCCATCTCAGCAACAGCCACAAGAG
```

```
Td-alpha-A11      GTTAGAGTTCCAGTGCCACAATTGCAGCCACAAAATCCATCTCAGCAACAGCCACAAGAG
Td-alpha-A9       GTTAGAGTTCCAGTGCCACAATTGCAGCCACAAAATCCATCTCAGCAACAGCCACAAGAG
Td-alpha-A10      GTTAGAGTTCCAGTGCCACAATTGCAGCCACAAAATCCATCTCAGCAACAGCCACAAGAG
Td-alpha-A8       GTTAGAGTTCCAGTGCCACAATTGCAGCCACAAAATCCATCTCAGCAACAGCCACAAGAG
Td-alpha-A12      TTTAGAATTCCAGTGCCACAATTGCAGCCACAAAATCCATCTTAGCAACAGCCACAATAG
Ta-alpha-A3       TTTAGAATTCTAGTGCCACAATTGCAGCCACAAAATCCATCTTAGCAACAACCACAAGAG
Td-alpha-A4       GTTAGAGTTCCAGTGCCACAATTGCAGCCACAAAATCCATCTCAGCAACAACCACAAGAG
Td-alpha-A5       GTTAGAGTTCCAGTGCCACAATTGCAGCCACAAAATCCATCTCAGCAACAACCACAAGAG
Td-alpha-A2       GTTAGAGTTCCAGTGCCACAACCGTAGCCACAAAATCGACCTCAGCCACAGCCACAAGGG
Td-alpha-A3       GTTAGAGTTCCAGTGCCACAATTGCAGCCACAAAATCCATCTCAGCAACAGCCACAAGAG
Ta-alpha-A1       GTTAGAGTTCCAGTGCCACAATTGCAGCCGCAAAATCCATCTCTGCAGCAACCACAAGAG
Ta-alpha-A2       GTTAGAGTTCCAGTGCCACAATTGCAGCCGCAAAATCCATCTCTGCAACAACCACAAGAG
Td-alpha-A1       GTTAGAGTTCCAGTGCCACAATTGCAGCTGCAAAATCCATCTATGCAACAACCACAAGAG
Td-alpha-A19      GTTAGAGTTCCAGTGCCACAATTGCAGCCATAAAATCCATCTCAGCAACATCCACAAGAG
Td-alpha-A7       -------------------ATTCGCAGCCGCAACTACCATATTCGT--------------
Td-alpha-A6       ------------------------------------------CAACAACAACAACAACAA
Td-alpha-A16      GTTAGAGTTCCAGTGCCACAATTGCAGCCACAAAATCCATCTCAGCAACAGCCACAAGAG

Ta-alpha-A4       CAAGTTCCATTGGTACAACAACAACAATTTCTAGGGCAGCAACAACCATTTCCACCACAA
Ta-alpha-A5       CAAGTTCCATTGGTACAACAACAACAATTTCTAGGGCAGCAACAACCATTTCCACCACAA
Td-alpha-A14      CAAGTTCCATTGGTACAACAACAACAATTTCTAGGGCAGCAACAACCATTTCCACCACAA
Td-alpha-A20      CAAGTTCCATTGGTACAACAACAACCATTTCTAGGGCAGCAACAATCATTTCCACCACAA
Ta-alpha-A7       CAAGTTCCATTGGTACAACAACAACAATTTCTAGGGCAGCAACAAACATTTCCACCACAA
Ta-alpha-A9       CAAGTTCCAGTGGTACAACAACAACAATTTCTAGGGCAGCAACAACCATTTCCACCACAA
Ta-alpha-A8       CAAGTTCCATTGGTACAACAACAACAATTTCTAGGGCAGCAACAACCATTTCCACCACAA
Ta-alpha-A10      CAAGTTCCATTGGTACAACAACAACAATTTCTAGGGCAGCAACAACCATTTCCACCACAA
Ta-alpha-A6       CAAGTTCCATTGGTACAACAACAACAATTTCTAGGGCAGCAACAACCATTTCCACCACAA
Td-alpha-A15      CAAGTTCCATTGGTACAACAACAACAATTTCTAGGGTAGCCACAACCATTTCCACCACAA
Td-alpha-A21      CAAGTTCCATTGGTACAACAACAACAATTTCTAGGGCAGCAACAACCATTTCCACCACAA
Td-alpha-A24      CAAGTTCCATTGGTACAACAACAACAATTTCTAGGGCAGCAACAACCATTTCCACCACAA
Td-alpha-A13      CAAGTTCCATTGGTACAACAACAACAATTTCTAGGGCAGCAACAACCATTTCCACCACAA
Td-alpha-A17      CAAGTTCCATTGGTACAACAACAACAATTTCTAGGGCAGCAACAACCATTTCCACCACAA
Td-alpha-A22      CAAGTTCCATTGGTACAACAACAACAATTTCTTGGGCAGCAACAACCATTTCCACCACAA
Td-alpha-A23      CAAGTTCCATTGGTACAACAACAACAATTTCTAGGGCAGCAACAACCATTTCCACCACAA
Td-alpha-A18      CAAGTTCCATTGGTACAACAACAACAATTTCTAGGGCAGTAACAACCATTTCCACCACAA
Td-alpha-A11      CAAGTTCCATTGGTACAACAACAAAAATTTCTAGGGCAGCAACAACAATTTCCACCACAA
Td-alpha-A9       CAAGTTCCATTGGTATAACAACAAAAATTTCTAGGGCAGCAACAACAATTTCCACCACAA
Td-alpha-A10      CAAGTTCCATTGGTACAACAACAAAAATTTCTAGGGCAGCAACAACAATTTCCACCACAA
Td-alpha-A8       CAAGTTCCATTGGTACAACAACAATAATTTCTAGGGCAGCAACAACAATTTCCACCACAA
Td-alpha-A12      CAAGTTCCATTGGTACAACAACAACAATTTCTAGGGCAGCAACAACCATTTCCACCACAA
Ta-alpha-A3       CAAGTTCCATTGGTACAACAACAGCAATTTCTAGGGCAGCAACAACCATTTCCACCACAA
Td-alpha-A4       CAAGTTCCATTGGTACAACAACAACAATTTCTAGGGAAGCAACAACAATTTCCAACAGAA
Td-alpha-A5       CAAGTTCCATTGGTACAACAACAACAATTTCTAGGGCAGCAACAACAATTTCCAACAGAA
Td-alpha-A2       CAAGTTCCATTGGTACAACAACAACAATTTCCAGGGCAGCAACAACCATTTCCACCACAA
Td-alpha-A3       CAAGTTCCATTGGTACAACAACAACAATTTCTAGGGCAGCAACAACAATTTCCACCACAA
Ta-alpha-A1       CAAGTTCCATTGGTGCAACAACAACAATTTCCAGGGCAGCAACAAACATTTCCACCACAA
Ta-alpha-A2       CAAGTTCCATTGGTGCAACAACAACAATTTCCAGGGCAGCAACAAACATTTCCACCACAA
Td-alpha-A1       CAAGTTCCATTGGTGCAACAACAACAATTTCTAGGGCAGCAACAAACATTTCCACCACAA
Td-alpha-A19      CAAGTTCCATTGGTACAACAACAACAATTTCTAGGGCAGCAACAACCATTTCCACCACAA
Td-alpha-A7       -------------------------------------AGCAGCAACCATTTCGACCACAA
Td-alpha-A6       CAA------------CAACAACAACAA---------CAACAACAACAA------------
Td-alpha-A16      CAAGTTCCATTGGTACAACAACAACAATTTCTAGGGCAGCAACAACCATTTCCACCACAA

Ta-alpha-A4       CAACCATATCCACAGCCGCAACCATTTCCATCACAACAACCATATCTGCAGCTGCAACCA
Ta-alpha-A5       CAACCATATCCACAGCCGCAACCATTTCCATCACAACAACCATATCTGCAGCTGCAACCA
Td-alpha-A14      CAACCATATCCACAGCCACAACCATTTCCATCACAACAACCATATCTGCAGCTGCAACCA
Td-alpha-A20      CAACCATATCCACAGCCGCAGCCATTTCCATCACAACAACCATATCTGCAGCTGCAACCA
Ta-alpha-A7       CAACCATATCCACAGCTGCAACCATTTCCATCACAACAACCATATCTACAGCTGCAACCA
Ta-alpha-A9       CAACCATATCCACAGCCGCAACCATTTCCATCACAACAACCATATCTGCAGCTGCAACCA
```

```
Ta-alpha-A8       CAACCATATCCACAGCCGCAACCATTTCCATCACAACAACCATATCTGCAGCTGCAACCA
Ta-alpha-A10      CAACCATATCCACAGCCGCAACCATTTCCATCACAACAACCATATCTGCAGCTGCAACCA
Ta-alpha-A6       CAACCATATCCACAGCCGCAACCATTTCCATCACAACAACCATATCTGCAACTACAACCA
Td-alpha-A15      CAACCATATCCACAGCCGCAACCATTTCCATCACAACAACCATATCTGCAACTGCAACCA
Td-alpha-A21      CAACCATATCCACAGCCGCAACCATTTCCATCACAACAACCAT-----------------
Td-alpha-A24      CAACCATATCCACAGGCGCAACCATTTCCATCACAACAACCATATCTGCAGCTGCAACCA
Td-alpha-A13      CAACCATATCCAGAGCCGCAACCATTTCCATCACAACAACCATATCTGCAGCTGCAACCA
Td-alpha-A17      CAACCATATCCACAGCCGCAACCATTTCCATCACAACAACCATATCTGCAGCTGCAACCA
Td-alpha-A22      CAACCATATCCACAGCCGCAACCATTTCCATCACAACAACCATATCTGCAGCTGCAACCA
Td-alpha-A23      CAACCATATCCACAGCCGCAACCATTTCCATCACAACTACCATATCTGCAGCTGCAACCA
Td-alpha-A18      CAACCATATCCACAGCCGCAACCATTTCCATCACAACAACCATATCTGCAGCTGCAACCA
Td-alpha-A11      CAACCAGATCCACAGCCGCAACCATTTCCATCACAACAACCATATCTGCAGTTGCAACCA
Td-alpha-A9       CAACCAGATCCACAGCCGCAACCATTTCCATCACAACAACCATATCTGCAGCTGCAACCA
Td-alpha-A10      CAACCAGATCCACAGCCGCAACCATTTCCATCACAACAACCATATCTACAGCTGCAACCA
Td-alpha-A8       CAACCATATCCACAGCCGCAACCATTTCCATCACAACAACCATATCTGCAGTTGCAACCA
Td-alpha-A12      CAACCATATCCACAGCCGCAACCATTTCCATCACAACAACCATATCTGCAGCTGCAACCA
Ta-alpha-A3       CAACCATATCCACAGCCGCAACCATTTCCATCACAACAACCATATCTGCAGCTGCAACCA
Td-alpha-A4       CAACCATATCCGCAGCCGCAACCATTTCCATCACAACAACCATATCTGCAGCTGCAACCA
Td-alpha-A5       CAACCATATCCGCAGCCGCAACCATTTCCATCACAACAACCATATCTGTAGCTGCAACCA
Td-alpha-A2       CAGCCATATCCGCAGCCGCAACCATTTCCATCACAACAACCATATCTGCAACTGCAACCA
Td-alpha-A3       CAACCATATCCGCAGCCGCAACCATTTCCATCACAACAACCATATCTGCAGCTGCAACCA
Ta-alpha-A1       CAGCCATATCCGCAGCCGCAACCATTTCCAGCACAACAACCATATCCGCAGCCGCAACTA
Ta-alpha-A2       CAACCATATCCGCAGCCGCAACCATTTCCAGGACAACAACCATATCCGCAGCCGCAACTA
Td-alpha-A1       CAGCCATATCCACAGCCGCAACCATTTCCAGCACAACAACCATATCCGCAACCGCAACCA
Td-alpha-A19      CAACCATATCCACAGCCGCAACCATTTCCATCACAACAACCATATCTGCAGCTGCAACCA
Td-alpha-A7       CAACCATATCCACAACCGCAACCACATTATTCGCAACCACAA------------CAACCA
Td-alpha-A6       ------------------------------------------------------------
Td-alpha-A16      CAACCATATCCATAGGCGCAACCATTTCCATCACAACAATCATATCTGTAGCTGCAACCA

Ta-alpha-A4       TTTCCGCAGCCGCAACTAC--------------C-ATATTCGCAGCCACAACCATTTCGA
Ta-alpha-A5       TTTCCGCAGCCGCAACTAC--------------C-ATATTCGCAGCCACAACCATTTCGA
Td-alpha-A14      TTTCCACAGCCGCAACTAC--------------C-ATATTCGCAACCACAACCATTTCGA
Td-alpha-A20      TTTCCGCAGCCGCAACTAC--------------C-ATATTCGCAGCCACAACCATTTCGA
Ta-alpha-A7       TTTCCGCAGCCGCAACTAC--------------C-ATATTCGCAGCCACAACCATTTCGA
Ta-alpha-A9       TTTCCGCAGCCGCAACTAC--------------C-ATATTCGCAGCCACAACCATTTCAA
Ta-alpha-A8       TTTCCGCAGCCGCAACTAC--------------C-ATATTCGCAGCCACAACCATTTCGA
Ta-alpha-A10      TTTCCGCAGCCGCAACTAC--------------C-ATATTCGCAGCCACAACCATTTCGA
Ta-alpha-A6       TTTCCGCAGCCGCAACTAC--------------C-ATATTCGCAGCCACAACCATTTCGA
Td-alpha-A15      TTTCCGCAGCCGCAACTAC--------------C-ATATTCACAGCCACAACCATTTCGA
Td-alpha-A21      -TTCCGCAGCCGCAACTAC--------------C-ATATTCGCAGCCACAACCATTTCGA
Td-alpha-A24      TTTCCGCAGCCGCAACTAC--------------C-ATATTCGCAGCCACAACCATTTCGA
Td-alpha-A13      TTTCCACAGCCGCAACTAC--------------C-ATATTCGCAACCACAACCATTTCGA
Td-alpha-A17      TTTCCGCAGACGCAACTAC--------------C-ATATTCACAGCCACAACCATTTCGA
Td-alpha-A22      TTTCCGCAGCCGCAACTAC--------------C-ATATTTGCAGCCACAACCATTTCGA
Td-alpha-A23      TTTCCGCAGCCGCAACTAC--------------C-ATATTCACAGCCACAACCATTTCGA
Td-alpha-A18      TTTCCGCAGCCGGAACTAC--------------C-ATATTCGCAGCCACAACCATTTCGA
Td-alpha-A11      TTTCCGCAGCCGCAACTAC--------------C-ATATTCTCAGCCGCAACCATTTCGA
Td-alpha-A9       TTTCCGCAGCCGCAACTAC--------------C-ATATTCGCAGCCGCAACCATTTCGA
Td-alpha-A10      TTTCCGCAGCCGCAACTAC--------------C-ATATTCGCAGCCGCAACCATTTCGA
Td-alpha-A8       TTTCCGCAGCCGCAACTAC--------------C-ATATTCGCAACCGTAACCATTTCGA
Td-alpha-A12      TTTCCGCAGCCGCAACTAC--------------C-ATATTCGCAGCCGCAACCATTTCGA
Ta-alpha-A3       TTTCCGCAGCCGCAACCAC--------------C-ATATTCGCAGCCGCAACCATTTCGA
Td-alpha-A4       TTTTCGCAGCCGCAACTAC--------------CCATATTCGCAGCCGCAACCATTTCGA
Td-alpha-A5       TTTTCGCAGCCGCAACTAC--------------CCATATTCTCAGCCGCAGCCATTTCGA
Td-alpha-A2       TTTCCACAACCGCAACCATTTCCGCCACAACTACCATATCCGCAGCCACCACCATTTTCA
Td-alpha-A3       TTTTCGCAGCCGCAACTA---------------CCATATTCGCAGCCGCAACCATTTCGA
Ta-alpha-A1       TTTCCACAGCCGCAACCTTTTCCGCCACAACTTCCATATCCGCAGCCGCAACCATTTCCC
Ta-alpha-A2       TTTCCACAGCCGCAACCTTTTCCGCCACAACTTCCATATCCGCAGCCGCAACCATTTCCC
Td-alpha-A1       TTTCCACAGCCGCAACCTTTTCCGCCACAACTACCATATCCGCAGCCGCAACCATTTCCC
```

```
Td-alpha-A19      TTTCCGCAGCCGCAACTA---------------CCATATTCGCAGCCACAACCATTTCGA
Td-alpha-A7       GTTTCGCACCAGCAGCAA------------------------CAGCAACAACAACAACAA
Td-alpha-A6       ------------------------------------------------------------
Td-alpha-A16      TTTCCGCAGCTGCAACTA---------------CCATATTTGCAGCCACAACCATTTCGA

Ta-alpha-A4       CCACAACAACCATATC--CACAACCGCAACCACA------ATATTCGCAACCACAACATC
Ta-alpha-A5       CCACAACAACCATATC--CACAACCGCAACCACA------GTATTCGCAACTACAACAAC
Td-alpha-A14      CCACAACAACCATATC--CACAACCGCAACCACA------GTATTCGCAACCACAACAAC
Td-alpha-A20      CCACAACAACCATATC--CACAACCGCAACCACA------GTATTCGCAACCACAACAAC
Ta-alpha-A7       CCACAACAACCATATC--CACAACCGCAACCACA------GTATTCGCAACCACAACAAC
Ta-alpha-A9       CCACAACAACCATATC--CACAACCGCAACCACA------GTATTCGCAACCACAACAAC
Ta-alpha-A8       CCACAACAACCATATC--CACAACCGCAACCACA------GTATTCGCAACCACAACAAC
Ta-alpha-A10      CCACAACAACCATATC--CACAACCGCAACCACA------GTATTCGCAACCACAAGAAC
Ta-alpha-A6       CCACAACAACCATATC--CACAACCGCAACCACA------GTATTCGCAACCACAACAAC
Td-alpha-A15      CCACAACAACCATATC--CACAACCACAACCACA------GTATTCGCAACCACAACAAC
Td-alpha-A21      CCACAACAACCATATC--CACAACCGCAACCACA------GTATTCGCAACCACAACAAC
Td-alpha-A24      CCACAACAACCATATC--CACAACCGCAACCACA------GTATTCGCAACCACAACAAC
Td-alpha-A13      CCACAACAACCATATC--CACAACCGCAACCACA------GTATTCGCAACCACAACAAC
Td-alpha-A17      CCACAACAACCATATC--CACAACCACAACCACA------GTATTCGCAAGCACAACAAC
Td-alpha-A22      CCACAACAACCATATC--CACAACCGCAACCACA------GTATTCGCAACCACAACAAC
Td-alpha-A23      CCACAACAACCGTATC--CACAACCGCAACCACA------GTAGTCGCAACCACAACAAC
Td-alpha-A18      CCACAACAACCATATC--CACAACCGCAACCACAACCACAGTATTCGCAACCACAACAAC
Td-alpha-A11      CCACAACAACCATATC--CACAACCGCAACCACA------GTATTCGCAACCACAACAAC
Td-alpha-A9       CCACAACAACCATATC--CACAACCGCAACCACA------GTATTCGCAACCACAACAAC
Td-alpha-A10      CCACAACAACCATATC--TACAACCGCAACCACA------GTATTCGCAACCACAACAAC
Td-alpha-A8       CCACAACAACCATATC--CACAACCGTAACCACA------GTATTCGCAACCACAACAAC
Td-alpha-A12      CCACAACAACCATATC--CACAACCGCAACCATA------GTATTTGCAACCACAATAAC
Ta-alpha-A3       CCACAATAACCATATCTCCACAACCGCAACCACA------GTATTCGCAACCACAATAAC
Td-alpha-A4       CCACAACAACCATATC--CACAACCGCAACGAAA------GTATTCGCAACCACAACATC
Td-alpha-A5       CCACAACAACCATATC--CACAACCGCAACCACA------GTATTCGCAACCACAACAAC
Td-alpha-A2       CCACAACAACCATATC--CACAACCGCAACCACA------GTATCCGCAACCACAACAAC
Td-alpha-A3       CCACAACAACCATATC--CACAACCGCAACCACA------GTATTCGCAACCACAACAAC
Ta-alpha-A1       CCACAACAACCATATC--CACAACCGCAAACACA------GCATCTGCAACCACAACAAC
Ta-alpha-A2       CCACAACAACCATATC--CACAACCGCAAACACA------TCATCTGCAACCACAACAAC
Td-alpha-A1       CCACAACAACCATATC--CACAACCGCAAACACA------GCATCTGCAACCACAACAAC
Td-alpha-A19      CCACAACAACCATATC--CACAACCGCAACCACA------GTATTCGCAACCACAACAAC
Td-alpha-A7       CAACAACAACAACA-----ACAACAACAACAACA------------ACAACAACAACAAC
Td-alpha-A6       -----------------------------------------------CAACAACAACAAC
Td-alpha-A16      CCACAACAACCATATC--CACAACCGCAACCACAT------TATTCGCAACCACAAGAAC

Ta-alpha-A4       CAATTTCACAGCAGCAGCAGCAGCA-----------------------------------
Ta-alpha-A5       CAATTTCACAGCAGCAGCAGCAGCAGCA--ACAACAACAACAACAACAACAACAACAACA
Td-alpha-A14      CAATTTCACAGCAGCAGCAGCAGCAGCA--ACAACAACAACAACAACAACAACAACAACA
Td-alpha-A20      CAATTTCACAGCAGCAGCAGCAACAACA--ACA---------------------------
Ta-alpha-A7       CAATTTCACAGCATCAACAGCAGCAGCA--GCAACAACAACAACAACAACAACA------
Ta-alpha-A9       CAATTTCACAGCAGCAGCAGCAGCAGCA--GCAGCAGCAACAACAACAACAACA------
Ta-alpha-A8       CAATTTCACAGCAGCAGC------AGCA--GCAGCAACAACAACAACAACA---------
Ta-alpha-A10      CAATTTCACAGCAGCAGC------AACA--ACAACAACAACAACAACAA-----------
Ta-alpha-A6       CAATTTCACAGCAGCAGC------AGCA--GCAGCAACAACAACAACAACA---------
Td-alpha-A15      CAATTTCACAGCAGCAGC------AGCA--GCAGCAACAACAACAACAACA---------
Td-alpha-A21      CAATTTCACAGCAGCAGCCGCAGCAGCA--GCAACAACAACAACAACAACA---------
Td-alpha-A24      CAATTTCACAGCAGCAGCAGCAACAACA--ACAACAACAATAACAACA------------
Td-alpha-A13      CAATTTCACAGCAGCAGCAGCAACAACA--ACAACAACAAGAACAACAA-----------
Td-alpha-A17      CAATTTCACAGCAGCAGCAACAGCAGCA--GCAGCAACAACAACAACAACA---------
Td-alpha-A22      CAATTTCACAGCAGCAGCAGCAGCAGCA--ACAACAACAACAACAACAACAACA------
Td-alpha-A23      CAATTTCACAGCAGCAGCAGCAGCAGCA--ACAACAACAACAACAACA------------
Td-alpha-A18      CAATTTCACAGCAGCAGCAGCAACAACA--ACAACAACAACAACAACA------------
Td-alpha-A11      CAATTTCGCAGCAACAATAACAACAACA--ACAA--------------------------
Td-alpha-A9       CAATTTCGCAGCAACAATAACAACAACA--ACAACAACAACAACAA--------------
```

```
Td-alpha-A10      CAATTTCGCAGCAACAATAACAACAACA--ACAA--------------------------
Td-alpha-A8       CAATTTCGCAGCAACAATAACAACAACA--ACAACAACAACAACAA--------------
Td-alpha-A12      CAATTTCGCAGCAGCAGCAACAACAACA--ACAACAACAACAACAA--------------
Ta-alpha-A3       CAATTTCGCAGCAGCAACAACAACAACA--ACAACAACAACAACAACAA-----------
Td-alpha-A4       CAATTTCGCATCAGCAGCAGCAGCAACA--ACAGCAACAACAACAACAATGAC-------
Td-alpha-A5       CAATTTCGCATTAGCAGCAGCAGCAACA--ACAGCAACAACAACAACAATGAC-------
Td-alpha-A2       CAATTTCATAGTAACAAGCACAACAACA--ACAACAACAACAACAACAACAAC-------
Td-alpha-A3       CAATTTCGCATCAGCAGCAGCAACAACA--ATAACAACAACAACAACAACAACAACAACA
Ta-alpha-A1       CAATTTCGCAGCAACAAGCACAACAACA--ACAACAACAACAACAACAACAACAA-----
Ta-alpha-A2       CAATTTCGCAGCAACAAGTACAACAACA--ACAACAACAACAACAACAACAACAACAACA
Td-alpha-A1       CAATTTCGCAGCAACAAGCACAACAACA--ACAACAACAACAACAACAACAACAACAACA
Td-alpha-A19      CAATTTCACAGCAGCAGCAGCAGCAGCA--------------------------------
Td-alpha-A7       AA------CAACAACAACAACAACAACA--ACAACAACAACAACAACA------------
Td-alpha-A6       AA------CAACAACAACAACAACAACA---CAGCAACAA--------------------
Td-alpha-A16      CAATTTCACAGCAGCAGCAGCAACAACA--ACAACAACAACAACAA--------------

Ta-alpha-A4       ---------------------------------ACAACAACAACAACAACAAGAACAACA
Ta-alpha-A5       ACAACAACAACAACAACAACAACAACAACAACAACAACAACAACAACAACAAGAACAACA
Td-alpha-A14      ACAACAACA------------------------ACAACAACAACAACAAGAAGAACAACA
Td-alpha-A20      ---------------------------------ACAACAACAACAACAACAACAACAACA
Ta-alpha-A7       ---------------------------------ACAACAA--------------------
Ta-alpha-A9       ---------------------------------ACAACAACAACAACAACAACCACAACA
Ta-alpha-A8       ---------------------------------ACAACAACAACAACAAAAGCAA-----
Ta-alpha-A10      ------------------------------------------------------------
Ta-alpha-A6       ---------------------------------ACAACAACAACAA--------------
Td-alpha-A15      ---------------------------------ACAACAACAACAACAACAACAACAACA
Td-alpha-A21      ---------------------------------ACAACAACAACAACAACAAGAACAACA
Td-alpha-A24      ---------------------------------ACAACAACAA-----------------
Td-alpha-A13      ------------------------------------------------------------
Td-alpha-A17      ---------------------------------ACAACAACAACAACAA---------CA
Td-alpha-A22      ---------------------------------ACAACAACAACAACAACAACAACAGCA
Td-alpha-A23      ---------------------------------ACAACAACAA-----------------
Td-alpha-A18      ---------------------------------ACAACAACAACAA--------------
Td-alpha-A11      ------------------------------------------------------------
Td-alpha-A9       ------------------------------------------------------------
Td-alpha-A10      ------------------------------------------------------------
Td-alpha-A8       ------------------------------------------------------------
Td-alpha-A12      ------------------------------------------------------------
Ta-alpha-A3       ------------------------------------------------------------
Td-alpha-A4       -----------------------------------------------------AAAAACA
Td-alpha-A5       -----------------------------------------------------AAAAACA
Td-alpha-A2       --------------------------------------------------AACAACAACA
Td-alpha-A3       ACAACAACA------------------------ACAACAACAACAACAACAACAACAACA
Ta-alpha-A1       ------------------------------------------------------------
Ta-alpha-A2       ACAACAACAACAACAACAACA------------ACAACAACAACTACTACTACAACAACA
Td-alpha-A1       ACAACAATAACAAC------------------------------------TACAACAACA
Td-alpha-A19      ------------------------------------------------------------
Td-alpha-A7       ----------------------------------------------------CAGCAACA
Td-alpha-A6       ------------------------------------------------------------
Td-alpha-A16      ------------------------------------------------------------

Ta-alpha-A4       AATACTTCAACAAATTTTGCAACAACAAC---TGATTCCATGCATGGATGTT---GTATT
Ta-alpha-A5       AATCCTTCAACAAATTTTGCAACAACAAC---TAATTCCATGCATGGATGTT---GTATT
Td-alpha-A14      AATCCTTCAACAAATTTTGCAACAACAAC---TGATTCCATGCATGGATGTT---GTATT
Td-alpha-A20      AATCCTTCAACAAATTTTGCAACAACAAC---TGATTCCATGCATGGATGTT---GTATT
Ta-alpha-A7       -ATCCTTCAACAAATTTTGCAACAACAAC---TGATTCCATGCATGGATGTT---GTATT
Ta-alpha-A9       AATCCTTCAACAAATTTTGCAACAACAAC---TGATTCCATGCATGGATGTT---GTATT
Ta-alpha-A8       -ATCCTTCAACAAATTTTGCAACAACAAC---TGATTCCATGCATGGATGTT---GTATT
Ta-alpha-A10      -ATCCTTCAACAAATTTTGCAACAACAAC---TGATTCCATGCATGGATGTT---GTATT
```

```
Ta-alpha-A6       -ATCCTTCAACAAATTTTGCAACAACAAC---TGATTCCATGCATGGATGTT---GTATT
Td-alpha-A15      AATCCTTCAACAAATTTTGCAACAACAAC---TGATTCCATGCATGGATGTT---GTATT
Td-alpha-A21      AATCCTTCAACAAATTTTGCAACAACAAC---TGATTCCATGCATGGATGTT---GTATT
Td-alpha-A24      -ATCCTTCAACAAATTTTGCAACAACAAC---TGATTCCATGCATGGATGTT---GTATT
Td-alpha-A13      -ATCCTTCAACATATTTTGCAACAACAAC---TGATTCCATGCATGGATGTT---GTATT
Td-alpha-A17      AATCCTTCAACAAATTTTGCAACAACAAC---TGATTCCATGCATGGATGTT---GTATT
Td-alpha-A22      AATCCTTCAACAAATTTTGCAACAACAAC---TGATTCCATGCATGGATGTT---GTATT
Td-alpha-A23      -ATCCTTCAACAAATTTTGCAACAACAAC---TGATTCCATGCATGGATGTT---GTATT
Td-alpha-A18      -ATCCTTCAACAAATTTTGCAACAACAAC---TGATTCCATGCATGGATGTT---GTATT
Td-alpha-A11      -ATCCTTCAACAAATTTTGCAACAACAAC---TGATTCCATGCATGGATGTT---GTATT
Td-alpha-A9       -ATCCTTCAACAAATTTTGCAACAACAAC---TGATTCCATGCATGGATGTT---GTATT
Td-alpha-A10      -ATCCTTCAACAAATTTTGCAACAACAAC---TGATTCCATGCATGGATGTT---GTATT
Td-alpha-A8       -ATCCTTCAACAAATTTTGCAACAACAAC---TGATTCCATGCATGGATGTT---GTATT
Td-alpha-A12      -ATCCTTCAACAAATTTTGCAACAACAAC---TGATTCCATGCATGGATGTT---GTATT
Ta-alpha-A3       -ATCCTTCAACAAATTTTGCAACAACAAC---TGATTCCATGCATGGATGTT---GTATT
Td-alpha-A4       AATCCTTCAACAAATTTTGCAACAACATC---TCATTCCATGCATGGATGTT---GTATT
Td-alpha-A5       AATCCTTCAACAAATTTTGCAACAACAAC---TCATTCCATGCATGGATGTT---GTATT
Td-alpha-A2       AATCCTTCAACAAATTTTGCAACAACAAC---TGATTCCATGCAGGGATGTT---GTCTT
Td-alpha-A3       AATCCTTCAACAAATTTTGCAACAACAAC---TGATTCCATGCATGGATGTT---GTATT
Ta-alpha-A1       -ATCCTTCAACAAATTCTGCAACAACAACAACTGATTCCATGCAGGGATGTCATCGTCTT
Ta-alpha-A2       AATCCTTCAACAAATTCTGCAACAACAACAACTGATTCCATGCAGGGATGTCATCGTCTT
Td-alpha-A1       AATCCTTCAACAAATTCTGCAACAACAAC---TGATTCCATGCAGGGATGTCATCGTCTT
Td-alpha-A19      ------------------------------------------------------------
Td-alpha-A7       AATCCTTCAACAAATTTTGCAACAACAAC---TGATTCCATGCATGGATGTT---GTATT
Td-alpha-A6       -ATCCTTCAACAAATTTTGCAACAACAAC---TGATTCCATGCATGGATGTT---GTATT
Td-alpha-A16      -------------------CAACAACAAC-------------------------------

Ta-alpha-A4       GCAGCAACACAACATAGCGCATGGAAGATCACAAGTTTTGCAACAAAGTACTTACCAGCT
Ta-alpha-A5       ACAGCAACACAACATAGCGCATGGAAGATCACAAGTTTTGCAACAAAGTACTTACCAGCT
Td-alpha-A14      GCAGCAACACAACATAGCGCATGGAAGATCACAAGTTTTGCAACAAAGTACTTACCAGCT
Td-alpha-A20      GCAGCAACACAACATAGCGCATGGAAGATCACAAGTTTTGCAACAAAGTACTTACCAGCT
Ta-alpha-A7       GCAGCAACACAACATAGCGCAAGGAAGATCACAAGTTTTGCAACAAAGTACTTACCAGCT
Ta-alpha-A9       GCAGCAACACAACATAGCGCAAGGAAGATCACAAGTTTTGCAACAAAGTACTTACCAGCT
Ta-alpha-A8       GCAGCAACACAACATAGCGCATGGAAGATCACAAGTTTTGCAACAAAGTACTTACCAGCT
Ta-alpha-A10      GCAGCAACACAACATAGCGCATGGAAGATCACAAGTTTTGCAACAAAGTACTTACCAGCT
Ta-alpha-A6       GCAGCAACACAACATAGCGCATGGAAGATCACAAGTTTTGCAACAAAGTACTTACCAGCT
Td-alpha-A15      GCAGCAACACAACATAGCGCATGGAAGATCACAAGTTTTGCAACAAAGTACTTACCAGCT
Td-alpha-A21      GCAGCAACACAACATAGCGCATGGAAGATCACAAGTTTTGCAACAAAGTACTTACCAGCT
Td-alpha-A24      GCAGCAACACAACATAGCGCATGGAAGATCACAAGTTTTGCAACAAAGTACTTACCAGCT
Td-alpha-A13      GTAGCAACACAACATAGCGCATGGAAGATCATAAGTTTTGCAACAAAGTACTTACCAGCT
Td-alpha-A17      GCAGCAACACAACATAGCCCATGGAAGATCACAAGTTTTGCAACAAAGTACTTACCAGCT
Td-alpha-A22      GCAGCAACACAACATAGCGCATGGAAGATCACAAGTTTTGCAACAAAGTACTTACCAGCT
Td-alpha-A23      GCAGCAACACAACATAGCGCATGGAAGATCACAAGTTTTGCAACAAAGTACTTACCAGCT
Td-alpha-A18      GCAGGAACACAACATAGCGCATGGAAGATCACAAGTTTTGCAACAAAGTACTTACCAGCT
Td-alpha-A11      GCAGCAACACAACATAGCGCATGGAAGATCACAAGTTTTGCAACAAAGTACTTACCAGCT
Td-alpha-A9       GCAGCAACACAACATAGCGCATGGAAGATCACAAGTTTTGCAACAAAGTACTTACCAGCT
Td-alpha-A10      GCAGCAACACAACATAGCGCATGGAAGATCACAAGTTTTGCAACAAAGTACTTACCAGCT
Td-alpha-A8       GCAGCAACACAACATAGCGCATGGAAGATCACAAGTTTTGCAACAAAGTACTTACCAGCT
Td-alpha-A12      GCAGCAACACAACATAGCGCATGGAAGACCACAAGTTTTGCAACAAAGTACTTACCAGCT
Ta-alpha-A3       GCAGCAACACAACATAGCGCATGGAAGATCACAAGTTTTGCAACAAAGTAGTTACCAGCT
Td-alpha-A4       GCAGCAACACAACATAGCGCATGGAAGCTCACAAGTTTTGCAACAAAGTACTTACCAGTT
Td-alpha-A5       GCAGCAACACAACATAGCGCATGGAAGCTCACAAGTTTTGCAACAAAGTACTTACCAGTT
Td-alpha-A2       GCAACAACACAACATAGCGCATGCAAGATCACAAGTTTTGCAACAAAGCACTTACCAGCC
Td-alpha-A3       GCAGCAACACAACATAGCGCATGGAAGCTCACAAGTTTTGCAACAAAGTACTTACCAGTT
Ta-alpha-A1       GCAACAACACAACATAGCGCATGAAAGCTCACAAGTATTGCAGCAAAGTAGTTACCAAGT
Ta-alpha-A2       GCAACAACACAACATAGCGCATGAAAGCTCACAAGTATTGCAGCAAAGTAGTTACCAAGT
Td-alpha-A1       GCAACAACACAACATAGTGCATGAAAGCTCACAAGTATTGCAGCAAAGTAGTTACCAAGT
Td-alpha-A19      ------------------------------------------------------------
Td-alpha-A7       GCAACAACACAACATAGCGCATGGAAGCTCACAAGTTTTGCAACAAAGTACTTACCAGTT
```

```
Td-alpha-A6        GCAACAACACAACATAGCGCATGGAAGCTCACAAGTTTTGCAACAAAGTACTTACCAGTT
Td-alpha-A16       ------------------------------------------------------------

Ta-alpha-A4        GTTGCAAGAATTGTGTTGTCAGCACCTATGGCAGATCCCTGAGCAGTCGCAGTGCCAGGC
Ta-alpha-A5        GTTGCAAGAATTGTGTTGTCAGCACCTATGGCAGATCCCTGAGCAGTCGCAGTGCCAGGC
Td-alpha-A14       GTTGCAAGAATTGTGTTGTCAGCACCTATGGCAGATCCCTGAGCAGTCGCAGTGCCAGGC
Td-alpha-A20       GTTGCAAGAATTGTGTTGTCAGCACCTATGGCAGATCCCTGAGCAGTTGCAGTGCCAGGC
Ta-alpha-A7        GTTGCAAGAATTGTGTTGTCAGCACCTATGGCAGATCCCTGAGCAGTCGCAGTGCCAGGC
Ta-alpha-A9        GTTGCAAGAATTGTGTTGTCAGCACCTATGGCAGATCCCTGAGCAGTCGCAGTGCCAGGC
Ta-alpha-A8        GTTGCAAGAATTGTGTTGTCAGCACCTATGGCAGATCCCTGAGCAGTCGCAGTGTCAGGC
Ta-alpha-A10       ATTGCAAGAATTGTGTTGTCAGCACCTATGGCAGATCCCTGAGCAGTCGCAGTGCCAGGC
Ta-alpha-A6        GTTACAAGAATTGTGTTGTCAGCACCTATGGCAGATCCCTGAGCAGTCGCAGTGCCAGGC
Td-alpha-A15       GTTGGAAGAATTGTGTTGTCAGCACCTATGGCAGATCCCTGAGCAGTCGCAGTGCCAGGC
Td-alpha-A21       GTTGCAAGAATTGTGTTGTCAACACCTATGGCAGATCCCTGAGCAGTCGCAGTGCCAGGC
Td-alpha-A24       GTTGCAAGAATTGTGTTGTCAGCACCTATGGCAGATCCCTGAGCAGTCGCAGTGCCAGGC
Td-alpha-A13       GTTGCAAGCATTGTGTTGTCAGCACCTATGGCATATCCCTGAGCAGTCGCAGTGCCAGGC
Td-alpha-A17       GCTGCAAGAATTGTGTTGTCAGCACCTATGGCAGATCCCTGAGCAGTCGCAGTGCCAGGC
Td-alpha-A22       GTTGCAAGAATTGTGTTGTCAGCACCTATGGCAGATCCCTGAGCAGTCGCAGTGCCAGGC
Td-alpha-A23       GCTGCAAGAATTGTGTTGTCAACACCTATGGCAGATCCCTGAGCAGTCGCAGTGCCAGGC
Td-alpha-A18       GTTGCAAGAATTGTGTTGTCAGCACCTATGCAAGATCCCTGAGCAGTCGCAGTGCCAGGC
Td-alpha-A11       GTTGCAAGAATTGTGTTGTCAGCACCTATGGCAGATCCCTGAGCAGTCGCAGTGCCAAGC
Td-alpha-A9        GTTGCAAGAATTGTGTTGTCAGCACCTATGGCAGATCCCTGAGCAGTCGCAGTGCCAAGC
Td-alpha-A10       GTTGCAAGAATTGTGTTGTCAGCACCTATGGCAGATCCCTGAGTAGTCGCAGTGCCAAGC
Td-alpha-A8        GTTGCAAGAATTGTGTTGTCAGCACCTATGGCAGATCCCTGAGCAGTCGGAGTGCCAAGC
Td-alpha-A12       ATTG---GAATTGTGTTTTCAGCACCTATGACAGATCCCTGAGCAGTCGCAGTGCCAAGC
Ta-alpha-A3        GTTGCAAGAATTGTGTTTTCAGCACCTATGACAGATCCCTGAGCAGTCGCAGTGCCAAGC
Td-alpha-A4        GTTGCAAGAATTGTGTTGTCAGCACCTATGGCAGATCCCTGAGCAGTCGCAGTGCCAAGC
Td-alpha-A5        GTTGCAAGAATTGTGTTGTCAGCACCTATGGCAGATCCCTGAGAAGTCGCAGTGCCAAGC
Td-alpha-A2        ATTGCAACAATTGTGTTGTCAACAGCTGTGGCAGATCCCCGAGCAGTCGCAGTGCCAAGC
Td-alpha-A3        GATGCAAGAATTGTGTTGTCAACACCTATGGCAGATCCCTGAACAGTCGCAGTGCCAAGC
Ta-alpha-A1        GTTGCAACAATTATGTTGTCAGCAGCTGCGGCAGATCCCCGAGCAGTCGCGGTGCCAAGC
Ta-alpha-A2        GTTGCAACAATTATGTTGTCAGCAGCTGCGGCTGATCCCCGAGCAGTCGCGGTGCCAAGC
Td-alpha-A1        GTTGCAACAATTATGTTGTCAGCAGCTGTGGCAGATCCCTGAGCAGTCGCGGTGTCAAGC
Td-alpha-A19       ------------------------------------------------------------
Td-alpha-A7        GTTGCAAGAATTGTGTTGTCAGCACCTATGGCAGATCCCTGAGCAGTCGCAGTGCCAAGC
Td-alpha-A6        GTTGCAAGAATTGTGTTGTCAGCACCTATGGCAGATCCCTGAGCAGTCGCAGTGCCAAGC
Td-alpha-A16       ------------------------------------------------------------

Ta-alpha-A4        CATCCACAATGTTGTTCATGCTATTATTCTGCGTCAACAACAAAAA------------CA
Ta-alpha-A5        CATCCACAATGTTGTTCATGCTATTATTCTGCATCAACAACAAAAA------------CA
Td-alpha-A14       CATCCACAATGTTGTTCATGCTATTATTCTGCATCAACAACAAAAA------------CA
Td-alpha-A20       CATCCACAATGTTGTTCATGCTATTATTCTGCATCAACAACAAAAA------------CA
Ta-alpha-A7        CATCCACAATGTTGTTCATGCTATTATTCTGCATCAACAACAACAAAAACAACAACAACA
Ta-alpha-A9        CATCCACAATGTTGTTCATGCTATTATTCTGCATCAACAACAAAAACAACAACAACAACA
Ta-alpha-A8        CATCCAAAATGTTGTTCATGCTATTATTCTGCATCAACAACAAAAA--------------
Ta-alpha-A10       CATCCAAAATGTTGTTCATGCTATTATTCTGCATCAACAACAAAAA--------------
Ta-alpha-A6        CATCCACAATGTTGTTCATGCTATTATTCTGCATCAACAACAAAAA--------------
Td-alpha-A15       CATCCACAATGTTGTTCATGCTATTATTCTGCATCAACAACAAAAA--------------
Td-alpha-A21       CATCCACAATGTTGTTCATGCTATTATTCTGCATCAACAACAAAAA--------------
Td-alpha-A24       CATCCACAATGTTGTTCATGCTATTATTCTGCATCAACAACAAAAA--------------
Td-alpha-A13       CATCCACAATGTTGTTCATGCTATTATTCTGCATCAACAACAAAAA--------------
Td-alpha-A17       CATCCACAATGTTGTTCATGCTATTATTCTGCATCAACAACAAAAA--------------
Td-alpha-A22       CATCCACAATGTTGTTCATGCTATTATTCTGCATCAACAACAAAAA--------------
Td-alpha-A23       CATCCACAATGTTGTTCATGCTATTATTCTGCATCAACAACAAAAA--------------
Td-alpha-A18       CATCCACAATGTTGTTCATGCTATTATTCTGCATCAACAACAAAAA------------CA
Td-alpha-A11       CATCCACAATGTTGTCCATGCTATTATTCTGCATCAACAACAAAAA---------CAACA
Td-alpha-A9        CATCCACAATGTCGTCCATGCTATTATTCTGCATCAACAACAAAAA---------CAACA
Td-alpha-A10       CATCCACAATGTTGTCCATGCTGTTATTCTGCATCAACAACAAAAA---------CAACA
Td-alpha-A8        CATCCACAATGTCGTCCATGCTATTATTCTGCATCAACAACAAAAA---------CAACA
```

```
Td-alpha-A12      CATCCACAATGTTGTCCATGCTATTATTCTGCATCAACAACAAAAG---------CAACA
Ta-alpha-A3       CATCCACAATGTTGTCCATGCTATTATTCTGCATCAACAACAAAAACAACAACAACAACA
Td-alpha-A4       CATCCAGAATGTTGTTCATGCTATTATTCTGCATCAACAACAAAAA--------------
Td-alpha-A5       CATCCACAATGTTGTTCATGCTATTATTCTGCATCAACAACAAAAA--------------
Td-alpha-A2       CATCCACAATGTTGTTCATGCTATTATTCTGCATCAACAACAG-----------------
Td-alpha-A3       CATCCACAATGTTGTTCATGCTATTATTCTGCATCAACAACAAAAA--------------
Ta-alpha-A1       CATCAACAATGTCGTTCATGCTATTATTCTGCATCAACAACAACAACAACAA--------
Ta-alpha-A2       CATCCACAATGTCGTTCATGCTATTATTCTACATCAACAACAACAACAACAACAACAACA
Td-alpha-A1       CATCCACAATGTCGTTCATGCTATTATTCTGCATCATCATCATCAACAACAACAACAACA
Td-alpha-A19      ------------------------------------------------------------
Td-alpha-A7       CATCCACAATGTTGTTCATGCTATTATTCTGCATCAACAACAAAAA------------CA
Td-alpha-A6       CATCCACAATGTTGTTCATGCTATTATTCTGCATCAACAACAAAAA------------CA
Td-alpha-A16      --------------------------------AACAACAACAACAA--------------

Ta-alpha-A4       ACAAC------AA---CAAC---CATCGAGCCAGGTCTCCTTCCAACAGCCTCAGCAACA
Ta-alpha-A5       ACAAC------AA---CAACAACCATCGAGCCAGGTCTCCTTCCAACAGCCTCAACAACA
Td-alpha-A14      ACAAC------AA---CAAC---TATCGAGCCAGGTCTCCTTCCAACAGCCTCAGCAACA
Td-alpha-A20      AGAAC------AA---CAACAACTATCGAGCCAGGTCTCCTTCCAACAGCCTCAGCAACA
Ta-alpha-A7       ACAAC------AA---CAACAACCATCGAGCCAGGTCTCCTTCCAACAGCCTCAGCAACA
Ta-alpha-A9       ACAAC------AA---CAACAACCATCGAGTCAGGTCTCCTTCCAACAGCCTCAGCAACA
Ta-alpha-A8       -CAAC------AA---CAACAACCATCGAGCCAGGTGTCCTTCCAACAGCCTCTGCAACA
Ta-alpha-A10      -CAAC------AA---CAACAACCATCGAGCCAGGTCTCCTTCCAACAGCCTCTGCAACA
Ta-alpha-A6       -CCAC------AA---CAACAACCATCGAGCCAGGTCTCCTTCCAACAGCCTCTGCAACA
Td-alpha-A15      -CAAC------AA---CAACAACCATCGAGCCAGGTCTCCTTCCAACAGCCTCTACAACA
Td-alpha-A21      -CAAC------AA---CAACAACCATCGAGCCAGGTCTCCTTCCAACAGCCTCTGCAACA
Td-alpha-A24      ----------------CAACAACCATCGAGCCAGGTCTCCTTCCAACAGCCTCAGCAACA
Td-alpha-A13      -CAAC------AA---CAACAACCATCGAGCCAGGTCTCCTACCAACAGCCTCAGCAACA
Td-alpha-A17      -CAAA------AA---CAACAACCATCGAGCCAGGTCTCCTTCCAACAGCCTCTGCAACA
Td-alpha-A22      -CAAC------AA---CAACAACCATCGAGCCAGGTCTCCTTCCAACAGCCTCTGCAACA
Td-alpha-A23      -CAAC------AA---CAACAACCATCGAGCCAGGTCTCCTTCCAACAGCCTCTGCAACA
Td-alpha-A18      ACAAC------AA---CAACAACCATCGAGCCAGGTCTCCT-------------------
Td-alpha-A11      ACAAC------AA---CAACAACCATCGAGCCAGGTCTCCTTCCAACAGCCTCAGCAACA
Td-alpha-A9       ACAAC------AA---CAAC---CATCGAGCAAGGTCTCCTTCCAACAGCCTCAGCAACA
Td-alpha-A10      ACAAC------AA---CAAC---CATCGAGCCAGGTCTCCTTCCAACAGCCTCAGCAACA
Td-alpha-A8       ACAAC------AA---CAACAACCATCGAGCAAGGTCTCCTTCCAACAGCCTCAGCAACA
Td-alpha-A12      ACAAC------AA---CAACAACCATCGAGCCAGGTCTCCTTCCAATAGCCTCAGCAACA
Ta-alpha-A3       ACAAC------AAAAACAACAACCATCGAGCCAGGTCTCCTTCCAACAGCCCCAGCAACA
Td-alpha-A4       ----C------AACAACAACAACCATTGAGCCAGGTCTCCTTCCAACAGCCTCAGCAACA
Td-alpha-A5       ----C------AACAACAACAACCATTGAGCCAGGTCTCCTTCCAACAGCCTCAGCAACA
Td-alpha-A2       ----C------GACAACAACAACCATCGAGCCAAGTCTCCTTCCAACAGCCTCAGCAACA
Td-alpha-A3       ----C------AACAACAACAACCATCGACCCATGCCTCCTTCCAACAGCCTCAACAACA
Ta-alpha-A1       ----------GGACAACATCAACAGTCGAGCCAGGTCTCCTACCAGCAGCCTCAGCAACA
Ta-alpha-A2       ACAACAACAAGGACAACATCAACCGTCGAGCCAGGTCTCCTACCAGCAGCCTCAGCAACA
Td-alpha-A1       AGAA------GAACAACAACAACCGTCGAGCCAGGTCTCCTACCAGCAGCCTCAACAACA
Td-alpha-A19      NNNNNAACAACAACAACAACAACCATCGAGCCAGGTCTCCTTCCAACAGCCTCAGCAACA
Td-alpha-A7       ACAAC------AACAACAACAACCATCGAGCCAGGTCTCCTTCCAACAGCCTCAGCAACA
Td-alpha-A6       ACAAC------AACAACAACAACCATCGAGCCAGGTCTCCTTCCAACAGCCTCAGCAACA
Td-alpha-A16      -------------------------------------------CAACAGCAGCAGCAGCA

Ta-alpha-A4       ATATCCATTAGGCCAGGGCTCCTTCCGGCCATCTCAGCAAAACCCACAGGCCCAGGGCTC
Ta-alpha-A5       ATATCCATTAGGCCAGGGCTCCTTCCGGCCATCTCAGCAAAACCCACAGGCCCAGGGCTC
Td-alpha-A14      ATATCCATTAGGCCAGGGCTCCTTCCGGCCATCTCAGCAAAACCCACAGGCCCAGGGCTC
Td-alpha-A20      ATATCCATTAGGCCAGGTCTCCTTCCGGCCATCTCAGCAAAACCCACAGGCCCAGGGCTC
Ta-alpha-A7       ATATCCATTAGGCTAGGGCTCCTTCCGGCCATCTCAGCAAAACCCACAGGCCCAGGGCTC
Ta-alpha-A9       ATATCCATTAGGCCAGGGCTCCTTCCGGCCATCTCAGCAAAACCCACAGGCCCAGGGCTC
Ta-alpha-A8       ATATCCATTAGGCCAGGGCTCCTTCCGGCCATCTCAGCAAAACCCACAGGACCAGGGCTC
Ta-alpha-A10      ATATCCATTAGGCCAGGGCTCCTTCCGGCCATCTCAGCAAAACCCACAGGACCAGGGCTC
Ta-alpha-A6       ATATCCATTAGGCCAGGGCTCCTTCCGGCCATCTCAGCAAAACCCACAGGCCCGGGGCTC
Td-alpha-A15      ATATCCATTAGGCCAGGGCTCCTTCCGGCCATCTCAGCAAAACCCACAGGCCCAGGGCTC
```

```
Td-alpha-A21      ATATCCATTAGGCCAGGGCTCCTTCCGGCCATCTCAGCAAAACCCACAGGCCCAGGGCTC
Td-alpha-A24      ATATCCATTAGGCCAGGGCTCCTTCTGGCCATCTCAGCAAAACCCACAGGCCCAGGGCTC
Td-alpha-A13      ATATCCATTAGGCCAGGGCTCCTTCCGGCCATCTCAGCAAAACCCACAGGCCCAGGGCTC
Td-alpha-A17      ATATCCATTAGGCCAGGGCTCCTTCCGGCCATCTCAGCAAAACCCACAGGCCCAGGGCTC
Td-alpha-A22      ATATCCATTAGGCCAGGGCTCCTTCCGGCCATCTCAGCAAAACCCACAGGCCCAGGGCTC
Td-alpha-A23      ATATCCATTAAGCCAGGGCTCCTTCCGGCCATCTCAGCAAAACCCACAGGCCCAGGGCTC
Td-alpha-A18      ---TCCATTAAGCCAGGGCTCCTTCCGGCCATCTCAGCAAAACCCACAGGCCCAGGACTC
Td-alpha-A11      ATATCCATCAGGCCAGGGCTCCTTCCGGCCATCTCAGCAAAACCCACAGGCCCAGGGCTC
Td-alpha-A9       ATATCCATCAGGCCAGGGCTCCTTCCGGCCATATCAGCAAAACCCACAGGCCCAGGGCTC
Td-alpha-A10      ATATCCATCAGGCCAGGGCTCCTTCCGGCCATCTCAGCAAAACCCACAGGCCCAGGGCTC
Td-alpha-A8       ATATCCATCAGGCCAGGGCTCCTTCCGGCCATATCAGCAAAAACCACAGGCCCAGGGCTC
Td-alpha-A12      ATATCCATCAGGCCAGGGCTCCTTCCGGCCATCTCAGCAAAACCCACAGGCCCAGGGTTC
Ta-alpha-A3       ATATCCATCAGGCCAGGGCTCCTTCCGGCCATCTCTGCAAAATCCACAGGCCCAGGGCTC
Td-alpha-A4       ATATCCATCAGGCCAGGGCTCCTTTCGGCCATCTCAGCAAAACCCACAGGCCCAGGGCTC
Td-alpha-A5       ATATCCATCAGGCCAGGGCTCCTTTCGGCCATCTCAGCAAAACCCACAGGCCCAGGGCTC
Td-alpha-A2       ATATCCATCAGGTCAGGGCTCCTTCCAGCCATCTCAGCAAAACCCACAGGCCCAGGGCTC
Td-alpha-A3       ATATCCATCAGGCCAGGGCTCCTTTCGTCCATCTCAGCAAAACCCACAGGCCCAGGGCCC
Ta-alpha-A1       ATATCCATCAGGCCAGGGCTCCTTCCAGCCATCTCAGCAAAATCCACAGGCCCAGGGCTT
Ta-alpha-A2       ATATCCATCAGGCCAGGGCTCCTTCCAGCCATCTCAGCAAAATCCACAGGCCCAGGGCTT
Td-alpha-A1       ATATCCATCAGGCCAGGGCTACTTCCAGCCATCTCAGCAAAATCCATAGGCCCAGGGCTT
Td-alpha-A19      ATATCCATTAGGCCAGGGCTCCTTCCGGCCATCTCAGCAAAACCCACAGGCCCAGGGCTC
Td-alpha-A7       ATATTCATCAGGATAGGGCTCCTTTCGGCCATCTCGGCAAAACCCACAGGCCCAGGGCTC
Td-alpha-A6       ATATTCATCAGGNNAGGGCTCCTTTCGGCCATCTCGGCAAAACCCACAGGCCCAGGGCTC
Td-alpha-A16      G---------------------------------CAGCAGCAGCAGCAG-----------

Ta-alpha-A4       TGTCCAGCCTCAACAACTGCCCCAGTTCGAGGAAATAAGGAACCTAGCGCTACAGACGCT
Ta-alpha-A5       TGTCCAGCCTCAACAACTGCCCCAGTTCGAGGAAATAAGGAACCTAGCGCTACAGACGCT
Td-alpha-A14      TGTCCAGCCTCAACAACTGCCCCAGTTCGAGGAAATAAGGAACCTAGCGCTACAGACGCT
Td-alpha-A20      TGTCCAGCCTCAACAACTGCCCCAGTTCGAGGAAATAAGGAACCTAGCGCTACAGACGCT
Ta-alpha-A7       TGTCCAGCCTCAACAACTGCCCCAGTTCGAGGAAATAAGGAACCTAGCGCTACAGACACT
Ta-alpha-A9       TGTCCAGCCTCAACAACTGCCCCAGTTCGAGGAAATAAGGAACCTAGCGCTACAGACACT
Ta-alpha-A8       TGTCCAGCCTCAACAACTGCCCCAGTTCGAGGAAATAAGGAACCTAGCGCTACAGATGCT
Ta-alpha-A10      TGTCCAGCCTCAACAACTGCCCCAGTTCGAGGAAATAAGGAACCTAGCGCTACAGACGCT
Ta-alpha-A6       TGTCCAGCCTCAACAACTGCCCCAGTTCGAGGAAATAAGGAACCTAGCGCTACAGACGCT
Td-alpha-A15      TGTCCAGCCTCAACAACTGCCCCAGTTCGAGGAAATAAGGAACCTAGCGCTACAGACGCT
Td-alpha-A21      TGTCCAGCCTCAACAACTGCCCCAGTTCGAGGAAATAAGGAACCTAGCGCTACAGACGCT
Td-alpha-A24      TGTCCAGCCTCAACAACTGCCCCAGTTCGAGGAAATAAGGAACCTAGCGCTACATACGCT
Td-alpha-A13      TGTCCAGCCTCAACAACTGCCCCAGTTCGAGGAAATAAGGAACCTAGCGCTATAGACGCT
Td-alpha-A17      TGTCCAGCCTCAACAACTGCCCCAGTTCGAGGAAATAAGGAACCTAGCGCTACAGACGCT
Td-alpha-A22      TGTCCAGCCTCAACAACTGCCCCAGTTCGAGGAAATAAGGAACCTAGCGCTACAGACGCT
Td-alpha-A23      TGTCCAGCCTCAACAACTGCCCCAGTTCGAGGAAATAAGGAACCTAGCGCTACAGACGCT
Td-alpha-A18      TGTCCAGCCTCAACAACTGCCCCAGTTCGAGGAAATAAGGAACCTAGCGCTACAGACGCT
Td-alpha-A11      TGTCCAGTCTCAACAACTGCCCCAGTTCGAGGAAATAAGGAACCTAGCGCTACAGACGCT
Td-alpha-A9       TGTCCATCCTCAACAACTGCCCCAGTTCGAGGAAATAAGTAACCTAGCGCTACAGACGCT
Td-alpha-A10      TGTCCAGCCTCAACAACTGCCCCAGTTCGAGGAAATAAGGAACCTAGCGCTACAGACGCT
Td-alpha-A8       TGTCCAGCCTCAACAACTGCCCCAGTTCGAGGAAATAAGGAACCTAGCGCTACAGACGCT
Td-alpha-A12      TGTCCAGCCTCAACAACTGCCC-AGTTCGAGGAAATAAGGAACCTAGCGCTACAGACGCT
Ta-alpha-A3       GGTCCAGCCTCAACAGCTGCCC-AGTTCGAGGAAATAAGGAACCTAGCGCTACAGACGCT
Td-alpha-A4       TGTCCAGCCTAAACAACTACCCCAGTTCGAGGAAATAAGGAACCTAGCGCTACATACGCC
Td-alpha-A5       TGTCCAGCTTAAACAACTACCCCAGTTCGAGGAAATAAGGAACCTAGCGCTACATACGCC
Td-alpha-A2       TGTCCAACCTCAACAACTGCCCCAGTTCGAGGAAATAAGGAACCTAGCGCTACAGACACT
Td-alpha-A3       TGTCCAGCCTCAACAACTGCCCCA------------------------------------
Ta-alpha-A1       TGTCCAACCTCAACATCTGCCCCAACTCGAGGAAATAAGTAACCTAGCGCTGCAGACGCT
Ta-alpha-A2       TGTCCAACCTCAACATCTGCCCCAACTCGAGGAAATAAGTAACCTAGCGCTGCAGACGCT
Td-alpha-A1       TGTCCAACCTCAACATCTGCCCCAGCTCGAGGAAATAAGGAACCTAGCGCTGCAGACGCT
Td-alpha-A19      TGTCCAGCCTCAACAACTGCCCCAGTTCGAGGAAATAAGGAACCTAGCGCTACAGACGCT
Td-alpha-A7       TGTCCAGCCTCAACAACTGCCCCAGTTCGAGGAAATAAGGAACCTAGCGCTACAGACGCT
Td-alpha-A6       TGTCCAGCCTCAACAACTGCCCCAGTTCGAGGAAATAAGGAACCTAGCGCTACAGACGCT
Td-alpha-A16      ----CAGCAGCAGCAGCAGC----------------------------------------
```

```
Ta-alpha-A4       ACCTGCAATGTGCAATGTCTACATCCCTCCATATTGC------ACCATCGCGCCATTTGG
Ta-alpha-A5       ACCTGCAATGTGCAATGTCTACATCCCTCCATATTGC------ACCATCGTGCCATTTGG
Td-alpha-A14      ACCTGCAATGTGCAATGTCTACATCCCTCCATATTGC------ACCATCGCGCCATTTGG
Td-alpha-A20      ACCTGCAATGTGCAATGTCTACATCCCTCCATATTGC------ACCATCGCGCCATTTGG
Ta-alpha-A7       ACCTGCAATTTGCAATGTCTACATCCCTCCATATTGC------ACCATCGCGCCATTTGG
Ta-alpha-A9       ACCTGCAATTTGCAATGTCTACATCCCTCCATATTGC------ACCATCGCGCCATTTGG
Ta-alpha-A8       ACCTGCAATGTGCAATGTCTACATCCCTCCATATTGC------ACAATCGCGCCATTTGG
Ta-alpha-A10      ACCTGCAATGTGCAATGTCTACATCCCTCCATATTGC------ACCATCGCGCCATTTGG
Ta-alpha-A6       ACCCGCAATGTGCAATGTCTACATCCCTCCATATTGC------ACCATCGCGCCATTTGG
Td-alpha-A15      ACCTGCAATGTGCAATGTCTACATCCCTCCATATTGC------ACCATCACGCCATTTGG
Td-alpha-A21      ACCTGCAATGTGCAATGTCTACATCCCTCCATATTGC------ACCATCGCGCCATTTGG
Td-alpha-A24      ACCTGCAATTTGCAATGTCTACATCCCTCCATATTGC------ACCATCACGCCATTTGG
Td-alpha-A13      ACCTGCAATGTGCAATGTCTACATCCCTCCATATTGC------ACCATCGCGCCATTTGG
Td-alpha-A17      ACCTGCAATGTGCAATGTCTACATCCCTCCATATTGC------ACCATCGCGCCATTTGG
Td-alpha-A22      ACCTGCAATGTGCAATGTCTACATCCCTCCATATTGC------ACCATCGCACCATTTGG
Td-alpha-A23      ACCTGCAATGTGCAATGTCTACATCCCTCCATATTGC------ACCATCGCGCCATTTGG
Td-alpha-A18      ACCTGCAATTTGCAATGTCTACATCCCTCCATATTGC------ACCATCACGCCATTTGG
Td-alpha-A11      ACCTGCAATGTGCAATGTCTACATCCCTCCATATTGC------ACCATCACGCCATTTGG
Td-alpha-A9       ACCTGCAATGTGCAATGTCTACATCCCTCCATATTGC------ACCATCACGCCATTTGT
Td-alpha-A10      ACCTGCAATGTGCAATGTCTACATCCCTCCATATTGC------ACCATCATGCCATTTGG
Td-alpha-A8       ACCTGCAATGTGCAATGTCTACATCCCTTCATATTGC------ACCATCACGCCATTTGT
Td-alpha-A12      ACCTGCAATGTGCAATGTCTACATCCCTCCATATTGC------ACCATCGCGCCATTTGG
Ta-alpha-A3       ACCTGCAATGTGCAATCTCTACATCCCTCCATATTGC------ACCATCGCGCCATTTGG
Td-alpha-A4       ACCTGCAATGTGCAATGTCTACATCCCTCCATATTGC------ACCATCGCGCCATTTGG
Td-alpha-A5       ACCTGCAATGTGCAATGTCTACATCCCTCCATATTGC------ACCATCGCCCCATTTGG
Td-alpha-A2       ACCTGGAATGTGCAATGTCTATATCCCTCCATATTGCTCGACCACCATTGCGCCATTTGG
Td-alpha-A3       ------------------------------------------------------------
Ta-alpha-A1       ACCAGCAATGTGCAATGTCTACATCCCTCCATATTGCTCGACCACCATTGCGCCATTTGG
Ta-alpha-A2       ACCAGCAATGTGCAATGTCTACATCCCTCCATATTGCTCGACCACCATTGCGCCATTTGG
Td-alpha-A1       ACCAGCAATGTGCAATGTCTACATCCCTCCATATTGCTCGACCACCATTGTGCCATTTGG
Td-alpha-A19      ACCTGCAATTTGCAATGTCTACATCCCTCCATATTGC------ACCATCACGCCATTTGG
Td-alpha-A7       ACCTGCAATGTGCAATGTCTACATCCCTCCATATTGC------ACCATCGCGCCATTTGG
Td-alpha-A6       ACCTGCAATGTGCAATGTCTACATCCCTCCATATTGC------ACCATCGCGCCATTTGG
Td-alpha-A16      ------------------------------------------------------------

Ta-alpha-A4       CATCTTTGGTACTAACTAA
Ta-alpha-A5       CATCTTCGGTACTAACTGA
Td-alpha-A14      CATCTTCGGTACTAACTGA
Td-alpha-A20      CATCTTCGGTACTAATTGA
Ta-alpha-A7       CATCTTCGGTACTAACTGA
Ta-alpha-A9       CATCTTCGGTACTAACTGA
Ta-alpha-A8       CATCTTCGGTACTAACTGA
Ta-alpha-A10      CATCTTCGGTACTAACTGA
Ta-alpha-A6       CATCTTCGGTACTAACTGA
Td-alpha-A15      CATCTTCGGTACTAACTGA
Td-alpha-A21      CATCTTCGGTACTAACTGA
Td-alpha-A24      CATCTTCGGTACTAACTGA
Td-alpha-A13      CATCTTCGGTACTAACTGA
Td-alpha-A17      CATCTTCGGTACTAACTGA
Td-alpha-A22      CATCTTCAGTACTAACTGA
Td-alpha-A23      CATCTTCGGTACTAACTGA
Td-alpha-A18      CATCTTCGGTACTAACTGA
Td-alpha-A11      CATCTTCGGTACTAACTGA
Td-alpha-A9       CATCTTCGGTATTAACTGA
Td-alpha-A10      CATCTTCGGTACTAACTGA
Td-alpha-A8       CATCTTCGGTATTAACAGA
Td-alpha-A12      CATATTCGGTACTAACTGA
Ta-alpha-A3       CATCTTCGGTACTAACTGA
```

```
Td-alpha-A4       CATCTTTGGTACTAACTGA
Td-alpha-A5       CATCTTTGGTACTAACTGA
Td-alpha-A2       CATCTTCGGTACTAACTGA
Td-alpha-A3       -------------------
Ta-alpha-A1       CATCTTCGGTACTAACTGA
Ta-alpha-A2       CATCTTCGGTACTAACTGA
Td-alpha-A1       CATCTTCGGTACTAACTGA
Td-alpha-A19      CATCTTCGGTACTAACTGA
Td-alpha-A7       CATCTTTGGTACTAACTGA
Td-alpha-A6       CATCTTTGGTACTAACTGA
Td-alpha-A16      -----------------AA
```
