# Supplementary material for: Rapid evolution of α-gliadin gene family revealed by analyzing Gli-2 locus regions of wild emmer wheat
Source: Funct Integr Genomics. 2019 Jun 13;19(6):993–1005. doi: 10.1007/s10142-019-00686-z (PMC6797660; doi:10.1007/s10142-019-00686-z)
Supplement: Supplementary file 5 — (PDF 110 kb) [file 10142_2019_686_MOESM5_ESM.pdf]

Supplementary Figure S5: Nucleotide sequence alignment of α-gliadin genes from the B genomes of hexaploid wheat Chinese Spring and wild emmer wheat

```
Ta-alpha-B14      ATGAAGACCTTTCTCATCCTTGCCCTCCTTGCTATCGTGGC-----GACCACCACCACAA
Ta-alpha-B15      ATGAAGACCTTTCTCATCCTTGCCCTCCTTGCTATCATGGC-----GACCACCACCACAA
Ta-alpha-B16      ATGAAGACCTTTCTCATCCTTGCCCTCCTTGCTATCGTGGC-----GACCACCACCACAA
Ta-alpha-B17      ATGAAGACCTTTCTCATCCTTGCCCTCCTTGCTATCGTGGC-----GACCACCACCACAA
Ta-alpha-B18      ATGAAGACCTTTCTCATCCTTGCCCTCCTTGCTATCGTGGC-----GACCACCACCACAA
Ta-alpha-B11      ATGAAGACCTTTCTCATCCTTGCCCTCCTTGCTATCGTGGC-----GACCACCACCACAA
Ta-alpha-B12      ATGAAGACCTTTCTCATCCTTGCCCTCCTTGCTATCGTGGC-----GACCACCACCACAA
Ta-alpha-B13      ATGAAGACCTTTCTCATCCTTGCCCTCCTTGCTATCGTGGC-----GACCACCACCACAA
Ta-alpha-B23      ATGAAGTCATTTCTCATCCTTGCCCTCCTTGCTATCGTGGC-----GACCACCGCCACAA
Td-alpha-B15      ATGAAGTCCTTTCTCATCCTTGCCCTCCTTGCTATCGTGGC-----GACCACCGCCACAA
Td-alpha-B10      ATAAAGACCTTTCTCATCCTTGCCCTCCTTGCTATCGTGGT-----GACCACCGCCACTA
Ta-alpha-B21      ATGAACCTTCTCCTTGCCCTCGCCCTCCTTGCTATCGTGGC-----GACCACCGCCACAA
Td-alpha-B9       ---------------------GCCCTCCTTGCTATCGTGGC-----GACCACCGCCACAA
Ta-alpha-B20      ATGAAGACCTTTCTCATCCTTGCCCTCCTTGCTATCATGGCAATGCGACCACCGCCACAA
Td-alpha-B8       ATGAAGACCTTTCTCATCCTTGCCCTCCTTGCTATCGTGGC-----GACCACCGCCACAA
Td-alpha-B7       ATGAAGACCTTTCTCATCCTTGCCCTCCTTTCTATCGTGGC---GCGACCACCGCCACAA
Ta-alpha-B19      ATGAAGACCTTTCTCATCCTTGCCCTCCTTGCTATCGTGGCGATGCGACCACCGCCACAA
Ta-alpha-B1       ATGAAGACCTTTCTCATCTTTTCCCTCCTTGCTATCGTGGC-----GACCACTGCAACAA
Ta-alpha-B3       ATGAAGACCTTTCTCATCCTTTCCCTCCTTGCTATCGTGGC-----GACCACTGCCACAA
Td-alpha-B1       ATGAAGACCTTTCTCATCCTTTCCCTCCTTGCTATCGTGGC-----GACCACTGCCACAA
Td-alpha-B2       ATGAAGACCTTTCTCATCCTTTCCCTCCTTGCTATCGTGGC-----GACCACCGCCACAA
Td-alpha-B3       ATGAAGACCTTTCTCATCCTTTCCCTCCTTGCTATCGTGGC-----GACCACCGCCACAA
Td-alpha-B4       ATGAAGACCTTTCTCATCCTTTCCCTCCTTGCTATCGTGGC-----GACCACCGCGACAA
Ta-alpha-B2       ATGAAGACCTTTCTCATCTTTTCCCTCCTTGCTATCGTGGC-----GACCACTCCAACAA
Ta-alpha-B7       ATGAAGACCTTTCTCATCCTTGCCCTCCTTGCTATCGTGGC-----GACCACCGCCACAA
Ta-alpha-B10      ATGAAGACCTTTCTCATCCTTGCCCTCCTTGCTATCGTGGC-----GACCACCGCCACAA
Ta-alpha-B9       ATGAAGACCTTTCTCATCCTTGCCCTCCTTGCTATCGTGGC-----GACCACCGCCACAA
Ta-alpha-B6       ATGAAGACCTTTCTCATCCTTGCCCTC---------GTGGC-----GACCACCGCCACAA
Ta-alpha-B8       ATGAAGACCTTTCTCATCCTTGCCCTC---------GTGGC-----GACCACCGCCACAA
Td-alpha-B6       ATGAAGACCTTTCTCATCCTTGCCCTCCTTGCTATCGTGGC-----GACCACCGCCACAA
Td-alpha-B5       ATGAAGACCTTTCTCATCCTTGCCCTCCTTGCTATCGTGGT-----GACCACCGCCACAA
Td-alpha-B13      ATGAAGTCCTTTCTCATCCTTGCCCTCCTTGCTATCGTGGC-----GACCACCGCCACAA
Ta-alpha-B22      ATGAAGACCTTTCTCATCCTTGCCCTCCTTGCTATCGCGGT-----GACCACCGCCACTA

Ta-alpha-B14      CTGCAGTTAGAGTTCCAGTGCCACAATTGCAGCCACAAAATCCATCTCAGCAACAACCAC
Ta-alpha-B15      CTGCAGTTAGAGTTCCAGTGCCACAATTGCAGCCACAAAATCCATCTCAGCAACAACCAC
Ta-alpha-B16      CTGCAGTTAGAGTTCCAGTGCCACAATTGCAGCCACAAAATCCATCTCAGCAACAACCAC
Ta-alpha-B17      CTGCAGTTAGAGTTCCAGTGCCACAATTGCAGCCACAAAATCCATCTCAGCAACAACCAC
Ta-alpha-B18      CTGCAGTTAGAGTTCCAGTGCCACAATTGCAGCCACAAAATCCATCTCAGCAACAACCAC
Ta-alpha-B11      CTGCAGTTAGAGTTCCAGTGCCACAATTGCAGCCACAAAATCCATCTCAGCAACAACCAC
Ta-alpha-B12      CTGCAGTTAGAGTTCCAGTGCCATAATTGCAGCCACAAAATCCATCTCAGCAACAACCAC
Ta-alpha-B13      CTGCAGTTAGAGTTCCAGTGCCATAATTGCAGCCACAAAATCCATCTCAGCAACAACCAC
Ta-alpha-B23      CTGCAGTTAGAGTTCCAGTGCCACAATTGCAGCCACAAAATCCATCTCAGCAACAACCAC
Td-alpha-B15      CTGCAGTTAGAGTTCCAGTGCCACAATTGCAGCCACAAAATCCATCTCAGCAACAACCAC
Td-alpha-B10      CTGCAGTGAGAGTTCCAGTGCCATAATTGCAGCCACAAAATCCATCTCAGCAACAACCAC
Ta-alpha-B21      CTGCAGTTAGAGTTCCAGTGCCACAATTGCAGCCACAAAATCCATCTCAGCAACAACCAC
Td-alpha-B9       CTGCAGTTAGAGTTCCTGTGCCACAATTGCAGCCACAAAATCCATCTCAGCAACAACCAC
Ta-alpha-B20      CTGCAGTTAGAGTTCCAGTGCCACAATTGCAACCAGAAAATCCATCTCAGCAACAACCAC
Td-alpha-B8       CTGCAGTTAGAGTTCTAGTGCCACAATTGCAGCCACAAAATCCATCTCAGCAACAACCAC
Td-alpha-B7       CTGCAGTTAGAGTTCCAGTGCCACAATTGCAGCCACAAAATCCATCTCAGCAACAACCAC
Ta-alpha-B19      CTGCAGTTAGAGTTCCAGTGCCACAATTGCAGCCACAAAATCCATCTCAGCAACAACCAC
Ta-alpha-B1       CTGCAGTTAGAGTTCCAGTGCCACAATTGCAGCCGCAAAATCCATCTCAGCAACAACCAC
Ta-alpha-B3       CTGCAGTTAGATTTCCAGTGCCACAATTGCAGCCGCAAAATCCATCTCAGCAACAACCAC
Td-alpha-B1       CTGCAGTTAGAGTTCCAGTGCCACAATTGCAGCGGCAAAATCCATCTCAACAACAACCAC
```

```
Td-alpha-B2      CTTCAGTTAGAGTTCCAGTGCCACAATTGCAGCCGCAAAATCCATCTCAGCAACAACCAC
Td-alpha-B3      CTTCAGTTAGAGTTCCAGTGCCACAATTGCAGCCGCAAAATCCATCTCAGCAACAACCAC
Td-alpha-B4      CTGCAGTTAGAGATCCAGTGCCACAATTGCAGCCGCAAAATCCATCTCAGCAACAACCAC
Ta-alpha-B2      CTGCAGTTAGATTTCCAGTGCCACAATTGCAGCCGCAAAATCCATCTCAGCAACAACCAC
Ta-alpha-B7      CTGCAGTTAGAGTTCCAGTGCCACAATTGCAGCCACAAAATCCATCTCAGCAACAGCCAC
Ta-alpha-B10     CTGCAGTTAGAGTTCCAGTGCCACAATTGCAGCCACAAAATCCATCTCAGCAACAGCCAC
Ta-alpha-B9      CTGCAGTTAGAGTTCCAGTGCCACAATTGCAGCCACAAAATCCATCTCAGCAACAGCCAC
Ta-alpha-B6      CTGCAGTTAGAGTTCCAGTGCCACAATTGCAGCCACAAAATCCATCTCAGCAACAGCCAC
Ta-alpha-B8      CTGCAGTTAGAGTTCCAGTGCCACAATTGCAGCCAAAAAATCCATCTCAGCAACAGCCAC
Td-alpha-B6      CTGCAGTTAGAGTTCCAGTGCCACAATTGCAGCCACAAAATCCATCTCAGCAACAGCCAC
Td-alpha-B5      CTGCAGTTAGAGTTCCAGTGCCACAATTGCAGCCACAAAATCCATCTCAGCAACAGCCAC
Td-alpha-B13     CTGCAGTTAGAGTTCCAGTGCCACAATTGCAGCCACAAAATCCATCTCAGCAACAACCAC
Ta-alpha-B22     CTGCAGTGAGAGTTCCAGTGCCATAATTGCAGCCACAAAATCCATCTCAGCAACAACCAC

Ta-alpha-B14     AAGAGCAAGTTCCATTGGTGCAACAACAACAATTTCTAGGGCAGCAACAACAACAATTTC
Ta-alpha-B15     AAGAGCAAGTTCCATTGGTGCAACAACAACAATTTCTAGGGCAGCAACAACAACAATTTC
Ta-alpha-B16     AAGAGCAAGTTCCATTGGTGCAACAACAACAATTTCTAGGGCAGCAACAACAACAATTTC
Ta-alpha-B17     AAGAGCAAGTTCCATTGGTGCAACAACAACAATTTCTAGGGCAGCAACAACAACAATTTC
Ta-alpha-B18     AAGAGCAAGTTCCATTGGTGCAACAACAACAATTTCTAGGGCAGCAACAACAACAATTTC
Ta-alpha-B11     AAGAGCAAGTTCCATTGGTGCAACAACAACAATTTCTAGGGCAGCAACAACAACAATTTC
Ta-alpha-B12     AAGAGCAAGTTCCATTGGTGCAACAACAACAATTTCTAGGGCAGCAACAACAACAATTTC
Ta-alpha-B13     AAGAGCAAGTTCCATTGGTGCAACAACAACAATTTCTAGGGCAGCAACAACAACAATTTC
Ta-alpha-B23     AAGAGCAAGTTCCATTGGTGCAACAACAACAATTTCTAGGGCAGCAACAACAAAAATTTC
Td-alpha-B15     AAGAGCAAGTTCCATTGGTGCAACAACAACAATTTCTAGGGCAGCAACAACAAAAATTTC
Td-alpha-B10     AAGAGCAAGTTCCATTGGTGCAACAACAACAATTTCTAGGGCAGCAACAACAACACTTTC
Ta-alpha-B21     AAGAGCAAGTTCTATTGGTGCAACAACAACCATTTCTAGGGCAGCAACAACAACAATTTC
Td-alpha-B9      AAGAGCAAGTTCTATTGGTGCAACAACAACCATTTCTAGGGCAGCAACAACAACAATTTC
Ta-alpha-B20     AAGAGCAAGTTCCATTGGTGCAACAACAACAATTTCTAGGGCAGCAACAACAACAATTTC
Td-alpha-B8      AAGAGCAAGTTCCATTGGTGCAACAACAACAATTTCTAGGGCAGCAACAACAACAATTTC
Td-alpha-B7      AAGAGCAAGTTCCATTGGTGCAACAACAACAATTTCTAGGGCAGCAACAACAACAATTTC
Ta-alpha-B19     AAGAGCAAGTTCCATTGGTGCAACAACAACAATTTCTAGGGCAGCAA-------------
Ta-alpha-B1      AAGAGCAAGTTCCGTTGGTGCAACAACAACAATATCCAGGGCAGCAACAACCATTTCCAC
Ta-alpha-B3      AAGAGCAAGTTCCGTTGGTGCAACAACTACAATATCCAGGGCAACAACAACCATTTCCAC
Td-alpha-B1      AAGAGCAAGTTTCGTTGGTGCAACAACAACAATATCCAGGGCAGCAACAACCATTTCCAC
Td-alpha-B2      AAGAGCAAGTTCCGTTGGTGCAACAACAACAATATCCAGGGCAGCAACAACCATTTCCAC
Td-alpha-B3      AAGAGCAAGTTCCGTTGGTGCAACAACAACAATATCCAGGGCAGCAACAACCATTTCCAC
Td-alpha-B4      AAGAGCAAGTTCCGTTGGTGCAACAACAACAATATCCAGGGCAGCAACAACCATTTCCAC
Ta-alpha-B2      AAGAGCAAGTTCCGTTGGTGCAACAACTACAATATCCAAGGCAACAACAACCATTTCCAC
Ta-alpha-B7      AAGAGCAAGTTCCATTGGTACAACAACAACAATTTCCAGGGCAGCAACAACAATTTCCAC
Ta-alpha-B10     AAGAGCAAGTTCCATTGGTACAACAACAACAATTTCCAGGGCAGCAACAACAATTTCCAC
Ta-alpha-B9      AAGAGCAAGTTCCATTGGTACAACAACAACAATTTCCAGGGCAGCAACAACAATTTCCAC
Ta-alpha-B6      AAGAGCAAGTTCCATTGGTACAACAACAACAATTTCCAGGGCAGCAACAACAATTTCCAC
Ta-alpha-B8      AAGAGCAAGTTCCATTGGTACAACAACAACAATTTCCAGGGCAGCAACAACAATTTCCAC
Td-alpha-B6      AAGAGCAAGTTCCATTGGTACAACAACAACAATTTCCAGGGCAGCAACAACAATTTCCAC
Td-alpha-B5      AAGAGCAAGTTCCATTGGTACAACAACAACAATTTCCAGGGCAGCAACAACAATTTCCAC
Td-alpha-B13     AAGAGCAAGTTCCATTGGTGCAACAACAACAATTTCTAGGGCAGCAACAACAAAAATTTC
Ta-alpha-B22     AAGAGCAAGTTCCATTGGTGCAACAACAACAATTTCTAGGGCAGCAACAACAACACTTTC

Ta-alpha-B14     CAGG------------GCAACAACAACCATTTCCACCACAACAGCCATATC---------
Ta-alpha-B15     CAGG------------GCAACAACAACCATTTCCACCACAACAGCCATATC---------
Ta-alpha-B16     CAGG------------GCAACAACAACCATTTCCACCACAACAGCCATATC---------
Ta-alpha-B17     CAGG------------GCAACAACAACCATTTCCACCACAACAGCCATATC---------
Ta-alpha-B18     CAGG------------GCAACAACAACCATTTCCACCACAACAGCCATATC---------
Ta-alpha-B11     CAGG------------GCAACAACAACCATTTCCACCACAACAGCCATATC---------
Ta-alpha-B12     CAGG------------GCAACAACAACCATTTCCACCACAACAGCCATATC---------
Ta-alpha-B13     CAGG------------GCAACAACAACCATTTCCACCACAACAGCCATATC---------
Ta-alpha-B23     CAGG------------GCAGCAGCAACCATTTCCACCACAACAGCCATATC---------
Td-alpha-B15     CAGG------------GCAGCAGCAACCATTTCCACCACAACAGCCATATC---------
Td-alpha-B10     CAAG------------GCAGCAACAACCATTTCCACCACAACAGCCATATC---------
```

```
Ta-alpha-B21     CAGG------------GCAGCAATAACCATTTCGACCACAACAGCCATATC---------
Td-alpha-B9      CAGG------------GCAGCAATAACCATATCGACCACAACAGCCATATC---------
Ta-alpha-B20     CAGG------------GCAGCAATAACCATTTCGACCACAACAGCCATATC---------
Td-alpha-B8      CAGG------------GCAGCAATAACCATTTCGACCACAACAGCCATATC---------
Td-alpha-B7      CAGG------------GCAGCAATAACCATTTCGACCACAACAGCCATATC---------
Ta-alpha-B19     -----------------------TAACCATTTCGACCACAACAACCATATC---------
Ta-alpha-B1      CACAACAACCATATCCGCAGCCGCAACCATTTCCATCACAACAACCATTTC---------
Ta-alpha-B3      CACAACAGCCATATCCGCAGCCGCAACCATTTCCATCACAACAACCATTGC---------
Td-alpha-B1      CACAACAGCCATATCCGCAGCCGCAACCATTTCCATCACAACAACCATTTC---------
Td-alpha-B2      CACAACAGCCATATCCGCAGCCGCAACCATTTCCATCACAACAACCATTTC---------
Td-alpha-B3      CACAACAGCCATATCCGCAGCCGCAACCATTTCCATCACAACAACCATTTC---------
Td-alpha-B4      CACAACAGCCATATCCGCAGCCGCAA---TTTCCATCACAACAACCATTTC---------
Ta-alpha-B2      CACAACAGCCATATCCGCAGCCGCAACCATTTCCATCACAACAACCATTGC---------
Ta-alpha-B7      CACAACAGCCATATCCGCAGCCGCAACCATTTCCATCACAACAACCATATCTGCAACTGC
Ta-alpha-B10     CACAACAGCCATATCCGCAGCCGCAACCATTTCCATCACAACAACCATATCTGCAACTGC
Ta-alpha-B9      CACAACAGCCATATCCGCAGCCGCAACCATTTCCATCACAACAACCATATCTGCAACTGC
Ta-alpha-B6      CACAACAGCCATATCCGCAGCCGCAACCATTTCCATCACAACAACCATATCTGCAGCTGC
Ta-alpha-B8      CACAACAGCCATATCCGCAGCCGCAACCATTTCCATCACAACAACCATATCTGCAGCTGC
Td-alpha-B6      CACAACAGCCATATCCGCAACCGCAACCACTTCCATCACAACAACCATATCTGCAGCTGC
Td-alpha-B5      CACAACAGCCATATCCGCAGCCGCAACCATTTCCATCACAACAACCATATCTGCAGCTGC
Td-alpha-B13     CAGG------------GCAGCAGCAACCATTTCCACCACAACAGCCATATC---------
Ta-alpha-B22     CAGG------------GCAGCAACAACCATTTCCACCACAACAGCCATATC---------

Ta-alpha-B14     ---------CGCAGCCGCAACCATTTCTGCCACAACTACCATATCCGCAGCCGCAACCAT
Ta-alpha-B15     ---------CGCAGCCGCAACCATTTCTGCCACAACTACCATATCCGCAGCCGCAACCAT
Ta-alpha-B16     ---------CGCAGCCGCAACCATTTCTGCCACAACTACCATATCCGCAGCCACAACCAT
Ta-alpha-B17     ---------CGCAGCCGCAACCATTTCTGCCACAACTACCATATCCGCAGCCGCAACCAT
Ta-alpha-B18     ---------CGCAGCCGCAACCATTTCTGCCACAACTACCATATCCGCAGCCGCAACCAT
Ta-alpha-B11     ---------CGCAGCCGCAACCATTTCTGCCACAACTACCATATCCGCAGCCGCAACCAT
Ta-alpha-B12     ---------CGCAGCCGCAACCATTTCTGCCACAACTACCATATCCGCAGCCGCAACCAT
Ta-alpha-B13     ---------CGCAGCCGCAACCATTTCTGCCACAACTACCATATCCGCAGCCGCAACCAT
Ta-alpha-B23     ---------CGCAGCCGCAACCATTTCTGCCACAACTACCATTTCCGCAGCCGCAACCAT
Td-alpha-B15     ---------CGCAGCCGCAACCATTTCCGCCACAACTACCATTTCCGCAGCCGCAACCAT
Td-alpha-B10     ---------CGCAGCCGCAACCATTTCCACCACAACTACCATATCTGCAGCCGCAACCAT
Ta-alpha-B21     ---------CGCAGCCGCAACTATTTCCTCCAAAACTACCATATCCGCAGCCGCAACCAT
Td-alpha-B9      ---------CGCAGCCGCAACCATTTCCTCCAAAACTACCATATCCGCAGCCGCAACCAT
Ta-alpha-B20     ---------CGCAGCCGCAACCATTTCCTCCACAACTACCATGTCCGCAACCGCAACCAT
Td-alpha-B8      ---------CGCAGCCGCAACCATTTCCTCCACAACTACCATGTCCGCAGCCGCAACCAT
Td-alpha-B7      ---------CACAGCCGCAACCATTTCCTCCACAACTACCATGTCCGCAGCCGCAACCAT
Ta-alpha-B19     ---------CACAGCCGCAACCATTTCCTCCACAACTACCATGTCCGCAGCCGCAACCAT
Ta-alpha-B1      ---------TGCAGCCGCAACCATTTCTGCTACAACTACCATATCCGCAGCCACAACCAT
Ta-alpha-B3      ---------CGCAACCGCAACCATTTCTGCCACAACTACCATATCCGCAGCCACAACCAT
Td-alpha-B1      ---------CGCTGCCGCAACCATTTCTGCCACAACTACCATATGCGCAGCCACAACCAT
Td-alpha-B2      ---------CGCAGCCGCAACCATTTCTGCCACAACTACCATATCCGCAGCCACAACCAT
Td-alpha-B3      ---------CGCAGCCGCAACCATTTCTGCCACAACTACCATATCCGCAGCCACAACCAT
Td-alpha-B4      ---------CGCAGCCGCAACCATTTCTGCCACAACTACCATATCCGCAGCCACAACCAT
Ta-alpha-B2      ---------CGCAACGGCAACCATTTCTGCCACAACTACCATATCCGCAGCCACAACCAT
Ta-alpha-B7      AACCATTTCCGCAGCCGCAACCATTTCCGCCACAACTACCATATCCGCAGCCGCAATCAT
Ta-alpha-B10     AACCATTTCCGCAGCCGCAACCATTTCCGCCACAACTACCATATCCGCAGCCGCAATCAT
Ta-alpha-B9      AACCATTTCCGCAGCCGCAACCATTTCCGCCACAACTACCATATCCGCAGCCGCAATCAT
Ta-alpha-B6      AACCATTTCCGCAGCCGCAACCATTTCTGCCACAACTACCATATCCGCAGCCGCAATCAT
Ta-alpha-B8      AACCATTTCCGCAGCCGCAACCATTTCTGCCACAACTACCATATCCGCAGCCGCAATCAT
Td-alpha-B6      AACCATTTCCGCAGCCGCAACCATTTCCGCCACAACTACCATATCCGCAGCCGCAATCAT
Td-alpha-B5      AACCATTTCCGCAGCCGCAACCATTTCCGCCACAACTACCATATCCGCAGCCGCAATCAT
Td-alpha-B13     ---------CGCAGCCGCAACCATTTCCGCCACAACTACCATTTCCGCAGCCGCAACCAT
Ta-alpha-B22     ---------CGCAGCCGCAACCATTTCCACCACAACTACCATATCTGCAGCCGCAACCAT

Ta-alpha-B14     TTCCACCACAACAATCATATCCACAACCACAACCACAATATCCGCAACCACAACAAC---
Ta-alpha-B15     TTCCACCACAACAATCATATCCACAACCACAACCACAATATCCGCAACCACAACAAC---
```

```
Ta-alpha-B16      TTCCACCACAACAATCATATCCACAACCACAACCACAATATCCGCAACCACAACAAC---
Ta-alpha-B17      TTCCACCACAACAATCATATCCACAACCACAACCACAATATCCGCAACCACAACAAC---
Ta-alpha-B18      TTCCACCACAACAATCATATCCACAACCACAACCACAATATCCGCAACCACAACAAC---
Ta-alpha-B11      TTCCACCACAACAATCATATCCACAACCACAACCACAATATCCGCAACCACAACAAC---
Ta-alpha-B12      TTCCACCACAACAATCATATCCACAACCACAACCACAATATCCGCAACCACAACAAC---
Ta-alpha-B13      TTCCACCACAACAATCATATCCACAACCACAACCACAATATCCGCAACCACAACAAC---
Ta-alpha-B23      TTCCACCACAACAATCATATCCACAACCGCAACCACAGTATCCACAACCACAACAAC---
Td-alpha-B15      TTCCACCACAACAATCATATCCACAACCGCAACCACAGTATCCGCAACCACAACAAC---
Td-alpha-B10      TTCCACCACAACAATCATATCCACAACCACAACCACAATATCCGCAATCACAACAAC---
Ta-alpha-B21      TTACACCACATCAATCATATCCACAACCACAACCACAGTATCCGCAACCACAACAAG---
Td-alpha-B9       TTACACCACAGCAATCATATCCACAACCACAACCACAGTATCTGCAACCACAACAAG---
Ta-alpha-B20      TTCCACCACAACAATCATATCCACAACCACAACCACAGTATCCGCAACCACAACAAA---
Td-alpha-B8       TTCCACCACAACAATCATATCCACAACCACAACCACAGTATCCGCAACTACAACAAA---
Td-alpha-B7       TTCCACCACACCAATCATATCCACAACCACAACCACAGTATCCGCAACCACAACAAA---
Ta-alpha-B19      TTCCACCACAACAATCATATCCACAACCACAACCACAGTATCCGCAACCACAACAAA---
Ta-alpha-B1       TTCCACCACAACAACCATATCCACAACCGCAACCACAGTATCCGCAACCACAACAAC---
Ta-alpha-B3       TTCCACCACAACAACCATATCCACAACCGCAACCACAGTATCCGCAACCACAACAAC---
Td-alpha-B1       TTCCACCACAACAACCATATGCACAACCGCAACCACAGTATCCGCAACCACAACAAC---
Td-alpha-B2       TTCCACCACAACAACCATATCCACAACCGCAACCACAGTATCCGCAACCACAACAAC---
Td-alpha-B3       TTCCACCACAACAACCATATCCACAACCGCAACCACAGTATCCGCAACCACAACAAC---
Td-alpha-B4       TTCCACCACAACAACCATATCCACAACCGCAACCACAGTATCCGCAACCACAACAAC---
Ta-alpha-B2       TTCCACCACAACAACCATATCCACAACCGCAACCACAGTATCCACAACCACAACAAC---
Ta-alpha-B7       TTCCACCACAACAACCATATCCACAACAGCAACCACAGTATCTACAACCACAACAAC---
Ta-alpha-B10      TTCCACCACAACAACCATATCCACAACAGCAACCACAGTATCTACAACCACAACAAC---
Ta-alpha-B9       TTCCACCACAACAACCATATCCACAACAGCAACCACAGTATCTACAACCACAACAAC---
Ta-alpha-B6       TTCCACCACAACAACCATATCCACAACAGCGACCAAAGTATCTACAACCACAACAAC---
Ta-alpha-B8       TTCCACCACAACAACCATATCCACAACAGCGACCAAAGTATCTACAACCACAACAAC---
Td-alpha-B6       TTCCACCACAACAACCATATCCACAACAGCAACCACAGTATCTGCAACCACAACAAC---
Td-alpha-B5       TTCCACCACAACAACCATATCCACAACACCAACCACAGTATCTACAACCACAACAAC---
Td-alpha-B13      TTCCACCACAACAATCATATCCACAACCGCAACCACAGTATCCGCAACCACAACAAC---
Ta-alpha-B22      TTCCACCACAACAATCATATCCACAACCACAACCACAGTATCCGCAATCACAACAACAAC

Ta-alpha-B14      CAATTTCGCAGCAACAAGCACAACTAC---------AACAACAACAACAACAACAACAAC
Ta-alpha-B15      CAATTTCGCAGCAACAAGCACAACTAC---------AACAACAACAACAACAACAACAAC
Ta-alpha-B16      CAATTTCGCAGCAACAAGCACAACTAC---------AACAACAACAACAACAACAACAAC
Ta-alpha-B17      CAATTTCGCAGCAACAAGCACAACTACTACAACAACAACAACAACAACAACAACAACAAC
Ta-alpha-B18      CAATTTCGCAGCAACAAGCACAACTACTACAACAACAACAACAACAACAACAACAACAAC
Ta-alpha-B11      CAATTTCGCAGCAACAAGCACAACTAC---------AACAACAACAACAACAACAACAAC
Ta-alpha-B12      CAATTTCGCAGCAACAAGCACAACTACAACAACAACAACAACAACAACAACAACAACAAC
Ta-alpha-B13      CAATTTCGCAGCAACAAGCACAACTACAACAACAACAACAACAACAACAACAACAACAAC
Ta-alpha-B23      CAATTTCGCAGCAACAAGCACAACAACAACAACAACAACAACAACAA-------------
Td-alpha-B15      CAATTTCGCAGCAACAAGCACAACAACAACAACAACAACAACAACAACAACAACAACAAC
Td-alpha-B10      ---------------------AACAACAACAACAACAACAACAACAA-------------
Ta-alpha-B21      CAATTTCGCAGCAACAAGGACAAC------------AACAACAA---------CAACAAC
Td-alpha-B9       CAATTTCGCAGCAACAAGCACAAC------------AACAAAAA---------CAACAAC
Ta-alpha-B20      CAATTTCGCAGCAACAAGCACAAC------------AACAACAATGACAACCACAACAAC
Td-alpha-B8       CAATTTCGCAGCAACAAGCACAAC------------AACAACAATGACAACAACAACAAC
Td-alpha-B7       CAATTTCGCAGCAACAAGCACAAC------------AACAACAATGACAACAACAACAAC
Ta-alpha-B19      CAATTTCGCAGCAACAAGCACAAC------------AACAACAATGACAACAACAACAAC
Ta-alpha-B1       CAATTTCACAGCAACAAGCGCAACAAGCACAACAACAACAACAACAACAACAACAACAAT
Ta-alpha-B3       CAATTTCACAGCAACAAGCACAACAAGCACAACAACAACAACAACAACAACAACAACAAC
Td-alpha-B1       CAATTTCACTGCAACAAGCACAACAAGCACACCAACAACAACAACAACAACAACAACAAC
Td-alpha-B2       CAATTTCACTGCAACAAGCACAACAAGCACAACAACAACAACAACAACAACAACAACAAC
Td-alpha-B3       CAATTTCACTGCAACAAGCACAACAAGCACAACAACAACAACAACAACAACAACAACAAC
Td-alpha-B4       CAATTTCACA------------------ACAACAACAACAACAACAACAACAACAACAAC
Ta-alpha-B2       CAATTTCACAGCAACAAGCACAATAAGCACAACAACAACAACAACAACAACAACAACAAC
Ta-alpha-B7       CAATTTCGCAGCAACAAGCACAACAACAACAACAACAACAACAACAACAACAACAACAAC
Ta-alpha-B10      CAATTTCGCAGCAACAAGCACAACAACAACAACAACAACAACAACAATAACAACAACAAC
Ta-alpha-B9       CAATTTCGCAGCAACAAGCACAACAACAACAACAACAACAACAACAACAACAACAACAAC
```

```
Ta-alpha-B6       CAATTTCGCAGCAACAAGCACAACAACAACAACAACAACAACAACAACAACAACAACAAC
Ta-alpha-B8       CAATTTCGCAGCAACAAGCACAACAACAACAACAACAACAACAACAACAACAACAACAAC
Td-alpha-B6       CAATTTCGCAGCAACAAGCACAACAACAACAACAA-------------------------
Td-alpha-B5       CAATTTCGCAGCAACAAGCACAACAACAACAACAACAACAACAACAACAACAACAACAAC
Td-alpha-B13      CAATTTCGCAGCAACAAGCACAAC------------------------------------
Ta-alpha-B22      CAATTTCACAGCAACAACAACAACAACAACAACAACAACAACAACAACAACAACAACAAC

Ta-alpha-B14      AACAACAACAACAA----------------------------------------------
Ta-alpha-B15      AACAACAA----------------------------------------------------
Ta-alpha-B16      AACAACAA----------------------------------------------------
Ta-alpha-B17      AACAACAACAACAA----------------------------------------------
Ta-alpha-B18      AACAACAACAACAA----------------------------------------------
Ta-alpha-B11      AACAACAACAACAA----------------------------------------------
Ta-alpha-B12      AACAACAACAACAACAACAA----------------------------------------
Ta-alpha-B13      AACAA-------------------------------------------------------
Ta-alpha-B23      ------------------------------------------------------------
Td-alpha-B15      AACAACAACAACAACAACAACAACAA----------------------------------
Td-alpha-B10      ------------------------------------------------------------
Ta-alpha-B21      AA----------------------------------------------------------
Td-alpha-B9       AA----------------------------------------------------------
Ta-alpha-B20      AA----------------------------------------------------------
Td-alpha-B8       AA----------------------------------------------------------
Td-alpha-B7       AA----------------------------------------------------------
Ta-alpha-B19      AA----------------------------------------------------------
Ta-alpha-B1       AACAATAACAACAACAACAA----------------------------------------
Ta-alpha-B3       AACAACAACAACAACAACAA----------------------------------------
Td-alpha-B1       AACAACAACAACAACAACAACAACAACAACAACAACAACAACAACAACAACAACAA----
Td-alpha-B2       AACAACAACAACAACAACAACAACAACAACAACAACAACAACAACAACAACAACAACAAC
Td-alpha-B3       AACAACAACAACAACAACAACAACAACAACAACAACAACAACAACAACAA----------
Td-alpha-B4       AACAACAACAACAACAACAACAACAACAACAACAACAACAACAACAACAA----------
Ta-alpha-B2       AACAACAACAA-------------------------------------------------
Ta-alpha-B7       AACAA-------------------------------------------------------
Ta-alpha-B10      AACAA-------------------------------------------------------
Ta-alpha-B9       AACAA-------------------------------------------------------
Ta-alpha-B6       AACAACAACAACAACAA-------------------------------------------
Ta-alpha-B8       AACAACAACAACAACAACAA----------------------------------------
Td-alpha-B6       ------------------------------------------------------------
Td-alpha-B5       AACAA-------------------------------------------------------
Td-alpha-B13      ------------------------------------------------------------
Ta-alpha-B22      AACAA-------------------------------------------------------

Ta-alpha-B14      --------------------------ATCCTTCAACAAATTCTGCAACAACAAC---TGA
Ta-alpha-B15      --------------------------ATCCTTCAACAAATTCTGCAACAACAAC---TGA
Ta-alpha-B16      --------------------------ATCCTTCAACAAATTCTGCAACAACAAC---TGA
Ta-alpha-B17      --------------------------ATCCTTCAACAAATTCTGCAACAACAAC---TGA
Ta-alpha-B18      --------------------------ATCCTTCAACAAATTCTGCAACAACAAC---TGA
Ta-alpha-B11      --------------------------ATCCTTCAACAAATTCTGCAACAACAAC---TGA
Ta-alpha-B12      --------------------------ATCCTTCAACAAATTCTGCAACAACAAC---TGA
Ta-alpha-B13      --------------------------ATCCTTCAACAAATTCTGCAACAACAAC---TGA
Ta-alpha-B23      --------------------------ATCCTTCAACAAATTCTGCAACAACAAC---TGA
Td-alpha-B15      --------------------------ATCCTTCAACAAATTCTGCAACAACAAC---TGA
Td-alpha-B10      --------------------------ATCCTTCAACAAATTCTGCAACAACAAC---TGA
Ta-alpha-B21      --------------------------ATCCTTCAACAAATTCTGCAACAACAAC---TGA
Td-alpha-B9       --------------------------ATCCTTCAACAAATTCTGCAACAACAAC---TGA
Ta-alpha-B20      --------------------------ATCCTTCAACAAATTCTACAAAAACAAC---TGA
Td-alpha-B8       --------------------------ATCATTCAACAAATTCTACAACAACAAC---TGA
Td-alpha-B7       --------------------------ATCCTTCAACAAATTCTACAACAACAAC---TGA
Ta-alpha-B19      --------------------------ATCCTTCAACAAATTCTACAACAACAAC---TGA
Ta-alpha-B1       --------------------------ATCCTTCAACAAATTCTGCAACAACAACAACTGA
```

```
Ta-alpha-B3       --------------------------ATCCTTCAACAAATTCTGCAACAACAACAACTGA
Td-alpha-B1       --------------ATCCTTCAACAAATTCTTCAACAAATTCTGCAACAACAACAACTGA
Td-alpha-B2       AACAACAACAACAAATCCTTCAACAAATTCTTCAACAACTTTTGCAACAACAACAACTGA
Td-alpha-B3       --------------ATCCTTCAACAAATTCTTCAACAACTTTTGCAACAACAACAACTGA
Td-alpha-B4       --------------------CAACAAATCCTTCAACAAATTCTACAACAACAACAACTGA
Ta-alpha-B2       --------------------------ATCCTTCAACAAATTCTGCAACAACAACAACTAA
Ta-alpha-B7       --------------------------ATCCTTCAACAAATTTTGCAACAACAAC---TGA
Ta-alpha-B10      --------------------------ATCCTTCAACAAATTTTGCAACAACAAC---TGA
Ta-alpha-B9       --------------------------ATCCTTCAACAAATTTTGCAACAACAAC---TGA
Ta-alpha-B6       --------------------------ATCCTTCAACAAATTTTGCAACAACAAC---TGA
Ta-alpha-B8       --------------------------ATCCTTCAACAAATTTTGCAACAACAAC---TGA
Td-alpha-B6       --------------------------ATCCTTCAACAAATTTTGCAACAACAAC---TGA
Td-alpha-B5       --------------------------ATCCTTCAACAAATTTTGCAACAACAAC---TGA
Td-alpha-B13      ------------------------------------------------------------
Ta-alpha-B22      --------------------------ATCCTTCAACAAATTCTGCAACAACAAT---TGA

Ta-alpha-B14      TTCCATGCAGGGATGTCGTCTTGCAACAACCCAATATAGCACATGCAAGCTCAAAAGTAT
Ta-alpha-B15      TTCCATGCAGGGATGTCGTCTTGCAACAACCCAATATAGCACATGCAAGCTCACAAGTAT
Ta-alpha-B16      TTCCATGCAGGGATGTCGTCTTGCAACAACCCAATATAGCACATGCAAGCTCACAAGTAT
Ta-alpha-B17      TTCCATGCAGGGATGTCGTCTTGCAACAACCCAATATAGCACATGCAAGCTCACAAGTAT
Ta-alpha-B18      TTCCATGCAGGGATGTCGTCTTGCAACAACCCAATATAGCACATGCAAGCTCACAAGTAT
Ta-alpha-B11      TTCCATGCAGGGATGTCGTCTTGCAACAACCCAATATAGCACATGCAAGCTCAAAAGTAT
Ta-alpha-B12      TTCCATGYAGGGATGTCGTCTTGCAACAACCCAACATAGCACATGCAAGCTCACAAGTAT
Ta-alpha-B13      TTCCATGTAGGGATGTCGTCTTGCAACAACCCAACATAGCACATGCAAGCTCACAAGTAT
Ta-alpha-B23      TTCCATGCAAGGATGTCGTCTTGCAACAACCCAACATAGCACATGCTAGCTCACAAGTAT
Td-alpha-B15      TTCCATGCAGGGATGTCGTCTTGCAACAACCCAACATAGCACATGCTAGCTCACAAGTAT
Td-alpha-B10      TTCCATGCAGGGATGTCGTCTTGCAACAACCCAACATAGCACATGCTAGCTCACAAGTAT
Ta-alpha-B21      TTCCATGCAGGGATGTCGTCTTGCAACAACCCAACATAGCACATGCAAGCTCACAAGTAT
Td-alpha-B9       TTCCATGCAGGGATGTCGTCTTGCAACAACCCAACATAGCACATGCAAGCTCACAAGTAT
Ta-alpha-B20      TTCCATGCAGGGATGTCGTCTTGCAACAACCCAACATAGCACATGCAAGCTCACAAGTAT
Td-alpha-B8       TTCCATGCAGGGATGTCGTCTTGCAACAACCCAACATAGCACATGCAAGCTCACAAGTAT
Td-alpha-B7       TTCCATGCAGGGATGTCGTCTTGCAACAACCC----------------------------
Ta-alpha-B19      TTCCATGCAGGGATGTCGTGTTGCAACAACCC----------------------------
Ta-alpha-B1       TTCCATGCAGGGATGTCGTCTTGCAACAACACAACATAGCGCATGCAAGCTCACAAGTAT
Ta-alpha-B3       TTCCATGCAGGGATGTCGTCTTGCAACAACACAACATAGCGCATGCAAGCTCACAAGTAT
Td-alpha-B1       TTCCATGCAGGGATGTCGTCTTGCAACAACACAACATAGCACATGCAAGCTCACAAGTAT
Td-alpha-B2       TTCCATGCAGGGATGTGGTCTTGCAACAACACAACATAGCGCATGCAAGCTCACAAGTAT
Td-alpha-B3       TTCCATGCAGGGATGTGGTCTTGCAACAACACAACATAGCGCATGCAAGCTCACAAGTAT
Td-alpha-B4       TTCCATGCAGGGATGTCGTCTTGCAACAACACAACATAGCGCATGCAAGCTCACAACTAT
Ta-alpha-B2       TTCCATGCAGGGATGTCGTCTTGCAACAACACAACATAGCGCATGCAAGCTCACAAGTAT
Ta-alpha-B7       TTCCATGCAGGGATGTTGTCTTGCAACAACACAACATAGCGCATGCAAGCTCACAAGTTT
Ta-alpha-B10      TTCCATGCAGGGATGTTGTCTTGCAACAACACAACATAGCGCATGCAAGCTCACAAGTTT
Ta-alpha-B9       TTCCATGCAGGGATGTTGTCTTGCAACAACACAACATAGCGCATGCAAGCTCACAAGTTT
Ta-alpha-B6       TTCCATGCAGGGATGTTATCTTGCAACAACACAACATAGCGCATGCAAGCTCACAAGTTT
Ta-alpha-B8       TTCCATGCAGGGATGTTGTCTTGCAACAACACAACATAGCGCATGCAAGCTCACAAGTTT
Td-alpha-B6       TTCCATGCAGGGATGTTGTCTTGCAACAACACAACATAGAGCATGCAAGCTCACAAGTTT
Td-alpha-B5       TTCCATGCAGGGATGTTGTCTTGCAACAACACAACATAGCGCATGCAAGCTCACAAGTTT
Td-alpha-B13      ------------------------------------------------------------
Ta-alpha-B22      TTCCATGCAGGGATGTCATCTTGCAACAACCCAACATAGCACATGCAAGCTCACAAGTAT

Ta-alpha-B14      CGCAACAAAGT---TACCAACTGTTGCAACAATTATGTTGTCAGCAACTGTGGCAGACCC
Ta-alpha-B15      CGCAACAAAGT---TACCAACTGTTGCAACAATTATGTTGTCAGCAACTGTGGCAGACCC
Ta-alpha-B16      CGCAACAAAGT---TACCAACTGTTGCAACAATTATGTTGTCAGCAACTGTGGCAGACCC
Ta-alpha-B17      CGCAACAAAGT---TACCAACTGTTGCAACAATTATGTTGTCAGCAACTGTGGCAGACCC
Ta-alpha-B18      CGCAACAAAGT---TACCAACTGTTGCAACAATTATGTTGTCAGCAACTGTGGCAGACCC
Ta-alpha-B11      CGCAACAAAGT---TACCAACTGTTGCAACAATTATGTTGTCTGCAACTGTGGCAGACCC
Ta-alpha-B12      YGCAACAAAGT---TACCAACTGTTGCAACAATTATGTTGTCAGCAACTGTGGCAGACCC
Ta-alpha-B13      CGCAACAAAGT---TACCAACTGTTGCAACAATTATGTTGTCAGCAACTGTGGCAGACCC
Ta-alpha-B23      CGCAACAAAGT---TACCAACTGTTGCAACAATTATGTTGTCAGCAATTGTGGCAGACCC
```

```
Td-alpha-B15      CGCAACAAAGT---TACCAACTGTTGCAACAATTATGTTGTCAGCAATTGTGGCAGACCC
Td-alpha-B10      CGCAACAAAGT---TACCAACTGTTGCAACAATTATGTTGTCAGCAATTGTGGCAGACCC
Ta-alpha-B21      CTCAACAAAGT---TACCATCTATTGCAACAATTATGTTGTCAGCAACTGTGGCAGACCC
Td-alpha-B9       CTCAACAAAGT---TACCATCTATTGCAATAATTATGTTGTCAGCAACTGTGGCAGACCC
Ta-alpha-B20      CACAACAAAGT---TACCATCTGTTGCAACAATTATGTTGTTAGCAACTGTGGCAGACCC
Td-alpha-B8       CGCAACAAAGT---TACCATCTGTTGCAACAATTATGTTGTCAGCAACTGTGGCAGACCC
Td-alpha-B7       ------------------------------------------------------------
Ta-alpha-B19      ------------------------------------------------------------
Ta-alpha-B1       TGCAACAAAGTAGTTACCAACTGTTGCAACAATTATGTTGTCAACGGTTGTGGCAGATCC
Ta-alpha-B3       TGCAACAAAGTAGTTACCAACTGTTGCAACAATTATGTTGTCAACGTTTGTGGCAGATCC
Td-alpha-B1       TGCAACAAAGTAGTTACCAACTGTTGCAACAATTATGTTGTCAACGGTTGTGGCAGATCC
Td-alpha-B2       TGCAACAAAGTAGTTACCAACTGTTGCAACAATTATGTTGTCAACGGTTGTGGCAGATCC
Td-alpha-B3       TGCAACAAAGTAGTTACCAACTGTTGCAACAATTATGTTGTCAACGGTTGTGGCAGATCC
Td-alpha-B4       TGCAACAAAGTAGTTACCAACTATTGCAACAATTATGTTGTCAACGGTTGTGGCATATCC
Ta-alpha-B2       TGCAACAAAGTAGTTACCAACTGTTGCAACAATTATGTTGTCAACGGTTGTGGCAGATCC
Ta-alpha-B7       TGCAACAAAGTACTTACCAGCTATTGCAACAATTGTGTTGTCAACAACTGTTGCAGATCC
Ta-alpha-B10      TGCAACAAAGTACTTACCAGCTATTGCAACAATTGTGTTGTCAACAACTGTTGCAGATCC
Ta-alpha-B9       TGCAACAAAGTACTTACCAGCTATTGCAACAATTGTGTTGTCAACAACTGTTGCAGATCC
Ta-alpha-B6       TGCAACAAAGTACTTACCAGCTATTGCAACAATTGTGTTGTCAACAACTGTTGCAGATCC
Ta-alpha-B8       TGCAACAAAGTACTTACCAGCTATTGCAACAATTGTGTTGTCAACAACTGTTGCAGATCC
Td-alpha-B6       TGCAACAAAGTTCTTACCAGCTATTGCAACAATTATGTTGTCAACAATTGTTGCAGATCC
Td-alpha-B5       TGCAACAAAGTACTTACCAGCTATTGCAACAATTGTGTTGTCAACAACTGTTGCAGATCC
Td-alpha-B13      ------------------------------------------------------------
Ta-alpha-B22      CGCAACAAAGT---TACCAACTTTTGCAACAATTATGTTGTCAGCAACTGTGGCAGACCC

Ta-alpha-B14      CCGAGCAGTCACGGTGCCAAGCCATCCACAATGTCATTCATGCTATTATTTTGCATCAAC
Ta-alpha-B15      CCGAGCAGTCACGGTGCCAAGCCATCCACAATGTCATTCATGCTATTATTTTGCATCAAC
Ta-alpha-B16      CCGAGCAGTCACGGTGCCAAGCCATCCACAATGTCATTCATGCTATTATTTTGCATCAAC
Ta-alpha-B17      CCGAGCAGTCACGGTGCCAAGCCATCCACAATGTCATTCATGCTATTATTTTGCATCAAC
Ta-alpha-B18      CCGAGCAGTCACGGTGCCAAGCCATCCACAATGTCATTCATGCTATTATTTTGCATCATC
Ta-alpha-B11      CCGAGCAGTCACGGTGCCAAGCCATCCACAATGTCATTCATGCTATTATTTTGCATCATC
Ta-alpha-B12      CCGAGCAGTCACGGTGCCAAGCCATCCACAATGTCATTCATGCTATTATTTTGCATCAAC
Ta-alpha-B13      CCGAGCAGTCACGGTGCCAAGCCATCCACAATGTCATTCATGCTATTATTTTGCATCAAC
Ta-alpha-B23      CCGAACAGTCACGGTGCCAAGCCATCCACAATGTCGTGCATGCTATTATTCTGCATCATC
Td-alpha-B15      CCGAGCAGTCACGGTGCCAAGCCATCCACAATGTCGTGCATGCTATTATTCTGCATCA--
Td-alpha-B10      CCGAGCAGTCACGGTGCCAAGCCATCCACAATGTCGTGCATGCTATTATTCTGCATCA--
Ta-alpha-B21      CCGAGCTGTCACAGTGCCAAGTCATCCACAATGTCGTTCATGCTATTATTCTGCATCATC
Td-alpha-B9       CCGAGCTGTCATGGTGCCAAGTCATCCACAATGTCGTTCATGCTATTATTCTGCATCATT
Ta-alpha-B20      CCGAGCTGTCACGGTGCCAAGTCATCCACAATGTCGTTCATGCTATTATTCTCCATCAAC
Td-alpha-B8       CCGAGCTGTCACGGTGCCAAGTCATCCACAATGTCGTTCATGCTATTATTCTCCATCAAC
Td-alpha-B7       -CGAGCTGTCACGGTGCCAAGTCATCCACAATGTCGTTCATGCTATTATTCTCCATCAAC
Ta-alpha-B19      -CGAGCTGTCACGGTGCCAAGTCATCCACAATGTCGTTCATGCTATTATTCTCCATCAAC
Ta-alpha-B1       CCGAGCAGTCGCGGTGCCAAGCCATCCACAATGTCGTTCATGCTATTATTCTGCAACAAC
Ta-alpha-B3       CCGAGCAGTCGCGGTGCCAAGCCATCCACAATGTCGTTCATGCTATTATTCTGCAACAAC
Td-alpha-B1       CCGAGAAGTCGCGGTGCCAAGCCATCCACAATGTCGTTCATGCTATTATTCTGCAACAAC
Td-alpha-B2       CCGAGCAGTCGCGGTGCCAAGCCATCCACAATGTCGTTCATGCTATTATTCTGCAACAAC
Td-alpha-B3       CCGAGCAGTCGCGGTGCCAAGCCATCCACAATGTCGTTCATGCTATTATTCTGCAACAAC
Td-alpha-B4       CCGAGCAGTCGCGGTGCCATGCCATCCACAATGTCGTTCATGCTATTATTCTACAACAAC
Ta-alpha-B2       CCGAGCAGTCGCGGTGCCAAGCCATCCACAATGTTGTTCATGCTATTATTCTGCAACAAC
Ta-alpha-B7       CTGAGCAGTCGAGGTGCCAAGCCATCCATAATGTTGCTCATGCTATTATTATGCATCAAC
Ta-alpha-B10      CTGAGCAGTCGAGGTGCCAAGCCATCCATAATGTTGCTCATGCTATTATTATGCATCAAC
Ta-alpha-B9       CTGAGCAGTCGAGGTGCCAAGCCATCCATAATGTTGCTCATGCTATTATTATGCATCAAC
Ta-alpha-B6       CTGAGCAGTCGAGGTGCCAAGCCATACATAATGTTGTTCATGCTATTATTATGCATCAAC
Ta-alpha-B8       CTGAGCAGTCGAGGTGCCAAGCCATCCATAATGTTGTTCATGCTATTATTATGCATCAAC
Td-alpha-B6       CTGAGCAGTCGAGGTGCCAAGCCATCCATAATGTTGCTCATGCTATTATTATGCATCAAC
Td-alpha-B5       CTGAGCAGTTGAGGTGCCAAGCCATCCATAATGTTGCTCATGCTATTATTCTGCATCATC
Td-alpha-B13      ------------------------------------------------------------
Ta-alpha-B22      CTGAGCAGTCACGGTGCCAAGCCATCCACAATGTCGTTCATGCTATTATTCTGCATCAAC
```

```
Ta-alpha-B14      AACAACAACAACAACAACAACAACAACAACAACAACAACAACAAC---------------
Ta-alpha-B15      AACAACAACAACAACAACAACAACAACAACAACAACAACAACAACAAC------------
Ta-alpha-B16      AACAACAACAACAACAACAACAACAACAACAACAACAACAACAACAACAACAACAACAAC
Ta-alpha-B17      AACAACAACAACAACAACAACAACAACAACAACAACAACAACAACAACAAC---------
Ta-alpha-B18      GTCATCAACAACAACAACAACAACAACAACAACAACAACAACAAC---------------
Ta-alpha-B11      AACAACAACAACAACAACAACAACAACAACAACAACAACAACAACAAC------------
Ta-alpha-B12      AACAACAACAACAACAACAACAACAACAACAACAACAACAACAACAACAAC---------
Ta-alpha-B13      AACAACAACAACAACAACAACAACAACAACAACAACAACAACAACAACAAC---------
Ta-alpha-B23      AACAACAACAACAACAACAACAACAACAACAACAACAACAACAACAACAACAACAACAAC
Td-alpha-B15      -------------------------------ACAACAACAACAACAACAACAACAACAAC
Td-alpha-B10      ----ACAAAAACAACAACAACAACAACAACAACAACAACAACAACAACAACAACAACAAC
Ta-alpha-B21      AATAACAACAAAAACAACAACAACAAC---------------------------------
Td-alpha-B9       AACAACAACAAAAACAACAACAACAAC---------------------------------
Ta-alpha-B20      AACAACAACAAAAACAACAACAACAACAACAACAACAAC---------------------
Td-alpha-B8       AACAACAACAAAAACAACAAAAACAACAACAACAAC------------------------
Td-alpha-B7       AACAACAACAAAAACAACAACAACAACAACAAC---------------------------
Ta-alpha-B19      AACAACAACAAAAACAACAACAACAACAACAAC---------------------------
Ta-alpha-B1       AACAACAACAACAACAACAACAACAACAACAACAACAACAACAAC---------------
Ta-alpha-B3       AACAACAACAACAACAACAACAACAACAACAACAACAACAACAACAACAACAACAACAAC
Td-alpha-B1       AACAGCAACGACAACAACAACAACAACAACAAC---------------------------
Td-alpha-B2       AACAACAACAACAACAACAACAACAACAACAACAACAACAACAACAACAACAACAACAAC
Td-alpha-B3       AACAACAACAACAACAACAACAACAACAACAACAACAACAACAACAACAACAACAACAAC
Td-alpha-B4       AACAACAACAACAACAACAACAACAACAACAACAACAACAACAACAACAACAACAACAAC
Ta-alpha-B2       AACAACAACAACAACAACAACAACAACAAC------------------------------
Ta-alpha-B7       AACAACAACAACAACAAG---AACAACAACAACAGTTGCAACAACAACAACAGCAGCAAC
Ta-alpha-B10      AACAACAACAACAACAAG---AACAACAACAACAGTTGCAACAACAACAGCAGCAGCAAC
Ta-alpha-B9       AACAACAACAACAACAAG---AACAACAACAACAGTTGCAACAACAACAACAGCAGCAAC
Ta-alpha-B6       AAGAACAACAACAACAGTTGCAACAACAACAACAACAGCAACTGCAACAACAACAACAAC
Ta-alpha-B8       AAGAACAACAACAACAGTTGCAACAACAACAACAACAGCAACTGCAACAACAACAACAAC
Td-alpha-B6       AACAACAACAACAACAACAAGAACAACAACAACAGTTGCAGCAACAACAACAACAGCAGC
Td-alpha-B5       AACAACAATAACAACA------ACAACAACAACAGCAGCAACAGCAACAACAACAACAAC
Td-alpha-B13      ---AACAACAACAACAACAACAACAACAACAA----------------------------
Ta-alpha-B22      AACAACAACAACAACAACAACAACAACAACAACAACAACAACAACAACAACAACAACAAC

Ta-alpha-B14      ------------------------------------------------------------
Ta-alpha-B15      ------------------------------------------------------------
Ta-alpha-B16      AACAAC------------------------------------------------------
Ta-alpha-B17      ------------------------------------------------------------
Ta-alpha-B18      ------------------------------------------------------------
Ta-alpha-B11      ------------------------------------------------------------
Ta-alpha-B12      ------------------------------------------------------------
Ta-alpha-B13      ------------------------------------------------------------
Ta-alpha-B23      AACAAC------------------------------------------------------
Td-alpha-B15      AACAAC------------------------------------------------------
Td-alpha-B10      AACAAC------------------------------------------------------
Ta-alpha-B21      ------------------------------------------------------------
Td-alpha-B9       ------------------------------------------------------------
Ta-alpha-B20      ------------------------------------------------------------
Td-alpha-B8       ------------------------------------------------------------
Td-alpha-B7       ------------------------------------------------------------
Ta-alpha-B19      ------------------------------------------------------------
Ta-alpha-B1       ------------------------------------------------------------
Ta-alpha-B3       AACAACAACAACAACAACAACAACAACAACAACAACAACAACAACAACAACAACAACAAC
Td-alpha-B1       ------------------------------------------------------------
Td-alpha-B2       AACAAC------------------------------------------------------
Td-alpha-B3       AACAACAACAACAACAACAACAACAACAACAACAACAACAACAACAACAACAGCAAC---
Td-alpha-B4       AACAACAACAACAACAACAACAACAACAAC------------------------------
```

```
Ta-alpha-B2       ------------------------------------------------------------
Ta-alpha-B7       TGCAACAACAACGACAACAAC---------------------------------------
Ta-alpha-B10      TGCAACAACAATGACAACAAC---------------------------------------
Ta-alpha-B9       TGCATCAACAACGACAACAAC---------------------------------------
Ta-alpha-B6       AACAACAACAACAACAACAACAACAACAAC------------------------------
Ta-alpha-B8       AACAACAACAACAACAACAAC---------------------------------------
Td-alpha-B6       AACTGCAACAACAACAACAACAAC------------------------------------
Td-alpha-B5       AACAACAAC---------------------------------------------------
Td-alpha-B13      ------------------------------------------------------------
Ta-alpha-B22      AACAACAACAACAACAACGACGACAACAACAACAACGACAACAACAACAACGACAACAAC


Ta-alpha-B14      ---------CGTCGAGCCAGGTCTCCTACCAGCAGCCTCAGCAACAATATCCATCAGGCC
Ta-alpha-B15      ---------CGTCGAGCCAGGTCTCCTACCAGCAGCCTCAGCAACAATATCCATCAGGCC
Ta-alpha-B16      ---------CGTCGAGCCAGGTCTCCTACCAGCAGCCTCAGCAACAATATCCATCAGGCC
Ta-alpha-B17      ---------CGTCGAGCCAGGTCTCCTACCAGCAGCCTCAGCAACAATATCCATCAGGCC
Ta-alpha-B18      ---------CGTCGAGCCAGGTCTCCTACCAGCAGCCTCAGCAACAATATCCATCAGGCC
Ta-alpha-B11      ---------CGTCGAGCCAGGTCTCCTACCAGCAGCCTCAGCAACAATATCCATCAGGCC
Ta-alpha-B12      ---------CGTCGAGCCAGGTCTCCTACCAGCAGCCTCAGCAACAATATCCATCAGGCC
Ta-alpha-B13      ---------CGTCGAGCCAGGTCTCCTACCAGCAGCCTCAGCAACAATATCCATCAGGCC
Ta-alpha-B23      ---------CGTCGAGCCAGGTCTCCTACCAGCAGCCTCAACAATAATATCCATCGGCCC
Td-alpha-B15      ---------CGTCGAGCCAGGTCTCCTACCAGCAGCCTCAACAACAATATCCATCGGCCC
Td-alpha-B10      ---------CGTCGAGCCAGGTCTCCTACCAGCAGCCTCAACAACAATATACATCGGCCC
Ta-alpha-B21      ---------CGTCGAGCCAGGTCTTCTACCAGCAGCCTCAGCAACAATATCCATCAGGCC
Td-alpha-B9       ---------CGTCGAGCCAGGTCTTCTACCAGCAGCCTCAGCAACAATATCCATCAGGCC
Ta-alpha-B20      ---------CGTCGAGCCAGGTCTTCTAGCAGCAACCTCAGCAACAATATCCATCAGCCC
Td-alpha-B8       ---------CGTCGAGCCAGGTCTTCTAGCAGCAGCCTCAGCAACAATATCCATCAGGCC
Td-alpha-B7       ---------CATCGAGCCAGGTCTTCTACCAGCAGCCTCAGCAATAATATCCATCAGGCC
Ta-alpha-B19      ---------CGTCGAGCCAGGTCTTCTACCAGCAGCCTCAGCAACAATATCCATCAGGCC
Ta-alpha-B1       ---------CGTCGAGCCAGGTCTCCTACCAGCAGCCTCAGCAACAATATCCATCGGGCC
Ta-alpha-B3       AACAACAACCGTCGAGCCAGGTCTCCTACCAGCAGCCTCAGCAACAATATCCATCGGGCC
Td-alpha-B1       ---------CGTCGAGCCAGGTCTCCTACCAGCAGCCTCAGCAACAATATCCATCGGGCC
Td-alpha-B2       ---------CGTCGAGCCAGGTCTCCTACCAGCAGCCTCAGCAACAATATCCATCGGGCC
Td-alpha-B3       ---------CGTCGAGCCAGATCTCCCTCTAGCAGCCTCAACAACAATATCCATCAGGCC
Td-alpha-B4       ---------CGTCGAGCCAGATCTCCCTCCAGCAGCCTCAACAACAATATCCATCAGGCC
Ta-alpha-B2       ---------CGTCGAGCCAGGTCTCCTACCAGCAGCCTCAGCAACAATATCCATCGGGCC
Ta-alpha-B7       ---------CGTCGAGCCAGGTCTCCTTCCAACAGCCTCAGCAGCAATATCCATCAAGCC
Ta-alpha-B10      ---------CGTCGAGCCAGGTCTCCTTCCAACAGCCTCAGCAGCAATATCCATCAAGCC
Ta-alpha-B9       ---------CATCGAGCCAGGTCTCCTTCCAACAGCCTCAGCAGCAATATCCATCAAGCC
Ta-alpha-B6       ---------CGTCAAGCCAGGTCTCCTTCCAACAGCCTCAGCAGCAATATCCATCAAGCC
Ta-alpha-B8       ---------CGTCAAGCCAGGTCTCCTTCCAACAGCCTCAGCAGCAATATCCATCAAGCC
Td-alpha-B6       ---------CGTCGAGCCAGGTCTCCTTCCAACAGCCTCAGCAGCAATATCCATCAAGCC
Td-alpha-B5       ---------CATCGAGCCAGGTCTCCTTCCAACAGCCTCAGCAGCAATATCCATCAAGCC
Td-alpha-B13      --------------AGCCAGTTCTCCTACCAGCAGCCCCATAACCAATATCCATCAGGCC
Ta-alpha-B22      AACAACGACCGTCGAGCGATGTCTCCTACCAGCAGCCTCAGCAAAAATATCCATCAGGCC

Ta-alpha-B14      AGGGCTTCTTCCAGCCATCTCAGCAAAACCCACAGGCCCAGGGCTTTGTCCAACCTCAGC
Ta-alpha-B15      AGGGCTTCTTCCAGCCATCTCAGCAAAACCCACAGGCCCAGGGCTTTGTCCAACCTCAGC
Ta-alpha-B16      AGGGCTTCTTCCAGCCATCTCAGCAAAACCCACAGGCCCAGGGCTTTGTCCAACCTCAGC
Ta-alpha-B17      AGGGCTTCTTCCAGCCATCTCAGCAAAACCCACAGGCCCAGGGCTTTGTCCAACCTCAGC
Ta-alpha-B18      AGGGCTTCTTCCAGCCATCTCAGCAAAACCCACAGGCCCAGGGCTTTGTCCAACCTCAGC
Ta-alpha-B11      AGGGCTTCTTCCAGCCATCTCAGCAAAACCCACAGGCCCAGGGCTTTGTCCAACCTCAGC
Ta-alpha-B12      AGGGCTCGTTCCAGCCATCTCAGCAAAACCCACAGGCCCAGGGCTTTGTCCAACCTCAGC
Ta-alpha-B13      AGGGCTCGTTCCAGCCATCTCAGCAAAACCCACAGGCCCAGGGCTTTGTCCAACCTCAGC
Ta-alpha-B23      AGGGCTCCTTCCAGCCATCTCAGCAAAACCCACAGGCCCAGGGCTTTGTCCAACCTCAAC
Td-alpha-B15      AGGGCTCCTTCCAGCCATCTCAGCAAAACCCACAGGCCCAGGGCTTTGTCCAACCTCAAC
Td-alpha-B10      AGGGCTCCTTCCAGCCATCTCAGCAAAACCCACAGGCCCAGGGCTTTGTCCAACCTCAAC
Ta-alpha-B21      ACGGCTCCTTCCAGCCATCTCAGCAAAACCCACAGACCCAGGGCTTTGTCCAACCTCAGC
Td-alpha-B9       ACGGCTCCTTCCAGCCATCTCAGCAAAACCCACAGACCCAGGGCTTTGTCCAACCTCAGC
```

```
Ta-alpha-B20      AGGGCTCCTTCCAGCCATCTGAGCAAAACCCACAGGCCGATGGCTTTGTCCAACCTCAGC
Td-alpha-B8       AGGGCTCCTTCCAGCCATCTCAGCAAAACCCACAGGCCCATGGCTTTGTCCAACCTCAGC
Td-alpha-B7       AGGGCTCCTTCCATCCATCTCAGCAAAACCCACAGGCCCATGGCTTTGTCCAACCTCAGC
Ta-alpha-B19      AGGGTTCCTTCCAGCCATCTCAGCAAAACCCACAGGCCCATGGCTTTGTCCAACCTCAGC
Ta-alpha-B1       AGGGCTCCTTCCAGCCATCTCAGCAGAACCCATAGGCCCAGGGCTCTGTCCAGTCTCAAC
Ta-alpha-B3       AGGGATCCTTCCAGCCATCTCAGCAGAACCCACAGGCCCAGGCCTCTGTCCAGCCTCAAC
Td-alpha-B1       AGGGCTCCTTCCAGCCATCTCAGCAGAACCCACAGGCCCAGGGCTCTGTCCAGTCTCAAC
Td-alpha-B2       AGGGCTCCTTCCAGCCATCTCAGCAGAACCCACAGGCCCAGGGCTTTGTCCAGTCTCAAC
Td-alpha-B3       AGGGCTCCTTCCAGCCATCTCAGCAAAACCCACAGCACCAGGGCTCTGTCCAGCCTCAAC
Td-alpha-B4       AGGGCTCCTTCCAGCCATCTCAGCAAAACCCACAGGACCAGGGCTCTGTCCAGCCTCAAC
Ta-alpha-B2       AGGGCTCCTTCCAGCCATCTCAGCAGAACCCATAGGCCTAGGGCTCTGTCCAGTCTCAAC
Ta-alpha-B7       AGGTCTCCTTCCAGCCATCTCAGCTAAACCCACAGGCTCAGGGCTCCGTCCAACCTCAAC
Ta-alpha-B10      AGGTCTTCTTCCAGCCATCTCAGCTAAACCCACAGGCTCAGGGCTCCGTCCAACCTCAAC
Ta-alpha-B9       AGGTCTCCTTCCAGCCATCTCAGCTAAACCCACAGGCTCAGGGCTCTGTCCAACCTCAAC
Ta-alpha-B6       AGGTCTCCTTCCAGCCATCTTAGCTAAACCCACAAGCTCAGGGCTCCGTCCAACCTCAAC
Ta-alpha-B8       AGGTCTCCTTCCAGCCATCTCAGCTAAACCCACAAGCTCAGGGCTCCGTCCAACCTCAAC
Td-alpha-B6       AGGTCTCCTTCCAGCCATCTCAGCTAAACCCACAGGCTCAGGGCTCTGTCCAACCTCAAC
Td-alpha-B5       AGGTCTCCTTCCAGCCATCTCAGCTATACCCACAGGCTCAGGGCTCTGTCCAACCTCAAC
Td-alpha-B13      AAGGCTCCTTCCAGCCATCTCAGCAAAACCCACAGGCCTAGGGCTCTGTCCGACCTCAAC
Ta-alpha-B22      AGGGCTCCTTCCAGCCATCTCAGCAAAACCCACAGGCCCAGGGCTTTGTCCAACCTCAGC

Ta-alpha-B14      AACTGCCGCAGTTCGAGGAAATAAGGAACCTAGCGCTGCAGACGCTACCAGCAATGTGCA
Ta-alpha-B15      AACTGCCGCAGTTCCAGGAAATAAGGAACCTAGCGCTGCAGACGCTACCAGCAATGTGCA
Ta-alpha-B16      AACTGCCGCAGTTCGAGGAAATAAGGAACCTAGCGCTGCAGACGCTACCAGCAATGTGCA
Ta-alpha-B17      AACTGCCGCAGTTCGAGGAAATAAGGAACCTAGCGCTGCAGACGCTACCAGCAATGTGCA
Ta-alpha-B18      AACTGCCGCAGTTCGAGGAAATAAGGAACCTAGCGCTGCAGACGCTACCAGCAATGTGCA
Ta-alpha-B11      AACTGCCGCAGTTCGAGGAAATAAGGAACCTAGCGCTGCAGACGCTACCAGCAATGTGCA
Ta-alpha-B12      AACTGCCGCAGTTCGAGGAAATAAGGAACCTAGCGCTACAGACGCTACCAGCAATGTGCA
Ta-alpha-B13      AACTGCCGCAGTTCGAGGAAATAAGGAACCTAGCGCTACAGACGCTACCAGCAATGTGCA
Ta-alpha-B23      AACTGCCCCAGTTCGAGGAAATAAGGAACCTAGCACTGCAGACGCTACCTGCAATGTGCA
Td-alpha-B15      AACTGCCCCAGTTCGAGGAAATAAGGAACCTAGCGCTGCAGACGCTACCTGCAATGTGCA
Td-alpha-B10      AACTGCCCCAGTTCGAGGAAATAAGGAACCTAGCGCTGCAGACGCTACCTGCAATGTGCA
Ta-alpha-B21      AACTGCTGCAGTTTGAGGAAATAAGGAACGTAGCGCTGCAGACACTACCAGCAATGTGCA
Td-alpha-B9       AACTGCCGCAGTTTGAGGAAATAAGGAACGTAGCGCTGCAGACACTACCAGCAATGTGCA
Ta-alpha-B20      AACTGCCGCAGTTTGAGTAAATAAGGAACCTAGCGCTGCAGACACTACCAGCAATGTGCA
Td-alpha-B8       AACTGCCGCAGTTTGAGGAAATAAGGAACCTAGCGCTGCAGACACTACCAGCAATGTGCA
Td-alpha-B7       AACTGCCGCAGTTTGAGGAAATAAGGAACCTAGCGCTGCAGACACTACCAGCAATGTGCA
Ta-alpha-B19      AACTGCCACAGTTTGAGGAAATAAGGAACCTAGCGCTACAGACACTACCAGCAATGTGCA
Ta-alpha-B1       AACTTCCCCAGTTCGAGGAAATAAGGAACCTAGTGCTGCAGACGCTACCGGCAATGTGCA
Ta-alpha-B3       AACTTCCCCAGTTCGAGGAAATAAGGAATCTAGCACGGCAGACGCTACCGGCAATGTGCA
Td-alpha-B1       AACTTCCCCAGTTCGAGGAAATAAGGAACCTAGCGCTGCAGACGCTACCGGTAATGTGCA
Td-alpha-B2       AACTTCCCCAGTTCGAGGAAATAAGGAACCTAGCACTGCAGACGCTACCGGCAATGTGCA
Td-alpha-B3       AACTGCCCCAGATCGAGGAAATAAGGAACCTAGCGCTGCAGACGCTACCGGCAATGTGCA
Td-alpha-B4       AACTGCCCCAGATCGAGGAAATAAGGAACCTAGCGCTGCAGACGCTACCGGCAATGTGCA
Ta-alpha-B2       AACTTCCCCAGTTCGAGGAAATAAGGAACCTAGCGCTGCAGACGCTACCGGCAATGTGCA
Ta-alpha-B7       AACTGCCCCAGTTCGCGGAAATAAGGAACCTAGCGCTACAGACGCTACCTGCAATGTGCA
Ta-alpha-B10      AACTGCCCCAGTTCGCGGAAATAAGGAACCTAGCGCTACAGACGCTACCTGCAATGTGCA
Ta-alpha-B9       AACTGCCCCAGTTCGCGGAAATAAGGAACCTAGCGCTACAGACGCTACCTGCAATGTGCA
Ta-alpha-B6       AACTGCCCCAGTTCGCGGAAATAAGGAACCTAGCGCTACAGACGCTACCTGCAATGTGCA
Ta-alpha-B8       AACTGCCCCAGTTCGCGGAAATAAGGAACCTAGCGCTACAGACGCTACCTGCAATGTGCA
Td-alpha-B6       AACTACCCCAGTTCGCGGAAATAAGGAACATAGCGCTACAGACGCTACCTGCAATGTGCA
Td-alpha-B5       AACTGCCCCAGTTCGCGGAAATAAGGAACCTAGCGCTACAGACGCTACCTGCAATGTGCA
Td-alpha-B13      AACTGCCCCAGTTCGAGGAAATAAGGAACCTAGCGCTGTAGACTCTACCAACAATGTGCA
Ta-alpha-B22      AACTGCCCCAATTCGAGAAAATAAGGAACCTAGCATTGCAGACGCTACCAGCAATGTGCA

Ta-alpha-B14      ATGTCTACATCCCTCCATATTGCTCGACCACCATTGCGCCATTTGGCATCATGAGTACTA
Ta-alpha-B15      ATGTCTACATCCCTCCATATTGCTCGACCACCATTGCGCCATTTGGCATCATGAGTACTA
Ta-alpha-B16      ATGTCTACATCCCTCCATATTGCTCGACCACCATTGCGCCATTTGGCATCATGAGTACTA
Ta-alpha-B17      ATGTCTACATCCCTCCATATTGCTCGACCACCATTGCGCCATTTGGCATCATGAGTACTA
```

```
Ta-alpha-B18      ATGTCTACATCCCTCCATATTGCTCGACCACCATTGCGCCATTTGGCATCATGAGTACTA
Ta-alpha-B11      ATGTCTACATCCCTCCATATTGCTCGACCACCATTGCGCCATTTGGCATCATGAGTACTA
Ta-alpha-B12      ATGTCTACATCCCTCCATATTGCTCGACCACCATTGCGCCATTTGGCATCATGAGTACTA
Ta-alpha-B13      ATGTCTACATCCCTCCATATTGCTCGACCACCATTGCGCCATTTGGCATCATGAGTACTA
Ta-alpha-B23      ATGTCTACATCCCTCCATATTGCTCGACCACCATTGTGCCATTTGGCATCATCGGTACTA
Td-alpha-B15      ATGTCTACATCCCTCCATATTGCTCGACCACCATTGTGCCATTTGGCATCATCGGTACTA
Td-alpha-B10      ATGTCTACATCCCTCCATATTGCTCGACCACCATTGTGCCATTTGGCATCATCGGTACTA
Ta-alpha-B21      ATGTCTACATCCCTCCATATTGCTCGACCACCATTGCGCCATTTGGCATCGTGAGTACTA
Td-alpha-B9       ATGTCTACATCCCTCCATATTGCTCGACCACCATTCCGCCATTTGGCATCGTGAGTACTA
Ta-alpha-B20      ATGTCTACATCCCTCCATATTGCTCGACCACCATTGCGCCATTTGGCATCGTGAGTACTA
Td-alpha-B8       ATGTCTACATCCCTCCATATTGCTCGACCACCATTGCGCCATTTGGCATCGTGAGTACTA
Td-alpha-B7       ATGTCTACATCCCTCCATATTGCTCGACCACCATTGCGCCATTTGGCATCGTGAGTACTA
Ta-alpha-B19      ATGTCTACATCCCTCCATATTGCTCGACCACCATTGCGCCATTTGGCATCGTGAGTACTA
Ta-alpha-B1       ATGTCTACATCCCTCCATATTGCTCGACCACCATTGCGCCATCTGGCATCTTCGGTACTA
Ta-alpha-B3       ATGTCTACATCCCTCCATATTGCTCGACCACCATTGCGCCATCTGGCATCTTTGGTACTA
Td-alpha-B1       ATGTCTACATCCCTCCATATTGCTCGACCACCATTGCGCCATCTGGCATCTTCGGTACTA
Td-alpha-B2       ATGTCTACATCCCTGCATATTGCTCGACCACCATTGCGCCATCTGGCATCTTCAGTTCTA
Td-alpha-B3       ATGTCTACATCCCTCCATATTGCTCGACCACCATTGCGCCATTTGGCATCCTCGGTACTA
Td-alpha-B4       ATGTCTACATCCCTCCATATTGCTCGATAACCATTGCGCCATTTGGCATCCTCGGTACTA
Ta-alpha-B2       ATGTCTACATCCCTCCATATTGCTCGACCACCATTGCGCCATCTGGCATCTTCGGTACTA
Ta-alpha-B7       ATGTCTACATCCCTCCACATTGCTCGACCACCATTGCGCCATTTGGCATCTTTGGTACCA
Ta-alpha-B10      ATGTCTACATCCCTCCACATTGCTCGACCACCATTGCGCCATTTGGCATCTTTGGTACCA
Ta-alpha-B9       ATGTCTACATCCCTCCACATTGCTCGACCACCATTGCGCCATTTGGCATCTTTGGTACCA
Ta-alpha-B6       ATGTCTACATCCCTCCACATTGCTCGACCACCATTGCGCCATTTGGCATCTTTGGTACCA
Ta-alpha-B8       ATGTCTACATCCCTCCACATTGCTCGACCACCATTGCGCCATTTGGCATCTTTGGTACCA
Td-alpha-B6       ATGTCTACATCCCTCCACATTGCTCGACCACCATTGCGCCATTTGGCATCTTCGGTACCA
Td-alpha-B5       ATGTCTACATCCCTCCACATTGCTCGACCACCATTGCACCATTTGGCATCTTCGGTACCA
Td-alpha-B13      ATGTCTACATCCCTCCATATTGCTCGGCCACCATTGCGCCATTTGGCATCGTCGGTAGTA
Ta-alpha-B22      ATGTCTACATCCCTCCATATT-CTCGACCACCATTGCGCCATTTGGCATCGTCGGCACTA

Ta-alpha-B14      ACTGA
Ta-alpha-B15      ACTGA
Ta-alpha-B16      ACTGA
Ta-alpha-B17      ACTGA
Ta-alpha-B18      ACTGA
Ta-alpha-B11      ACTGA
Ta-alpha-B12      ACTGA
Ta-alpha-B13      ACTGA
Ta-alpha-B23      ACTGA
Td-alpha-B15      ACTGA
Td-alpha-B10      ACTGA
Ta-alpha-B21      ACTGA
Td-alpha-B9       ACTGA
Ta-alpha-B20      ATTGA
Td-alpha-B8       ACTGA
Td-alpha-B7       ACTGA
Ta-alpha-B19      ACTGA
Ta-alpha-B1       ACTGA
Ta-alpha-B3       ACTGA
Td-alpha-B1       ACTGA
Td-alpha-B2       AATGA
Td-alpha-B3       ACTGA
Td-alpha-B4       ACTGA
Ta-alpha-B2       ACTGA
Ta-alpha-B7       ACTGA
Ta-alpha-B10      ACTGA
Ta-alpha-B9       ACTGA
Ta-alpha-B6       ACTGA
Ta-alpha-B8       ACTGA
```

```
Td-alpha-B6       ACTGA
Td-alpha-B5       ACTGA
Td-alpha-B13      ACTGA
Ta-alpha-B22      ACTGA
```
