# Supplementary material for: Rapid evolution of α-gliadin gene family revealed by analyzing Gli-2 locus regions of wild emmer wheat
Source: Funct Integr Genomics. 2019 Jun 13;19(6):993–1005. doi: 10.1007/s10142-019-00686-z (PMC6797660; doi:10.1007/s10142-019-00686-z)
Supplement: Supplementary file 6 — (PDF 75 kb) [file 10142_2019_686_MOESM6_ESM.pdf]

Table S1. Wild emmer sequence scaffolds in the α-gliadin locus regions

	Scaffolds	Size (bp)
A genome	scffold43817	2803334
	scffold108470	765716
	scffold48495	301868
	scaffold46758	131996
	scaffold58108	2539422
B genome	scaffold13531-1	2692525
