# Supplementary material for: Rapid evolution of α-gliadin gene family revealed by analyzing Gli-2 locus regions of wild emmer wheat
Source: Funct Integr Genomics. 2019 Jun 13;19(6):993–1005. doi: 10.1007/s10142-019-00686-z (PMC6797660; doi:10.1007/s10142-019-00686-z)
Supplement: Supplementary file 7 — (PDF 101 kb) [file 10142_2019_686_MOESM7_ESM.pdf]

| Table S2 Annatation of Wild Emmer alpha gliadin gene region | | | |
|---|---|---|---|
| **Annotation** | **Gene symbol** | **Start** | **End** |
| **A genome** | | | |
| Glucan endo-1,3-beta-glucosidase 3 | Td-GLR-A1 | 1 | 3586 |
| Glucan endo-1,3-beta-glucosidase 3 | Td-GLR-A2 | 23910 | 27576 |
| alpha gliadin | Td-α-A1 | 66882 | 67805 |
| Glucan endo-1,3-beta-glucosidase 3 | Td-GLR-A3 | 116117 | 119998 |
| alpha gliadin | Td-α-A2 | 288446 | 289324 |
| alpha gliadin | Td-α-A3 | 485701 | 486485 |
| alpha gliadin | Td-α-A4 | 579661 | 580519 |
| alpha gliadin | Td-α-A5 | 688247 | 689105 |
| alpha gliadin | Td-α-A6 | 768290 | 768872 |
| alpha gliadin | Td-α-A7 | 846092 | 846787 |
| alpha gliadin | Td-α-A8 | 1181702 | 1182550 |
| alpha gliadin | Td-α-A9 | 1199905 | 1200750 |
| alpha gliadin | Td-α-A10 | 1246292 | 1247125 |
| alpha gliadin | Td-α-A11 | 1384965 | 1385801 |
| alpha gliadin | Td-α-A12 | 1530235 | 1531079 |
| alpha gliadin | Td-α-A13 | 1565823 | 1566668 |
| alpha gliadin | Td-α-A14 | 1655706 | 1656599 |
| alpha gliadin | Td-α-A15 | 1728431 | 1729300 |
| alpha gliadin | Td-α-A16 | 1807910 | 1808365 |
| alpha gliadin | Td-α-A17 | 1829818 | 1830684 |
| alpha gliadin | Td-α-A18 | 1891268 | 1892112 |
| alpha gliadin | Td-α-A19 | 2334062 | 2335349 |
| alpha gliadin | Td-α-A20 | 2411571 | 2412431 |
| alpha gliadin | Td-α-A21 | 2458854 | 2459711 |
| alpha gliadin | Td-α-A22 | 2513911 | 2514769 |
| alpha gliadin | Td-α-A23 | 2540755 | 2541609 |
| alpha gliadin | Td-α-A24 | 2600291 | 2601139 |
| Glucan endo-1,3-beta-glucosidase 3 | Td-GLR-A4 | 2890723 | 2896285 |
| **B Genome** | | | |
| Glucan endo-1,3-beta-glucosidase 3 | Td-GLR-B1 | 1 | 3731 |
| Glucan endo-1,3-beta-glucosidase 3 | Td-GLR-B2 | 54928 | 58529 |
| alpha gliadin | Td-α-B1 | 109390 | 110334 |
| alpha gliadin | Td-α-B2 | 155497 | 156492 |
| alpha gliadin | Td-α-B3 | 161272 | 162294 |
| alpha gliadin | Td-α-B4 | 178698 | 179666 |
| Glucan endo-1,3-beta-glucosidase 3 | Td-GLR-B3 | 215220 | 218859 |
| alpha gliadin | Td-α-B5 | 237980 | 238906 |
| alpha gliadin | Td-α-B6 | 266320 | 267237 |
| alpha gliadin | Td-α-B7 | 563035 | 585104 |
| alpha gliadin | Td-α-B8 | 591900 | 614063 |
| alpha gliadin | Td-α-B9 | 632272 | 633084 |

| | | | |
|---|---|---|---|
| alpha gliadin | Td-α-B10 | 764551 | 765088 |
| alpha gliadin | Td-α-B11 | 769182 | 769412 |
| alpha gliadin | Td-α-B12 | 790309 | 790977 |
| alpha gliadin | Td-α-B13 | 811179 | 811553 |
| alpha gliadin | Td-α_B14 | 838867 | 839012 |
| alpha gliadin | Td-α-B15 | 860314 | 861198 |
| alpha gliadin | Td-α-B16 | 865290 | 874116 |
| Glucan endo-1,3-beta-glucosidase 3 | Td-GLR-B4 | 908912 | 912488 |
